# Randomized crossover trial of a modified ketogenic diet in Alzheimer’s disease

**DOI:** 10.1186/s13195-021-00783-x

**Published:** 2021-02-23

**Authors:** Matthew C. L. Phillips, Laura M. Deprez, Grace M. N. Mortimer, Deborah K. J. Murtagh, Stacey McCoy, Ruth Mylchreest, Linda J. Gilbertson, Karen M. Clark, Patricia V. Simpson, Eileen J. McManus, Jee-Eun Oh, Satish Yadavaraj, Vanessa M. King, Avinesh Pillai, Beatriz Romero-Ferrando, Martijn Brinkhuis, Bronwyn M. Copeland, Shah Samad, Shenyang Liao, Jan A. C. Schepel

**Affiliations:** 1 Department of Neurology, Waikato Hospital, Hamilton, New Zealand; 2 Faculty of Psychology and Neuroscience, Maastricht University, Maastricht, The Netherlands; 3 Healthy Kitchen Christchurch Ltd, Hamilton, New Zealand; 4 Department of Dietetics Services, Waikato Hospital, Hamilton, New Zealand; 5 LINC Mental Health Services, Tauranga Hospital, Tauranga, New Zealand; 6 Department of Statistics, University of Auckland, Auckland, New Zealand; 7 Mental Health Services for Older People, Waikato Hospital, Hamilton, New Zealand; 8 Mental Health Services for Older People, Tauranga Hospital, Tauranga, New Zealand; 9 Older Persons and Rehabilitation Service, Waikato Hospital, Hamilton, New Zealand

**Keywords:** Alzheimer’s disease, Ketogenic diet, Randomized crossover trial, Cognition, Daily function, Quality of life

## Abstract

**Background:**

Brain energy metabolism is impaired in Alzheimer’s disease (AD), which may be mitigated by a ketogenic diet. We conducted a randomized crossover trial to determine whether a 12-week modified ketogenic diet improved cognition, daily function, or quality of life in a hospital clinic of AD patients.

**Methods:**

We randomly assigned patients with clinically confirmed diagnoses of AD to a modified ketogenic diet or usual diet supplemented with low-fat healthy-eating guidelines and enrolled them in a single-phase, assessor-blinded, two-period crossover trial (two 12-week treatment periods, separated by a 10-week washout period). Primary outcomes were mean within-individual changes in the Addenbrookes Cognitive Examination - III (ACE-III) scale, AD Cooperative Study - Activities of Daily Living (ADCS-ADL) inventory, and Quality of Life in AD (QOL-AD) questionnaire over 12 weeks. Secondary outcomes considered changes in cardiovascular risk factors and adverse effects.

**Results:**

We randomized 26 patients, of whom 21 (81%) completed the ketogenic diet; only one withdrawal was attributed to the ketogenic diet. While on the ketogenic diet, patients achieved sustained physiological ketosis (12-week mean beta-hydroxybutyrate level: 0.95 ± 0.34 mmol/L). Compared with usual diet, patients on the ketogenic diet increased their mean within-individual ADCS-ADL (+ 3.13 ± 5.01 points, *P* = 0.0067) and QOL-AD (+ 3.37 ± 6.86 points, *P* = 0.023) scores; the ACE-III also increased, but not significantly (+ 2.12 ± 8.70 points, *P* = 0.24). Changes in cardiovascular risk factors were mostly favourable, and adverse effects were mild.

**Conclusions:**

This is the first randomized trial to investigate the impact of a ketogenic diet in patients with uniform diagnoses of AD. High rates of retention, adherence, and safety appear to be achievable in applying a 12-week modified ketogenic diet to AD patients. Compared with a usual diet supplemented with low-fat healthy-eating guidelines, patients on the ketogenic diet improved in daily function and quality of life, two factors of great importance to people living with dementia.

**Trial registration:**

This trial is registered on the Australia New Zealand Clinical Trials Registry, number ACTRN12618001450202. The trial was registered on August 28, 2018.

**Supplementary Information:**

The online version contains supplementary material available at 10.1186/s13195-021-00783-x.

## Introduction

Despite extensive efforts towards prevention and remediation, dementia remains an urgent public health priority, affecting over 50 million people worldwide [1]. The majority of people with dementia have Alzheimer’s disease (AD), a disorder that characteristically results in progressive cognitive and functional decline [2]. Supportive care remains the mainstay of treatment, and new strategies are needed.

Brain energy metabolism is impaired in AD. Compared with healthy controls, people with AD display lower levels of brain insulin signaling and fewer brain insulin receptors, culminating in brain insulin resistance [3, 4]. PET studies demonstrate a 20–25% deficiency in cerebral glucose metabolism [5]. AD neurons also exhibit diminished numbers of mitochondria, many of which show reduced citric acid cycle and respiratory chain activity, culminating in decreased energy production [6].

Ketogenic diets can theoretically mitigate impaired brain energy metabolism in AD, leading to improved cognition, daily function, or quality of life. Ketogenic diets are high-fat, low-carbohydrate diets that shift the body towards fat metabolism. Neurons cannot metabolize fats directly, but the liver converts fats into ketones, which can serve as a major neuron energy source [7]. During a typical western diet, the concentration of the primary blood ketone, beta-hydroxybutyrate, supplies less than 5% of brain energy requirements and its blood concentration rarely exceeds 0.5 mmol/L. By contrast, a ketogenic diet induces a state of “physiological ketosis” in which beta-hydroxybutyrate provides a greater contribution to brain energy metabolism and its blood concentration exceeds 0.5–0.6 mmol/L. [8, 9] Compared with glucose, ketones produce more energy per unit oxygen [10]. Cerebral ketone metabolism remains normal in AD and can potentially compensate for brain insulin resistance and deficient glucose metabolism [11]. Ketogenic diets also typically upregulate mitochondria biogenesis and induce expression of genes associated with the citric acid cycle and respiratory chain, thus increasing neuron energy production [12].

To date, two clinical trials have examined the symptomatic effects of a ketogenic diet in AD. A single-arm study examined the impact of a 12-week ketogenic diet in 15 AD patients [13]. The 11 completers improved their cognitive scores, but the lack of a control group meant that additional contributing factors could not be ruled out. A second ongoing randomized controlled trial has provided preliminary data on the impact of a 12-week Modified Atkins diet versus a recommended diet in 14 patients with mild cognitive impairment or AD [14]. The most adherent patients improved their memory scores, but overall adherence was only fair, and function did not improve.

On this background, we conducted a randomized crossover trial to determine whether a 12-week modified ketogenic diet was well-tolerated and improved cognition, daily function, or quality of life in a hospital clinic of AD patients.

## Materials and methods

### Trial design

This was a single-phase, assessor-blinded, two-period randomized crossover trial conducted at Waikato Hospital, a tertiary hospital in Hamilton, New Zealand. The trial was approved by the Waikato Maori Consultation Research Review Committee and Health and Disability Ethics Committee of New Zealand.

Patients and trial partners attended a screening visit in July 2019 and a diet instruction visit in August 2019. In September 2019, patients were randomized (1:1 allocation) to a modified ketogenic diet (intervention diet) or their usual diet supplemented with low-fat healthy-eating guidelines and optional recipes (control diet). The crossover design specified two 12-week treatment periods separated by a 10-week washout period during which patients resumed their usual diet (cognitive changes induced by a ketogenic diet in AD return to baseline after 1 month) [13]. For each treatment period, assessments were made at baseline, week 6, and week 12.

### Patients

The trial was advertised in newspapers and regional dementia organizations. Volunteers attended a 2-h screening visit that included a description of the trial, medical history, evaluation of current diagnostic criteria for probable AD [2], dementia severity rating scale [15], AD informed consent questionnaire (with the word “medication” replaced with “diet”) [16], geriatric depression scale (short form) [17], Hachinski ischemia scale [18], body mass index calculations, and (assuming consent capacity) written informed consent from both patient and trial partner.

Eligible patients were 50 to 90 years of age, satisfied the revised NINCDS-ADRDA criteria for probable AD (confirmed by a neurologist or geriatrician), had a dementia severity rating scale score < 19, a body mass index > 18.5, and a cohabiting trial partner willing to (at least partly) partake in a ketogenic diet. Exclusion criteria included moderate or severe depression (geriatric depression scale score > 8), substantial cerebrovascular disease (Hachinski ischemia scale score > 4), a change in acetylcholinesterase inhibitor dose within the past 6 weeks, and a concurrent medical or psychiatric disorder judged likely to create difficulty in completing the trial. Patients also had recent (within 1 year) blood investigations (cell count, electrolytes, creatinine, liver function, thyroid-stimulating hormone, B12, and folate) in the normal range, as well as recent (within 2 years) CT or MRI brain imaging showing no ischemic changes beyond age-appropriate leukoariosis (missing investigations for otherwise eligible patients were performed after screening).

### Diets

Following screening, eligible patients and trial partners attended a 1-h diet instruction visit and were shown how to use a complimentary blood glucose and ketone (beta-hydroxybutyrate) monitor (FreeStyle Neo, Abbott Diabetes Care, Whitney, UK), complete a 3-day (two weekdays, one weekend day) food record, and follow the diet plans. Blood was taken for apolipoprotein E genotyping.

Both diet plans contained guidelines, space to record daily (bedtime) blood glucose and ketone levels, and recipes (for full plans, see Supplementary Material). Patients on the ketogenic diet were instructed to eat all meals from the plan (unless they attended a social event, in which case meal advice was given), with numerous recipe options providing an average macronutrient ratio of 58% fat (26% saturated, 32% non-saturated), 29% protein, 7% fibre, and 6% net carbohydrate by weight. Main dietary constituents were green vegetables, meats, eggs, nuts, seeds, creams, and natural oils. The “usual” diet plan contained optional low-fat recipes in accordance with New Zealand healthy-eating guidelines, providing an average ratio of 11% fat (3% saturated, 8% non-saturated), 19% protein, 8% fibre, and 62% net carbohydrate by weight. Constituents were mainly green and root vegetables, meats, legumes, whole grains, and fruits. Both diets were supplemented by a daily multivitamin (Multivitamin and Mineral Boost, Clinicians Ltd., Auckland, New Zealand).

### Randomization and blinding

Following stratification by dementia severity rating scale score and body mass index (below baseline mean, above baseline mean), the trial statistician randomized patients (1:1 allocation, block size of four) to the intervention or control diet using SAS statistical software (SAS Institute, Cary, USA).

Diet-related discussion between assessors and patients (or trial partners) was prohibited throughout the trial. To prevent detection of acetone breath, a fragrance-diffusing scent (Naturals Diffuser, The Aromatherapy Co., Auckland, New Zealand) was placed between assessors and patients (or trial partners) at every assessment.

### Assessments

Patients and trial partners attended three 1-h assessments over each 12-week treatment period. A baseline assessment was made during the week prior to commencing the treatment period, followed by assessments during weeks 6 and 12. Assessments evaluated patient cognition, daily function, and quality of life; patients and trial partners were evaluated by the same assessor at baseline and week 12, on the same weekday and hour of the day. Cognition was assessed using the Addenbrookes Cognitive Examination - III (ACE-III) scale, administered by an ACE-III-trained neurologist, neuropsychologist, psychiatrist, or geriatrician (New Zealand version A at baseline, version B in week 6, and version C in week 12). The ACE-III assesses 19 activities pertaining to five cognitive domains: attention, memory, fluency, language, and visuospatial ability (scores range from 0 to 100, with higher numbers indicating better cognition) [19]. It has been objectively validated, with high levels of correlation shown between domain scores and performance on standard neuropsychological measures [19], and is the primary cognitive assessment battery used in our hospital. The AD Cooperative Study - Activities of Daily Living (ADCS-ADL) inventory was administered to the trial partner. The ADCS-ADL assesses 23 items (scores range from 0 to 78, with higher numbers indicating better daily function) and has good test-retest reliability [20]. The Quality of Life in AD (QOL-AD) questionnaire was also administered to the trial partner. The QOL-AD assesses 13 items (scores range from 13 to 52, with higher numbers indicating better quality of life) and has good test-retest reliability [21]. Assessments included body weight measurements and blood tests for glycosylated haemoglobin (HbA1C), triglycerides, high-density lipoprotein (HDL), low-density lipoprotein (LDL), and total cholesterol. An adverse effects questionnaire was given to both patient and trial partner by a nutrition specialist at weeks 6 and 12 within each treatment period. The week 12 assessment for the second treatment period included a questionnaire asking whether the patient or trial partner would continue the ketogenic diet after completing the trial.

### Washout period

During the first treatment period, patients and trial partners were prohibited from making copies of the diet plans. At the week 12 assessment of the first treatment period, all plans were returned and a 10-week washout period ensued. During this interval, patients and trial partners were repeatedly reminded to return to their usual diet (all trial partners vouched that the patients complied).

### Educational programme

The lead investigator and nutrition specialists delivered an educational programme, consisting of two global e-mails per week and a 10-min video posted on the trial website every weekend. The programme provided information about facts and misconceptions relating to ketogenic and low-fat diets. Both diet approaches were consistently presented as potentially healthy and all patients were encouraged to eat until satiation.

### Primary and secondary outcomes

Primary outcomes were mean within-individual changes in cognition (ACE-III), daily function (ADCS-ADL), and quality of life (QOL-AD) from baseline to week 12. Secondary outcomes included mean within-individual changes in cardiovascular risk factors (weight, body mass index, HbA1C, triglycerides, HDL, LDL, and total cholesterol) from baseline to week 12.

### Statistical analysis

Sample size calculations considered that a 5-point change on the ACE-III was clinically meaningful (generally agreed by physicians at our hospital), while a 2-point change on the ADCS-ADL was considered clinically meaningful as this degree of change represents a gain or loss of independence in one domain of daily function [22]. A 3-point change on the QOL-AD was considered clinically meaningful as this represents a change from very poor to excellent, or vice versa, in one domain of quality of life [23]. For each outcome, we conservatively assumed a within-individual standard deviation equal to 50% of the between-individual standard deviation, resulting in a calculated conversion factor of 10 for the crossover design [24]. To obtain 90% power at a significance level of 0.05, 18 patients were needed to complete both diets to detect an ACE-III change of 5 ± 10 points, an ADCS-ADL change of 2 ± 4 points, or a QOL-AD change of 3 ± 6 points. Since the only previous study involving a ketogenic diet in patients with uniform diagnoses of AD showed a 27% dropout rate over 12 weeks [13], we sought to recruit 25 to 30 patients.

Given the pre-trial uncertainty regarding data distribution, all outcomes were analysed using (nonparametric) Wilcoxon signed-rank tests. To check for a period effect, we performed Mann-Whitney *U* tests on the baseline means for all comparison groups (by treatment period, by treatment sequence, and for all patients). Statistical tests were two-tailed and considered an alpha of 5% as statistically significant. Data are presented as mean ± standard deviation unless stated otherwise.

We analysed primary and secondary outcomes using data from all randomized patients, with missing data imputed using regression imputation. We also performed an efficacy analysis on primary outcomes using data solely from “completers” who remained on protocol for both treatment periods and achieved sustained physiological ketosis (12-week mean beta-hydroxybutyrate level ≥ 0.6 mmol/L) during the ketogenic diet intervention.

## Results

### Patient flow

**Fig. 1:**
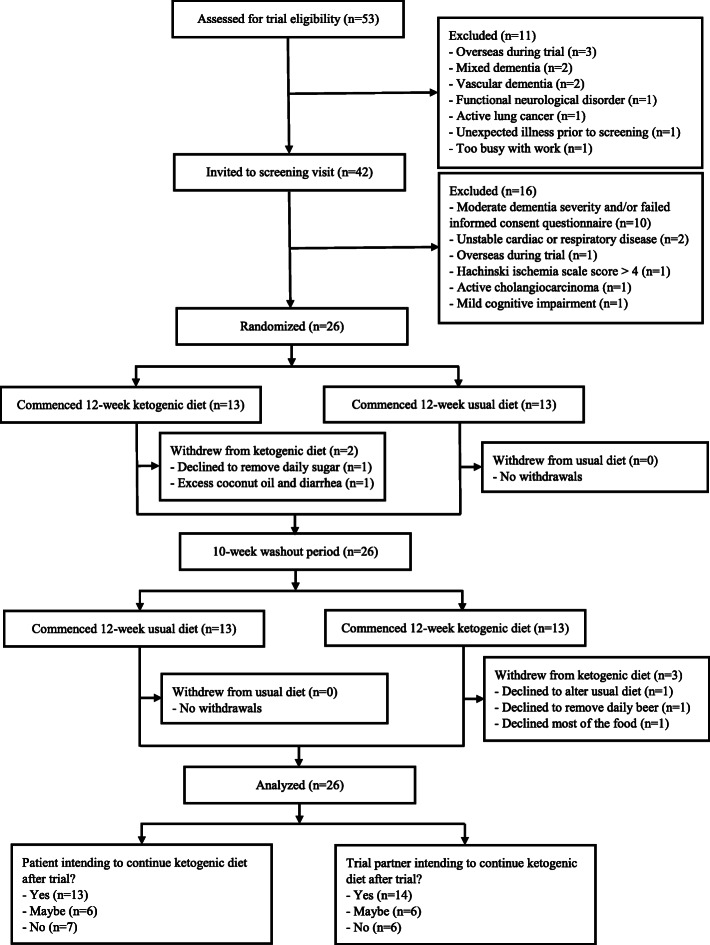
Patient flow, showing all exclusions and withdrawals

Patient flow is shown in Fig. 1. Of the 26 patients, 21 (81%) completed the ketogenic diet and all completed their usual diet supplemented with low-fat healthy-eating guidelines. Four of the five withdrawals from the ketogenic diet resulted from the patient’s refusal to alter their diet, creating conflict with the trial partner. Only one withdrawal was attributed to ill effects of the ketogenic diet (this patient increased their coconut oil intake beyond the recommended amount, resulting in diarrhoea). At the week 12 assessment of the second treatment period, 13 (50%) of the patients and 14 (54%) of the trial partners stated that they intended to continue the ketogenic diet after the trial.

### Baseline characteristics

Baseline characteristics for all randomized patients are shown in Table 1. The sole significant difference between the two treatment sequence groups was a larger proportion of apolipoprotein E4 carriers in the usual-ketogenic diet sequence (chi-square test: *P* = 0.0039).

**Table 1:** Baseline characteristics for all randomized patients

Characteristic	Ketogenic-usual diet (***n*** = 13)	Usual-ketogenic diet (***n*** = 13)	All patients (***n*** = 26)
**Age (years)**	68.0 ±5.4 (range, 57–77)	71.7 ± 6.2 (range, 61–79)	69.8 ± 6.0 (range, 57–79)
**Sex (male)**	10 (77%)	6 (46%)	16 (62%)
**Education (years)**	13.2 ± 3.6	11.5 ± 1.1	12.3 ± 2.7
**Ethnicity**			
**European**	11 (85%)	11 (85%)	22 (85%)
**Maori**	1 (8%)	1 (8%)	2 (8%)
**Cook Islander**	0	1 (8%)	1 (4%)
**Indian**	1 (8%)	0	1 (4%)
**Screening tests**			
**Dementia severity rating scale**	11.8 ± 5.2	12.4 ± 4.8	12.1 ± 4.9
**Geriatric depression scale**	2.2 ± 1.7	2.2 ± 2.0	2.2 ± 1.8
**Hachinski ischemia scale**	2.1 ± 1.4	1.4 ± 1.0	1.7 ± 1.3
**Body mass index**	27.8 ± 5.9	26.6 ± 2.7	27.2 ± 4.5
**Apolipoprotein E genotype**			
**Noncarrier (E2/3)**	2 (15%)	1 (8%)	3 (12%)
**Noncarrier (E3/3)**	6 (46%)	0	6 (23%)
**Heterozygote (E3/4)**	4 (31%)	11 (85%)	15 (58%)
**Homozygote (E4/4)**	1 (8%)	1 (8%)	2 (8%)
**Comorbidities relevant to ketogenic diet**			
**Type 2 diabetes**	0	2 (15%)	2 (8%)
**Ischemic heart disease**	2 (15%)	1 (8%)	3 (12%)
**Cholecystectomy**	0	1 (8%)	1 (4%)
**History of urinary calculus**	1 (8%)	2 (15%)	3 (12%)
**History of gout**	0	2 (15%)	2 (8%)
**Medications relevant to cognition**			
**Donepezil**	4 (31%)	4 (31%)	8 (31%)
**Beta-blocker**	4 (31%)	1 (8%)	5 (19%)
**Statin**	2 (15%)	2 (15%)	4 (15%)
**Benzodiazepine**	1 (8%)	1 (8%)	2 (8%)
**No medications at all**	3 (23%)	4 (31%)	7 (27%)
**Usual diet (by weight)**			
**Fat**	21%	21%	21%
**Saturated**	11%	12%	12%
**Unsaturated**	10%	9%	9%
**Protein**	24%	24%	24%
**Fibre**	7%	8%	8%
**Net carbohydrate**	48%	47%	47%
**Usual diet (by energy intake)**			
**Fat**	37%	37%	37%
**Protein**	19%	19%	19%
**Carbohydrate**	44%	44%	44%

Except for % variables, values are presented as mean ± standard deviation

Due to round-off, some percent variables may add up to slightly over 100%

### Adherence

**Fig. 2:**
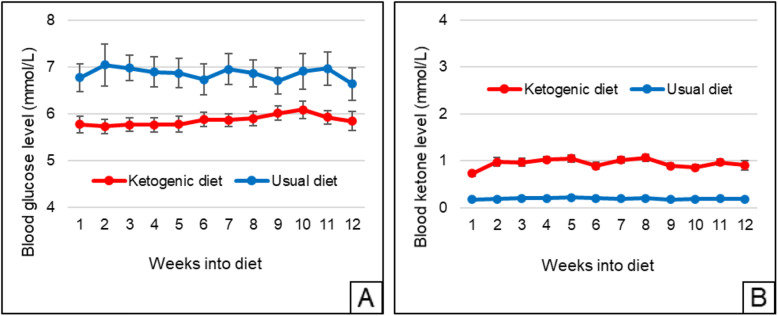
Mean weekly blood (**a**) glucose and (**b**) ketone (beta-hydroxybutyrate) levels for all randomized patients (*n* = 26 for ketogenic diet, *n* = 26 for usual diet). For patients with weeks containing partial data, the days containing data were used to calculate the weekly mean. Regarding withdrawals, all data up to the point of withdrawal are shown, with regression imputation used to calculate values for the weekly means post-withdrawal. Error bars indicate standard error

Mean weekly blood glucose and beta-hydroxybutyrate levels are shown in Fig. 2. The 12-week mean differed with respect to blood glucose (5.86 ± 0.75 mmol/L for the ketogenic diet versus 6.86 ± 1.51 mmol/L for usual diet, *P* < 0.001) and beta-hydroxybutyrate (0.95 ± 0.34 mmol/L versus 0.20 ± 0.06 mmol/L, *P* < 0.001). Of the 21 patients who completed the ketogenic diet, 18 achieved sustained physiological ketosis.

### Primary outcomes

**Fig. 3:**
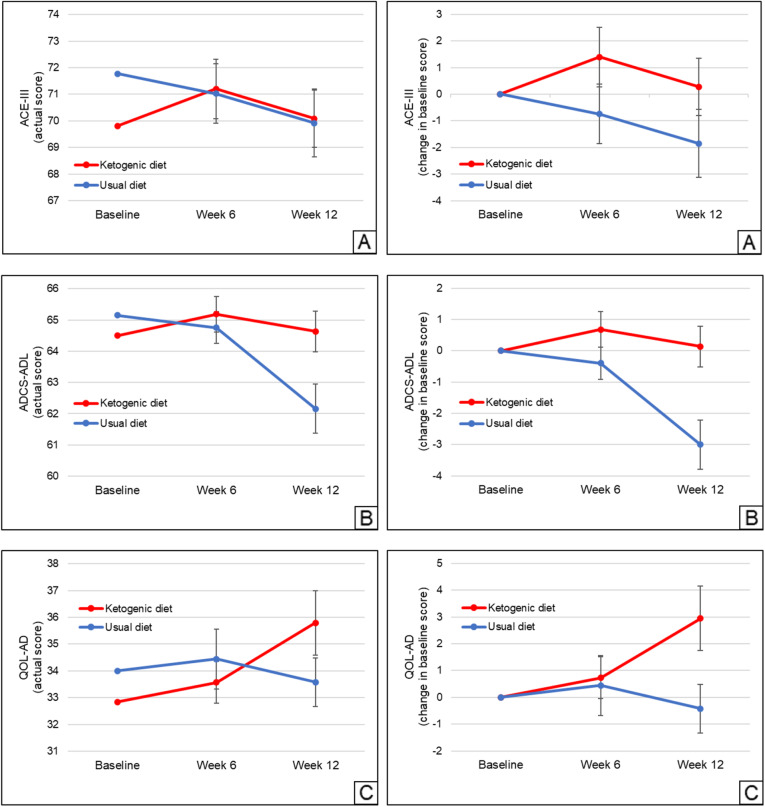
Mean within-individual changes in **a** cognition (ACE-III), **b** daily function (ADCS-ADL), and **c** quality of life (QOL-AD) scores (both the actual scores as well as changes in baseline scores are shown) for all randomized patients (*n* = 26 for ketogenic diet, *n* = 26 for usual diet). Regarding withdrawals, all data up to the point of withdrawal are shown, with regression imputation used to calculate values for the week(s) post-withdrawal. Error bars indicate standard error

Cognition, daily function, and quality of life data are shown in Table 2, and outcome changes are depicted in Fig. 3. The baseline means did not differ between any two comparison groups (*P* > 0.05). Compared with usual diet, patients on the ketogenic diet showed only a modest trend-level change in the ACE-III (+ 2.12 ± 8.70 points, *P* = 0.24) from baseline to week 12, whereas they increased their ADCS-ADL (+ 3.13 ± 5.01 points, *P* = 0.0067) and QOL-AD (+ 3.37 ± 6.86 points, *P* = 0.023) scores.

**Table 2:** Cognition, daily function, and quality of life data for all randomized patients, showing mean baseline scores and changes at weeks 6 and 12 (data shown by treatment period, by treatment sequence, and for all patients)

	First treatment period		Second treatment period		All patients		Treatment effect
	Ketogenic-usual diet (***n*** = 13)	Usual-ketogenic diet (***n*** = 13)	Ketogenic-usual diet (***n*** = 13)	Usual-ketogenic diet (***n*** = 13)	Ketogenic diet (***n*** = 26)	Usual diet (***n*** = 26)	
**Cognition (ACE-III)**							
**Baseline**	69.3 ± 15.2	71.1 ± 15.5	72.5 ± 20.3	70.3 ± 19.3	69.8 ± 17.0	71.8 ± 17.7	**+ 2.12** ± **8.70 (*P*** **= 0.24)**
**Change at week 6**	+ 2.82 ± 7.55	+ 0.46 ± 5.32	−1.96 ± 6.02	− 0.03 ± 2.49	+ 1.39 ± 5.69	− 0.75 ± 5.70	
**Change at week 12**	+ 3.45 ± 4.44	− 1.77 ± 6.04	−1.92 ± 7.26	−2.90 ± 4.57	+ 0.28 ± 5.48	−1.84 ± 6.55	
**Daily function (ADCS-ADL)**							
**Baseline**	66.7 ± 7.7	65.8 ± 8.2	64.5 ± 13.2	62.3 ± 13.0	64.5 ± 10.7	65.2 ± 10.8	**+ 3.13** ± **5.01 (*P*** **= 0.0067)**
**Change at week 6**	+ 1.06 ± 3.53	− 0.85 ± 2.51	+ 0.04 ± 2.70	+ 0.30 ± 2.08	+ 0.68 ± 2.87	−0.40 ± 2.59	
**Change at week 12**	+ 0.22 ± 3.63	−4.46 ± 4.58	− 1.54 ± 2.82	+ 0.04 ± 3.07	+ 0.13 ± 3.30	−3.00 ± 4.00	
**Quality of life (QOL-AD)**							
**Baseline**	33.8 ± 5.6	33.9 ± 5.8	34.1 ± 8.5	31.8 ± 7.3	32.8 ± 6.4	34.0 ± 7.1	**+ 3.37 ± 6.86 (*P*** **= 0.023)**
**Change at week 6**	−0.26 ± 3.39	+ 0.46 ± 3.28	+ 0.43 ± 7.56	+ 1.71 ± 4.37	+ 0.73 ± 3.96	+ 0.45 ± 5.71	
**Change at week 12**	+ 2.86 ± 4.64	−1.15 ± 5.41	+ 0.31 ± 3.68	+ 3.03 ± 7.52	+ 2.95 ± 6.12	−0.42 ± 4.60	

Values are presented as mean ± standard deviation

Regarding withdrawals, all data up to the point of withdrawal are shown, with regression imputation used to calculate values for the week(s) post-withdrawal

Treatment effects and *P* values refer to the difference between the ketogenic and usual diet mean within-individual change at week 12 (for all patients)

*ACE-III* Addenbrookes Cognitive Examination – III, *ADCS-ADL* Alzheimer’s Disease Cooperative Study - Activities of Daily Living, *QOL-AD* Quality of Life in Alzheimer’s Disease

Table 3 shows cognition, daily function, and quality of life data for completers who remained on protocol for both treatment periods and achieved sustained physiological ketosis during the ketogenic diet intervention. The primary outcome results remained similar, and the statistical conclusions unchanged.

**Table 3:** Cognition, function, and quality of life data for completers who remained on protocol for both treatment periods and achieved sustained physiological ketosis (12-week mean beta-hydroxybutyrate level ≥ 0.6 mmol/L) during the ketogenic diet intervention, showing mean baseline scores and changes at weeks 6 and 12 (data shown by treatment period, by treatment sequence, and for all patients)

	First treatment period		Second treatment period		All patients		Treatment effect
	Ketogenic-usual diet (***n*** = 10)	Usual-ketogenic diet (***n*** = 8)	Ketogenic-usual diet (***n*** = 10)	Usual-ketogenic diet (***n*** = 8)	Ketogenic diet (***n*** = 18)	Usual diet (***n*** = 18)	
**Cognition (ACE-III)**							
**Baseline**	73.2 ± 14.3	68.3 ± 17.2	80.1 ± 14.4	66.0 ± 22.1	70.0 ± 18.0	74.8 ± 16.4	**+ 2.56** ± **7.17** **(*P*** **= 0.12)**
**Change at week 6**	+ 4.90 ± 5.24	0.00 ± 4.41	+ 0.40 ± 4.03	0.00 ± 2.45	+ 2.72 ± 4.82	+ 0.22 ± 4.08	
**Change at week 12**	+ 4.30 ± 4.55	−3.00 ± 4.93	− 0.10 ± 3.41	− 2.75 ± 5.95	+ 1.17 ± 6.20	−1.39 ± 4.29	
**Daily function (ADCS-ADL)**							
**Baseline**	69.1 ± 6.5	64.6 ± 8.7	69.6 ± 8.7	60.3 ± 16.2	65.2 ± 12.3	67.4 ± 8.8	**+ 2.95 ± 5.22** **(*P*** **= 0.037)**
**Change at week 6**	+ 1.10 ± 3.90	− 1.00 ± 2.45	− 0.70 ± 2.58	+ 0.63 ± 2.39	+ 0.89 ± 3.23	−0.83 ± 2.46	
**Change at week 12**	+ 0.70 ± 3.59	−4.13 ± 5.46	− 1.00 ± 2.79	+ 0.38± 3.66	+ 0.56 ± 3.52	− 2.39 ± 4.35	
**Quality of life (QOL-AD)**							
**Baseline**	35.4 ± 5.0	32.1 ± 5.8	36.9 ± 7.5	30.3 ± 8.2	33.1 ± 6.9	34.8 ± 7.1	**+ 4.28 ± 7.27(*P*** **= 0.031)**
**Change at week 6**	+ 0.10 ± 3.31	+ 1.00 ± 4.14	− 0.90 ± 7.91	+ 1.75 ± 5.65	+ 0.83 ± 4.44	−0.06 ± 6.41	
**Change at week 12**	+ 3.50 ± 4.38	− 0.63 ± 6.93	− 0.70 ± 3.59	+ 3.75 ± 9.56	+ 3.61 ± 6.91	− 0.67 ± 5.16	

Values are presented as mean ± standard deviation

Treatment effects and *P* values refer to the difference between the ketogenic and usual diet mean within-individual change at week 12 (for all patients)

*ACE-III* Addenbrookes Cognitive Examination – III, *ADCS-ADL* Alzheimer’s Disease Cooperative Study - Activities of Daily Living, *QOL-AD* Quality of Life in Alzheimer’s Disease

### Secondary outcomes

Cardiovascular risk factor data are shown in Table 4. The baseline means did not differ between any two comparison groups (*P* > 0.05). Compared with usual diet, patients on the ketogenic diet decreased in weight, body mass index, and HbA1C, did not alter triglycerides, and increased in HDL, LDL, and total cholesterol from baseline to week 12.

**Table 4:** Cardiovascular risk factor data for all randomized patients, showing mean baseline values and changes at week 12 (data shown by treatment period, by treatment sequence, and for all patients)

	First treatment period		Second treatment period		All patients		Treatment effect
	Ketogenic-usual diet (***n*** = 13)	Usual-ketogenic diet (***n*** = 13)	Ketogenic-usual diet (***n*** = 13)	Usual-ketogenic diet (***n*** = 13)	Ketogenic diet (***n*** = 26)	Usual diet (***n*** = 26)	
**Weight (kg)**							
**Baseline**	86.1 ± 18.4	75.5 ± 11.5	85.2 ± 17.5	75.0 ± 10.8	80.5 ± 15.8	80.4 ± 15.4	**−2.62 ± 3.29 (*P*** **= 0.0017)**
**Change at week 12**	−2.54 ± 3.66	−0.50 ± 1.82	+ 1.29 ± 1.39	− 1.90 ± 1.22	−2.22 ± 2.69	+ 0.40 ± 1.83	
**Body mass index**							
**Baseline**	27.8 ± 5.9	26.6 ± 2.7	27.6 ± 5.5	26.5 ± 2.7	27.1 ± 4.5	27.1 ± 4.3	**− 0.95 ± 1.35 (*P*** **< 0.001)**
**Change at week 12**	− 0.95 ± 1.40	− 0.17 ± 0.63	+ 0.44 ± 0.50	− 0.68 ± 0.42	− 0.81 ± 1.02	+ 0.14 ± 0.64	
**HbA1C (mmol/mol)**							
**Baseline**	34.4 ± 3.2	38.5 ± 6.6	35.7 ± 3.0	40.5 ± 9.3	37.5 ± 7.5	37.1 ± 5.2	**− 2.73 ± 5.17 (*P*** **= 0.0047)**
**Change at week 12**	− 0.88 ± 1.27	+ 0.23 ± 1.59	0.00 ± 2.00	−4.35 ± 7.00	−2.61 ± 5.24	+ 0.12 ± 1.77	
**Triglycerides (mmol/L)**							
**Baseline**	2.45 ± 1.19	2.00 ± 0.89	2.20 ± 0.82	1.84 ± 0.55	2.15 ± 0.96	2.10 ± 0.84	**− 0.32 ± 1.34 (*P*** **= 0.20)**
**Change at week 12**	− 0.43 ± 1.18	− 0.06 ± 0.92	+ 0.20 ± 0.83	− 0.07 ± 0.66	− 0.25 ± 0.96	+ 0.07 ± 0.87	
**HDL (mmol/L)**							
**Baseline**	1.30 ± 0.30	1.49 ± 0.40	1.40 ± 0.34	1.55 ± 0.40	1.43 ± 0.37	1.45 ± 0.37	**+ 0.36 ± 0.32 (*P*** **< 0.001)**
**Change at week 12**	+ 0.27 ± 0.26	− 0.02 ± 0.23	− 0.04 ± 0.13	+ 0.40 ± 0.28	+ 0.33 ± 0.28	−0.03 ± 0.18	
**LDL (mmol/L)**							
**Baseline**	2.68 ± 1.19	2.64 ± 0.88	2.83 ± 1.21	2.65 ± 0.85	2.67 ± 1.01	2.73 ± 1.04	**+ 0.38 ± 1.06 (*P*** **= 0.039)**
**Change at week 12**	+ 0.16 ± 0.69	−0.21 ± 0.55	+ 0.30 ± 0.56	+ 0.69 ± 0.87	+ 0.42 ± 0.81	+ 0.04 ± 0.61	
**Total cholesterol (mmol/L)**							
**Baseline**	5.08 ± 0.96	5.05 ± 0.95	5.22 ± 1.22	5.02 ± 0.92	5.05 ± 0.92	5.13 ± 1.07	**+ 0.62 ± 1.23 (*P*** **= 0.012)**
**Change at week 12**	+ 0.29 ± 0.63	−0.26 ± 0.65	+ 0.35 ± 0.50	+ 1.04 ± 0.94	+ 0.66 ± 0.87	+ 0.04 ± 0.65	

Values are presented as mean ± standard deviation

Regarding withdrawals, regression imputation was used to calculate the value at week 12

Treatment effects and *P* values refer to the difference between the ketogenic and usual diet mean within-individual change at week 12 (for all patients)

*HbA1C* glycosylated haemoglobin, *HDL* high-density lipoprotein, *LDL* low-density lipoprotein

### Adverse effects

Adverse effects are shown in Table 5. The most common adverse effect on both diets was increased irritability. No serious adverse events occurred on the ketogenic diet.

**Table 5:** Adverse effects in weeks 6 and 12 for all randomized patients

Adverse effect	Ketogenic diet (***n*** = 26)		Usual diet (***n*** = 26)	
	Week 6	Week 12	Week 6	Week 12
**Increased irritability**	9 (35%)	5 (19%)	7 (27%)	9 (35%)
**Increased fatigue**	5 (19%)	6 (23%)	2 (8%)	7 (27%)
**Sugar cravings**	5 (19%)	2 (8%)	2 (8%)	6 (23%)
**Insomnia**	2 (8%)	1 (4%)	5 (19%)	5 (19%)
**Muscle cramps**	5 (19%)	3 (12%)	4 (15%)	1 (4%)
**Constipation**	4 (15%)	1 (4%)	2 (8%)	4 (15%)
**Feeling lightheaded**	1 (4%)	4 (15%)	2 (8%)	3 (12%)
**Increased back pain**	2 (8%)	1 (4%)	1 (4%)	3 (12%)
**Excessive hunger**	1 (4%)	2 (8%)	2 (8%)	0
**Excessive thirst**	3 (12%)	1 (4%)	1 (4%)	0
**Nausea**	2 (8%)	0	0	2 (8%)
**Headache**	1 (4%)	0	0	3 (12%)
**Heartburn**	2 (8%)	0	0	2 (8%)
**Diarrhoea**	1 (4%)	1 (4%)	0	0
**Palpitations**	0	1 (4%)	0	1 (4%)
**Urinary calculus**	0	0	0	1 (4%)
**Gout flare-up**	1 (4%)	0	0	0
**Psychotic episode**	0	0	0	1 (4%)
**Total**	44	28	28	48

Regarding withdrawals, all adverse effects experienced up to the point of withdrawal are shown

## Discussion

To our knowledge, this is the first randomized trial to investigate the impact of a ketogenic diet in patients with uniform diagnoses of AD. Our findings suggest that high rates of retention and adherence are achievable in applying a 12-week modified ketogenic diet to AD patients. Compared with a usual diet supplemented with low-fat healthy-eating guidelines, patients on the ketogenic diet improved in daily function and quality of life. Changes in cardiovascular risk factors were mostly favourable and adverse effects were mild.

Among 26 who were randomized, 21 (81%) of the patients completed the ketogenic diet. This is a relatively high proportion compared to previous studies of ketogenic diets in AD [13, 14]. Moreover, half the patients and trial partners stated that they intended to continue the ketogenic diet after the trial. This proportion is also high given that ketogenic diets are often considered unpalatable [25]. We partially attribute these results to our AD-tailored ketogenic diet, which utilized affordable and simple recipes aimed at satisfying a typical AD preference for sweet foods [26]. The high retention rate may also relate to our exclusion of medium-chain triglyceride oils, which produce adverse gastrointestinal effects [13], and our educational programme. Importantly, only one withdrawal was attributed to ill effects of the ketogenic diet, even though the trial partner was enthusiastic. The fact that the remaining four non-completers declared no interest in altering their usual diet suggests that patient motivation is essential when commencing a ketogenic diet in AD.

Patients on the ketogenic diet achieved a 12-week mean blood beta-hydroxybutyrate level of 0.95 ± 0.34 mmol/L, which is consistent with a state of physiological ketosis and corresponds to ketones supplying 12–15% of brain energy requirements [5]. This level compares favourably with the average of 0.3–0.6 mmol/L achieved in previous studies of ketosis in AD [13, 27, 28] and indicates that most of our patients adhered to the diet, with 18 patients achieving sustained physiological ketosis. We attribute the high adherence rate to our AD-tailored ketogenic diet and to the use of blood glucose and ketone monitors. Blood monitors relay the level of beta-hydroxybutyrate, which is the principal ketone contributing to brain energy metabolism and therefore the main variable of interest when evaluating adherence to a ketogenic diet. They are easy to operate and can be used daily, allowing difficulties to be swiftly recognized and corrected. In contrast, food records reflect adherence to a prescribed diet but do not relay the actual level of ketosis achieved. They can also be burdensome for cognitively impaired individuals [29].

Although cognition is an important outcome measure in AD, daily function and quality of life are factors of great importance to people living with dementia as they focus on what a person can do, or how they feel [30]. We therefore measured changes in these variables (as well as cognitive performance) as primary outcomes.

Compared with usual diet, patients on the ketogenic diet showed only a non-significant trend towards improved cognition, increasing by 2.12 ± 8.70 points on the ACE-III. An observed reversal of trends in ACE-III during weeks 7 to 12 may have been attributable to a national COVID-19 lockdown that occurred during weeks 7 to 11 of the second treatment period; common increases in anxiety related to the lockdown may have had adverse consequences in ketogenic diet patients who were not yet fully adapted to the additional stress of a new dietary lifestyle. Alternatively, the trend reversal may be explained by the higher prevalence of apolipoprotein E4 carriers among ketogenic diet patients in the second treatment period; ketone energy metabolism may be less beneficial for apolipoprotein E4 carriers [27, 31], although not all studies have shown this [14, 32].

Compared with usual diet, patients on the ketogenic diet improved in daily function. Given that a 2-point change on the ADCS-ADL is considered clinically meaningful [22], the observed increase of 3.13 points implies that the ketogenic diet imparted a meaningful benefit in our patients to a degree that rarely occurs with medications [33]. Since an ongoing decline in daily function is a core feature of AD [2], this finding may be potentially important. By contrast, the decline in function observed in patients on low-fat healthy-eating guidelines may raise questions as to whether this dietary advice should be provided in AD.

Compared with usual diet, patients on the ketogenic diet also improved in quality of life. Given that a 3-point change on the QOL-AD is considered clinically meaningful [23], the observed increase of 3.37 points again implies that the ketogenic diet may offer a substantial benefit. By comparison, cholinesterase inhibitors show inconsistent effects on quality of life [34].

Whether ketogenic diets offer benefits on cardiovascular risk factors remains controversial [35]. The changes observed in this trial were mostly favourable. Compared with usual diet, patients on the ketogenic diet lost 2.62 ± 3.29 kg. Weight loss has been associated with increased mortality in AD [36], but that observation may relate to appetite changes resulting in cachexia in later-stage disease. By contrast, our patients were generally overweight (and pleased with their weight loss). Moreover, patients on the ketogenic diet decreased their HbA1C, did not alter triglycerides, and increased their HDL. There were modest increases in LDL and total cholesterol, but the impact of these changes on cardiovascular health remains debated [37].

Adverse effects of the ketogenic diet were mild. The most common adverse effect on both diets was increased irritability, which is unsurprising given that agitation is frequently observed in AD [2]. Importantly, patients on the ketogenic diet experienced nearly twice as many adverse effects in week 6 as in week 12, which suggests that patients may become less distressed as they acclimate to the new diet.

Our use of a randomized crossover design may raise potential concerns about carryover and period effects [24]. Given that cognitive changes, if any, induced by a ketogenic diet in AD typically return to baseline by 1 month [13], we considered a 10-week washout period sufficient to avoid effects of the diets continuing from the first treatment period. We also compared the baseline means for all comparison groups and found that the 10-week interval was short enough to avoid outcome changes related to natural AD progression.

Several trial strengths may be mentioned. First, all our patients had uniform NINCDS-ADRDA diagnoses of probable AD (as opposed to mild cognitive impairment or possible AD). Second, we utilized a single-phase design, strictly enforced timing, diet-blinded assessors, and validated assessment tools to minimize assessment bias. Third, our AD-tailored diet plan contained a variety of affordable and simple recipe options, which appeared to have promoted a high retention rate and sustained physiological ketosis. Fourth, along with cognition, our primary outcomes included changes in daily function and quality of life, which are the factors of particular importance to AD patients and their families.

### Limitations

Among its limitations, the trial’s sample size and duration were small; a larger sample or longer diet intervention could have provided additional statistical power. Thus, our findings should be considered as preliminary. Second, even with rigorous application of the NINCDS-ADRDA criteria, the clinical diagnostic accuracy for distinguishing autopsy-confirmed AD from other dementia types is limited [38], and we might have enrolled some non-AD patients. We attempted to mitigate this concern by ensuring every patient had recent brain imaging, which may increase diagnostic accuracy in AD [38]. Third, although assessors were blinded to diet, patients and trial partners could not be blinded to their food, and their awareness may have resulted in different expectations of benefit (or harm) for one diet compared to the other. We tried to mitigate this possibility by presenting both diet approaches as potentially healthy. Fourth, patients on the ketogenic diet experienced a modest degree of weight loss compared to usual diet, and this in itself may have affected other clinical outcomes. We tried to mitigate this possibility by consistently encouraging all patients to eat until satiation. Fifth, patients were repeatedly assessed using the same cognitive scale, which may have resulted in a learning effect on subsequent testing. We tried to mitigate this possibility by utilizing different versions of the ACE-III throughout the trial (moreover, even if a learning effect did occur, it would logically have affected both diets similarly).

## Conclusions

In conclusion, our findings suggest that high rates of retention and adherence are achievable in applying a 12-week modified ketogenic diet to AD patients. Compared with a usual diet supplemented with low-fat healthy-eating guidelines, patients on the ketogenic diet improved in daily function and quality of life, two factors of great importance to people living with dementia. Changes in cardiovascular risk factors were mostly favourable and adverse effects were mild. Ketogenic diets may hold promise as viable and effective treatment strategies in AD, but larger and longer studies are needed before this can be stated with confidence.

## Supplementary Information

**Additional file 1.**

## Data Availability

The data that support the findings of this trial are freely available from the corresponding author, upon reasonable request.
