# Supplementary material for: Randomized crossover trial of a modified ketogenic diet in Alzheimer’s disease
Source: Alzheimers Res Ther. 2021 Feb 23;13:51. doi: 10.1186/s13195-021-00783-x (PMC7901512; doi:10.1186/s13195-021-00783-x)
Supplement: Supplementary file 1 — Additional file 1. [file 13195_2021_783_MOESM1_ESM.zip › Healthy Eating Recipes - Alzheimers Dietary Study.pdf]

Healthy Eating Recipes

Healthy Diet Recipes

The Alzheimer’s Dietary Study

2019-2020

Table Of Contents

The Commitment….3

Your Nightly Record (Write Down Your Numbers)….4

Optional Recipes….8

New Zealand Nutrition Guidelines….50

The Commitment

"Let food be thy medicine, and medicine be thy food."
- Hippocrates

The **guidelines** for this plan are simple:

(1) **The recommended recipes are optional** - We want you to keep eating your usual diet over the next 3 months, but you may add in these optional recipes as you like. The recipes are designed to be low-fat, high-fibre in accordance with the New Zealand Nutrition Guidelines, which are attached at the end for you.

(2) **Record your bedtime glucose and ketones** - Check your blood glucose and ketones every night at bedtime, then write the numbers in the boxes on pages 4-7. Try to keep the ketones **under 0.3 mmol/L**, at all times.

(3) **If you don't feel like cooking, it's ok** - Sometimes, it's nice to have a break from cooking and eat at a restaurant or social function! This is ok; eat out as you normally would.

You may email me at Matthew.Phillips@waikatodhb.health.nz any time. You may also email Deborah at nutritioninalzheimers@gmail.com any time.

Sincerely,

Matt, Deborah, Stacey, Grace…and the rest of the Alzheimer's Dietary Study team.

Your Nightly Record (Write Down Your Numbers, Every Night At Bedtime)

Week 1

February 17, 2020	Bedtime Glucose		Bedtime Ketones	
February 18, 2020	Bedtime Glucose		Bedtime Ketones	
February 19, 2020	Bedtime Glucose		Bedtime Ketones	
February 20, 2020	Bedtime Glucose		Bedtime Ketones	
February 21, 2020	Bedtime Glucose		Bedtime Ketones	
February 22, 2020	Bedtime Glucose		Bedtime Ketones	
February 23, 2020	Bedtime Glucose		Bedtime Ketones	

Week 2

February 24, 2020	Bedtime Glucose		Bedtime Ketones	
February 25, 2020	Bedtime Glucose		Bedtime Ketones	
February 26, 2020	Bedtime Glucose		Bedtime Ketones	
February 27, 2020	Bedtime Glucose		Bedtime Ketones	
February 28, 2020	Bedtime Glucose		Bedtime Ketones	
February 29, 2020	Bedtime Glucose		Bedtime Ketones	
March 1, 2020	Bedtime Glucose		Bedtime Ketones	

Week 3

March 2, 2020	Bedtime Glucose		Bedtime Ketones	
March 3, 2020	Bedtime Glucose		Bedtime Ketones	
March 4, 2020	Bedtime Glucose		Bedtime Ketones	
March 5, 2020	Bedtime Glucose		Bedtime Ketones	
March 6, 2020	Bedtime Glucose		Bedtime Ketones	
March 7, 2020	Bedtime Glucose		Bedtime Ketones	
March 8, 2020	Bedtime Glucose		Bedtime Ketones	

Week 4

March 9, 2020	Bedtime Glucose		Bedtime Ketones	
March 10, 2020	Bedtime Glucose		Bedtime Ketones	
March 11, 2020	Bedtime Glucose		Bedtime Ketones	
March 12, 2020	Bedtime Glucose		Bedtime Ketones	
March 13, 2020	Bedtime Glucose		Bedtime Ketones	
March 14, 2020	Bedtime Glucose		Bedtime Ketones	
March 15, 2020	Bedtime Glucose		Bedtime Ketones	

Week 5

March 16, 2020	Bedtime Glucose		Bedtime Ketones	
March 17, 2020	Bedtime Glucose		Bedtime Ketones	
March 18, 2020	Bedtime Glucose		Bedtime Ketones	
March 19, 2020	Bedtime Glucose		Bedtime Ketones	
March 20, 2020	Bedtime Glucose		Bedtime Ketones	
March 21, 2020	Bedtime Glucose		Bedtime Ketones	
March 22, 2020	Bedtime Glucose		Bedtime Ketones	

Week 6

March 23, 2020	Bedtime Glucose		Bedtime Ketones	
March 24, 2020	Bedtime Glucose		Bedtime Ketones	
March 25, 2020	Bedtime Glucose		Bedtime Ketones	
March 26, 2020	Bedtime Glucose		Bedtime Ketones	
March 27, 2020	Bedtime Glucose		Bedtime Ketones	
March 28, 2020	Bedtime Glucose		Bedtime Ketones	
March 29, 2020	Bedtime Glucose		Bedtime Ketones	

Week 7

March 30, 2020	Bedtime Glucose		Bedtime Ketones	
March 31, 2020	Bedtime Glucose		Bedtime Ketones	
April 1, 2020	Bedtime Glucose		Bedtime Ketones	
April 2, 2020	Bedtime Glucose		Bedtime Ketones	
April 3, 2020	Bedtime Glucose		Bedtime Ketones	
April 4, 2020	Bedtime Glucose		Bedtime Ketones	
April 5, 2020	Bedtime Glucose		Bedtime Ketones	

Week 8

April 6, 2020	Bedtime Glucose		Bedtime Ketones	
April 7, 2020	Bedtime Glucose		Bedtime Ketones	
April 8, 2020	Bedtime Glucose		Bedtime Ketones	
April 9, 2020	Bedtime Glucose		Bedtime Ketones	
April 10, 2020	Bedtime Glucose		Bedtime Ketones	
April 11, 2020	Bedtime Glucose		Bedtime Ketones	
April 12, 2020	Bedtime Glucose		Bedtime Ketones	

Week 9

April 13, 2020	Bedtime Glucose		Bedtime Ketones	
April 14, 2020	Bedtime Glucose		Bedtime Ketones	
April 15, 2020	Bedtime Glucose		Bedtime Ketones	
April 16, 2020	Bedtime Glucose		Bedtime Ketones	
April 17, 2020	Bedtime Glucose		Bedtime Ketones	
April 18, 2020	Bedtime Glucose		Bedtime Ketones	
April 19, 2020	Bedtime Glucose		Bedtime Ketones	

Week 10

April 20, 2020	Bedtime Glucose		Bedtime Ketones	
April 21, 2020	Bedtime Glucose		Bedtime Ketones	
April 22, 2020	Bedtime Glucose		Bedtime Ketones	
April 23, 2020	Bedtime Glucose		Bedtime Ketones	
April 24, 2020	Bedtime Glucose		Bedtime Ketones	
April 25, 2020	Bedtime Glucose		Bedtime Ketones	
April 26, 2020	Bedtime Glucose		Bedtime Ketones	

Week 11

April 27, 2020	Bedtime Glucose		Bedtime Ketones	
April 28, 2020	Bedtime Glucose		Bedtime Ketones	
April 29, 2020	Bedtime Glucose		Bedtime Ketones	
April 30, 2020	Bedtime Glucose		Bedtime Ketones	
May 1, 2020	Bedtime Glucose		Bedtime Ketones	
May 2, 2020	Bedtime Glucose		Bedtime Ketones	
May 3, 2020	Bedtime Glucose		Bedtime Ketones	

Week 12

May 4, 2020	Bedtime Glucose		Bedtime Ketones	
May 5, 2020	Bedtime Glucose		Bedtime Ketones	
May 6, 2020	Bedtime Glucose		Bedtime Ketones	
May 7, 2020	Bedtime Glucose		Bedtime Ketones	
May 8, 2020	Bedtime Glucose		Bedtime Ketones	

BREAKFAST..9
French Toast & Honey..10
Bran Muffins..11
Green Smoothie..12
Raspberry-Peach-Mango Granola Smoothie Bowl..13
Strawberry Mango Smoothie..14
Granola Fruit Cereal..15
Poached Eggs & Split..16
Wake-Up Smoothie..17
Fried Eggs..18
Scrambled Eggs On Toast..19

LUNCH..20
Salmon Salad..21
Hamburger..22
Raw Energy Salad..23
Minestrone Soup..24
Carrot & Cashew Salad..25
Chicken & Vegetable Soup..26
Mini Pizzas..27

DINNER..28
Beef Stroganoff..29
Baked Chicken..30
Smokey Hotpot..31
Bean Burritos..32
Vermicelli & Homestyle Tomato Sauce..33
Tuna Casserole..34
Roast Vegetables..35
Homestyle Vegetable Lasagne..36

SIDE DISHES..37
Spanish Rice..38
Indian Rice..39
Stir-Fried Vegetables..40
Steamed Broccoli..41
Low Fat Vegetable Medley..42

DESSERT..43
Berry Hot Drink..44
Berry Fruit Salad..45
Banana Bread..46
Lemon Lime Fruit Salad..47
Apple Crisp..48
Sunshine Salad..49

Breakfast

French Toast & Honey

This recipe makes one serving (one serving = two slices bread).

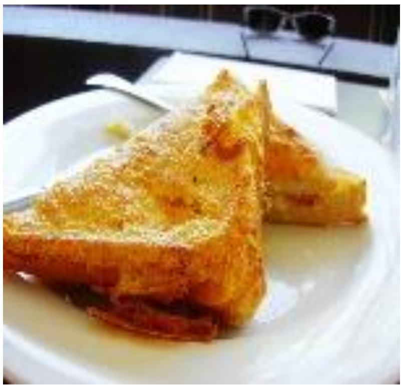

Ingredients:

1 egg
3 tablespoons Value trim low fat milk
1 teaspoon pure vanilla extract
2 teaspoons Anchor lite dairy blend
2 slices wholemeal bread
2 tablespoons honey (or maple syrup)
1 teaspoon cinnamon

Preparation:

(1) Break the egg into a mixing bowl. Beat the egg slightly with a whisk or fork. Add the milk and vanilla and mix it all together to create the egg-and-milk mixture.
(2) Melt the dairy blend in a pan over medium heat.
(3) Dip each side of the bread quickly into the egg-and-milk mixture until it is well coated, but not entirely soaked with the mixture.
(4) Put the bread in the hot pan and cook it over medium heat until it is golden brown on one side. Turn and cook the other side until it is also golden brown. Repeat with the other slice of bread (if you have a big enough pan, you can cook both slices together).
(5) Pour the honey over top and sprinkle with cinnamon - serve hot!

Nutrition Information:

Calories: 486.4
Fat: 11.3 g
Protein 15.1 g
Net Carbs: 78.5 g
Fibre: 5.2 g

Bran Muffins

This recipe makes 12 muffins (one serving = two muffins).

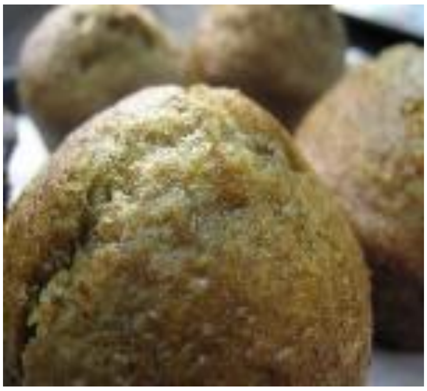

Ingredients:

½ cup wholemeal flour
1 cup pure plain flour
1½ cups wheat germ
1 teaspoon baking soda
½ teaspoon salt
½ cup raisins
1 cup Value trim low fat milk
¼ cup molasses
1 tablespoon honey (or maple syrup)
¼ cup brown sugar
1 egg
2 teaspoons pure vanilla extract
1 tablespoon (½ lemon) juice
2 tablespoons canola oil

Preparation:

(1) Preheat the oven to 200°C.
(2) In a large bowl, mix the flours, wheat germ, baking soda, salt, and raisins. Set aside.
(3) In another bowl, mix the milk with the molasses, honey, brown sugar, egg, vanilla, lemon juice, and oil.
(4) Add the wet ingredients to the dry ingredients in the large bowl. Stir everything just enough to moisten everything - don't overmix.
(5) Fill 12 muffin cups about two thirds full and bake for 12-14 minutes. Cool and serve.

Nutrition Information:

Calories: 2361.5 (196.8 per muffin)
Fat: 52.9 g (4.4 g per muffin)
Protein 80.2 g (6.7 g per muffin)
Net Carbs: 377.7 g (31.5 g per muffin)
Fibre: 36.2 g (3 g per muffin)

Green Smoothie

This recipe makes one serving.

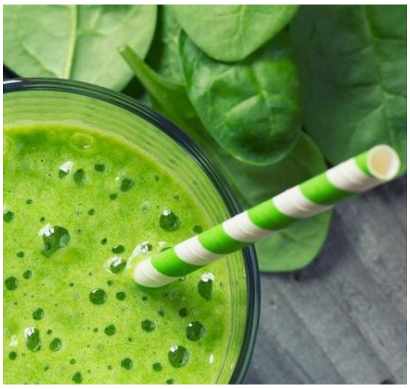

Ingredients:

1 cup frozen mango chunks
60 g spinach (or kale) leaves
½ cup water
½ banana
2 tablespoons (1 lemon) juice

Preparation:

(1) Put the mangos, spinach, water, and banana into an electric blender. Squeeze all the lemon juice into the blender.
(2) Blend together and serve.

Nutrition Information:

Calories: 199.5
Fat: 0.3 g
Protein: 1.9 g
Net Carbs: 47.5 g
Fibre: 5.4 g

Raspberry-Peach-Mango Granola Smoothie Bowl

This recipe makes one serving.

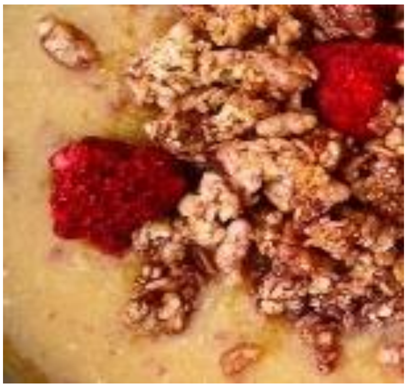

Ingredients:

1 cup frozen mango chunks
½ cup Yoplait 97% fat free yoghurt
¼ cup Value trim low fat milk
1 teaspoon pure vanilla extract
¼ cup Hubbard's granola
½ peach (or persimmon)
½ cup raspberries (or blueberries)

Preparation:

(1) Combine the mangos, yoghurt, milk, and vanilla in an electric blender. Pulse until you have created a smoothie. Pour the smoothie into a bowl.
(2) Mix the granola into the smoothie.
(3) Slice up the peach and rinse the berries; place on top. Enjoy!

Nutrition Information:

Calories: 430
Fat: 9.1 g
Protein: 11.2 g
Net Carbs: 68.2 g
Fibre: 9.6 g

Strawberry Mango Smoothie

This recipe makes one serving.

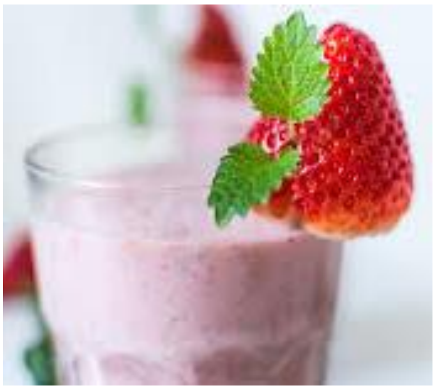

Ingredients:

1 cup strawberries
1 cup frozen mango chunks
½ cup Yoplait 97% fat free yoghurt
6 ice cubes (optional)

Preparation:

(1) Combine the strawberries, mangos, and yoghurt in an electric blender. Pulse until you have created a smoothie.
(2) If you want, add in the ice cubes and pulse until smooth. Serve.

Nutrition Information:

Calories: 276.5
Fat: 4.1 g
Protein: 6 g
Net Carbs: 51.8 g
Fibre: 5.6 g

Granola Fruit Cereal

This recipe makes one serving.

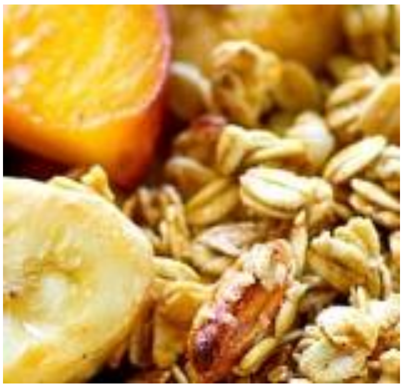

Ingredients:

½ cup Hubbard's granola
1 banana
½ peach (or persimmon)
¾ cup Value trim low fat milk

Preparation:

(1) Place the granola into a bowl.
(2) Chop up the banana and peach, and add to the granola.
(3) Pour milk over top and enjoy!

Nutrition Information:

Calories: 450.3
Fat: 10.4 g
Protein 14.2 g
Net Carbs: 68.8
Fibre: 8.1

Poached Eggs & Split

This recipe makes one serving (one serving = two eggs).

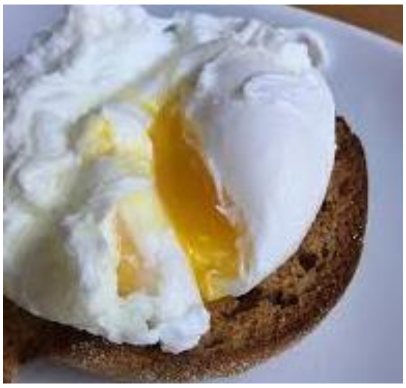

Ingredients:

1 teaspoon white vinegar
2 eggs
1 Quality Bakers whole grain muffin split
Salt and pepper to taste

Preparation:

(1) Simmer a pot of water and add the vinegar. Crack one egg into a cup, create a gentle whirlpool, then slowly tip the egg into the water. Cook 2-4 minutes, depending how hard you want the egg. Remove with a slotted spoon. Repeat with one more egg.
(2) Toast the muffin split halves and place one egg on each half. Season with the salt and pepper, and serve.

Nutrition Information:

Calories: 299
Fat: 11.3 g
Protein 19 g
Net Carbs: 24.1 g
Fibre: 2.5 g

Wake-Up Smoothie

This recipe makes one serving.

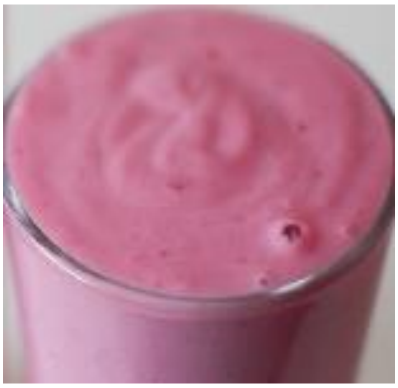

Ingredients:

½ cup orange juice
200 g (½ can) apricots in juice
1 banana
½ cup raspberries (or blueberries)
¼ cup Yoplait 97% fat free yoghurt

Preparation:

(1) Combine all ingredients in an electric blender and pulse until smooth. Serve!

Nutrition Information:

Calories: 430.3
Fat: 3 g
Protein: 6.9 g
Net Carbs: 88.1 g
Fibre: 13.1 g

Fried Eggs

This recipe makes one serving.

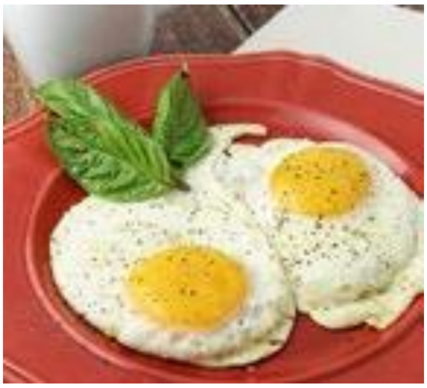

Ingredients:

2 teaspoons canola oil
2 eggs
Salt and pepper to taste

Preparation:

(1) Heat the oil in a pan on medium heat.
(2) Once hot, crack the eggs into the oil in the pan and cook the eggs until the whites are solid. There should be no more raw egg whites on top of the egg.
(3) Add salt and pepper and serve.

Nutrition Information:

Calories: 239
Fat: 19.6 g
Protein: 14 g
Net Carbs: 0.8 g
Fibre: 0 g

Scrambled Eggs On Toast

This recipe makes one serving (one serving = two eggs).

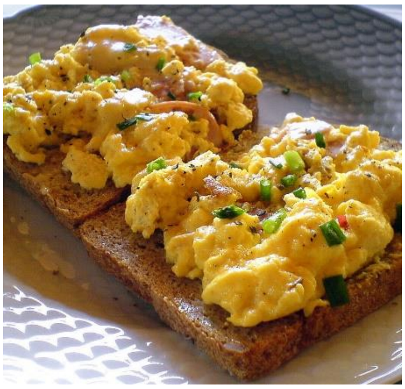

Ingredients:

2 eggs
1 tablespoon Value trim low fat milk
Salt and pepper to taste
2 teaspoons Anchor lite dairy blend
1 spring onion stalk
2 slices wholemeal bread

Preparation:

(1) Break the eggs into a small bowl.
(2) Add milk, salt, and pepper, then beat with a fork to blend the whites and yolks.
(3) Melt the dairy blend in a pan over medium heat. Chop and add the spring onion; let it cook for 1-2 minutes.
(4) Pour the egg mixture into the pan and cook over medium heat, very gently pulling the eggs across the pan to form large soft curds.
(5) Toast the bread, place the eggs on the toast, and serve.

Nutrition Information:

Calories: 419.8
Fat: 16.6 g
Protein 20.8 g
Net Carbs: 41.8 g
Fibre: 4 g

Lunch

Salmon Salad

This recipe makes one serving.

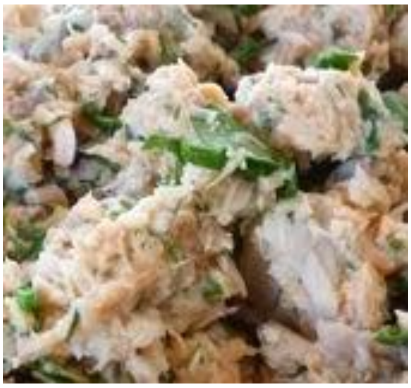

Ingredients:

85 g (1 small can) Sealord canned salmon
1 spring onion stalk
½ short cucumber (or ½ courgette)
2 tablespoons lite mayonnaise
½ teaspoon mustard
1 tablespoon (½ lemon) juice
Salt and pepper to taste

Preparation:

(1) Mash the salmon in a bowl.
(2) Chop up and add the spring onion and cucumber.
(3) Mix the mayonnaise, mustard, and lemon juice in a separate bowl (or a sealed container of you are going to work).
(4) When ready to eat, mix the dressing into the salmon and season with salt and pepper. Serve.

Nutrition Information:

Calories: 209
Fat: 13.5 g
Protein: 14.9 g
Net Carbs: 7.7 g
Fibre: 0.8 g

Hamburger

This recipe makes one serving (one serving = one hamburger).

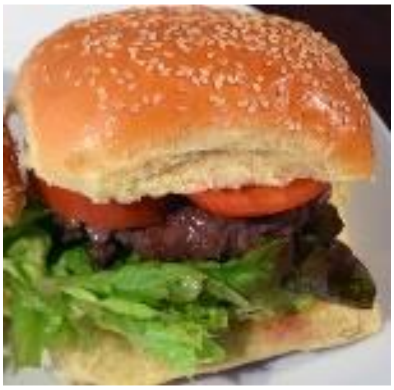

Ingredients:

1 teaspoon canola oil
115 g prime (5% fat) beef mince (or steak)
Salt and pepper to taste
1 Quality Bakers nature's fresh burger bun
½ tomato
¼ small brown onion
2 lettuce leaves (optional)
1 tablespoon ketchup (or lite mayonnaise)

Preparation:

(1) Heat the oil in a pan over medium heat.
(2) While it heats up, place the beef mince on a plate and shape it into an orb 3-4 cm high.
(3) When you're ready to transfer the meat to the pan, turn the heat to high and put the orb into the pan. Very quickly, using a stiff metal spatula, press down on the orb to form a thin patty only 1 cm thick and 10-12 cm in diameter; it must be 1 cm think or the patty may not cook through to the middle. Season with salt and pepper.
(4) Cook the patty without moving it until it has achieved a deep, burnished crust; this takes roughly 90 seconds.
(5) Slide your spatula under the patty, flip it, and cook the patty for another 60 seconds.
(6) Check the middle of the burger - if the inside is still raw, reduce the heat to medium and place it on the pan another 30 seconds, flip it, cook the other side another 30 seconds. Check it again, and repeat until the inside is cooked.
(6) Remove to the bun, slice up the tomato and onion and add them to the patty followed by the lettuce and ketchup, and serve.

Nutrition Information:

Calories: 519.1
Fat: 13.9 g
Protein: 39.5 g
Net Carbs: 51.8 g
Fibre: 3.9 g

Raw Energy Salad

This recipe makes two servings.

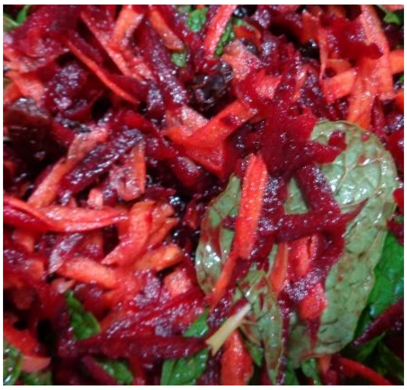

Ingredients:

1 beetroot
1 carrot
½ cup mint leaves (chopped)
¼ cup raisins
¼ teaspoon salt
1 tablespoon Balsamic vinegar
1 tablespoon honey
¼ cup orange juice

Preparation:

(1) Mix together the beetroots, carrots, mint leaves, raisins, and salt in a bowl.
(2) To prepare the dressing, add the vinegar, honey, and orange juice to a sealed container and shake until they are well combined, particularly the honey.
(3) When ready to eat, pour over the dressing and serve.

Nutrition Information:

Calories: 268.5 (134.3 per serving)
Fat: 0.6 g (0.3 g per serving)
Protein: 3.7 g (1.9 g per serving)
Net Carbs: 62.3 g (31.2 g per serving)
Fibre: 5.9 g (3 g per serving)

Minestrone Soup

This recipe makes two servings.

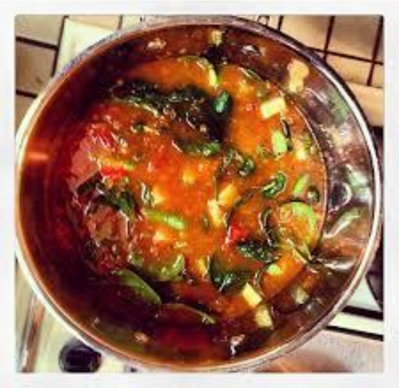

Ingredients:

3 cups reduced salt chicken stock
2 cups cabbage (chopped)
3 carrots
1 teaspoon oregano
400 g canned kidney beans
1 tomato
1 clove garlic
Salt and pepper to taste
2 tablespoons fresh basil (chopped)

Preparation:

(1) Add the chicken stock to a large pot and bring to a boil over high heat. Meanwhile, chop up the cabbage and dice the carrots.
(2) Turn the heat down to low-medium, then add the cabbage, carrots, and oregano to the pot.
(3) Cover the pot and simmer until the vegetables are tender, about 15-20 minutes.
(4) When the 15-20 minutes are up, drain and rinse the kidney beans, then add the kidney beans, sliced tomatoes, minced garlic, salt, and pepper to the pot.
(5) Turn the heat up to medium, cover the pot and cook another 6-8 minutes. Top with the basil and serve.

Nutrition Information:

Calories: 549 (274.5 per serving)
Fat: 3.3 g (1.7 g per serving)
Protein 38.7 g (19.4 g per serving)
Net Carbs: 68.8 g (34.4 g per serving)
Fibre: 28.7 g (14.4 g per serving)

Carrot & Cashew Salad

This recipe makes two servings.

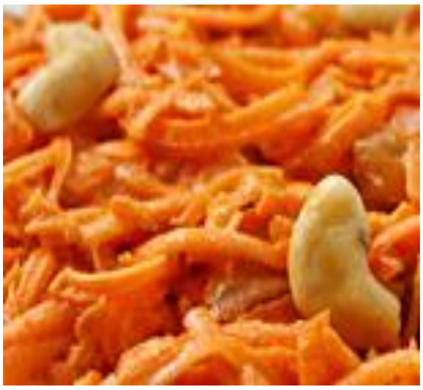

Ingredients:

2 carrots
¼ cup cashews
1 teaspoon ground coriander
2 tablespoons fresh coriander (chopped)
¼ cup (2 lemons) juice
1 tablespoon canola oil
½ clove garlic
½ teaspoon cumin
¼ teaspoon salt

Preparation:

(1) Grate the carrots.
(2) Mix everything together in a bowl (or a sealed container if you are going to work) and serve.

Nutrition Information:

Calories: 359 (179.5 per serving)
Fat: 28.1 g (14.1 g per serving)
Protein: 7.1 g (3.6 g per serving)
Net Carbs: 18.7 g (9.4 g per serving)
Fibre: 5.2 g (2.6 g per serving)

Chicken & Vegetable Soup

This recipe makes four servings.

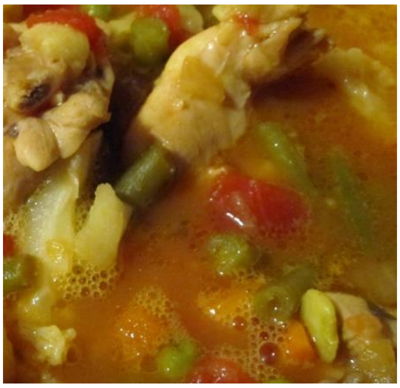

Ingredients:

4 chicken drumsticks (skin off)
2 cloves garlic
1 teaspoon salt
1 teaspoon black pepper
2 teaspoons thyme
6 teaspoons parsley
4 celery stalks (or 2 courgettes)
4 potatoes
2 cups frozen peas
¼ cup brown rice (uncooked)

Preparation:

(1) Measure 8 cups of water into a large pot and place over high heat.
(2) Add the drumsticks, minced garlic, salt, pepper, thyme, and parsley to the pot. Bring to a boil - once boiling, turn the stove down to low heat and simmer for 50-60 minutes.
(3) Chop up the celery and cube the potatoes, then add the celery, potatoes, peas, and rice to the pot. Let the pot simmer another 30-50 minutes, until the rice and potatoes are soft.
(4) When cooked, remove the meat from the drumsticks and put the meat back into the soup. Discard the bones. Ready to serve.

Nutrition Information:

Calories: 1360 (453.3 per serving)
Fat: 11.5 (3.8 g per serving)
Protein: 88.4 (29.5 g per serving)
Net Carbs: 193.3 (64.4 g per serving)
Fibre: 34.4 (11.5 g per serving)

Mini Pizzas

This recipe makes one serving (one serving = two mini pizzas).

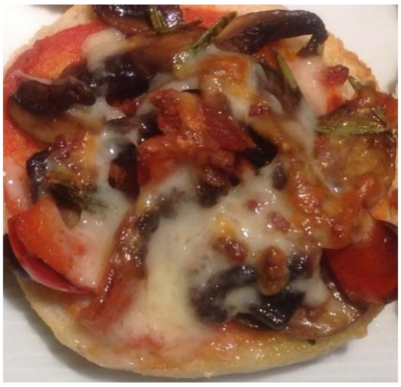

Ingredients:

1 serving Homestyle Tomato Sauce
2 Quality Bakers whole grain muffin splits
Basil and oregano to taste
Salt and pepper to taste
2 spring onion stalks
½ green capsicum
4 small mushrooms
60 g mozzarella cheese

Preparation:

(1) Preheat the oven to 180°C.
(2) Make the Homestyle Tomato Sauce now, without the vermicelli. Set aside.
(3) Slice the muffin splits in half and place the four halves on a baking tray, then spread one quarter of the sauce on each muffin split half. Sprinkle basil, oregano, salt, and pepper over the top.
(4) Finely chop the spring onion, green capsicum, and mushrooms. Layer them on top of each muffin split half. Sprinkle grated cheese over the top.
(5) Bake until hot and bubbly, about 10-15 minutes. Serve.

Nutrition Information:

Calories: 642
Fat: 24.2 g
Protein: 28.3 g
Net Carbs: 65.1 g
Fibre: 8.4 g

Dinner

Beef Stroganoff

This recipe makes three servings.

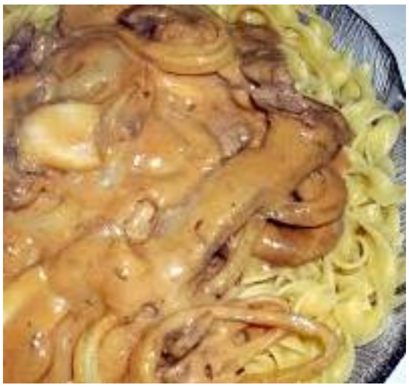

Ingredients:

1 cup penne (uncooked)
200 g prime (5% fat) beef mince
½ small brown onion
¾ cup frozen peas
150 g cream of mushroom soup
¼ cup Value trim low fat milk
½ cup light sour cream
¼ teaspoon salt
¼ teaspoon black pepper
Dash of garlic salt
2 teaspoons Worcester sauce
1 slice wholemeal bread
1 tablespoon Anchor lite dairy blend

Preparation:

(1) Preheat the oven to 180°C.
(2) Measure 4 cups of water into a pot on high heat and bring to a boil. Once it boils, cook the penne for 7-9 minutes, then drain and set aside.
(3) Meanwhile, saute the chopped onion and brown the beef in a pan over medium heat (cook in one teaspoon of canola oil if you like).
(4) Mix the penne, peas, mushroom soup, milk, sour cream, salt, pepper, garlic salt, and Worcester sauce into the beef mixture, then transfer everything to a casserole dish.
(5) To make the breadcrumbs, tear one slice of bread into small pieces and process it in an electric blender until small coarse crumbs form. Mix the breadcrumbs with the dairy blend and top the casserole with this mixture.
(6) Bake for 30 minutes and serve!

Nutrition Information:

Calories: 1356.2 (452.1 per serving)
Fat: 47.4 g (15.8 g per serving)
Protein: 89.9 g (30 g per serving)
Net Carbs: 130.1 g (43.4 g per serving)
Fibre: 13.3 g (4.4 g per serving)

Baked Chicken

This recipe makes two servings.

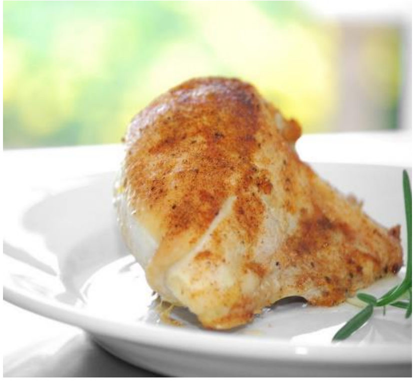

Ingredients:

1 teaspoon salt
240 g deboned chicken breasts (skin off)
1 tablespoon canola oil
½ teaspoon salt
½ teaspoon black pepper
½ teaspoon garlic salt
½ teaspoon paprika

Preparation:

(1) Fill a big bowl with warm (not hot) water and stir in the salt. Brine the chicken breasts in the water for 15-20 minutes.
(2) Remove the chicken breasts from the brine, rinse with cold water, and pat dry with paper towel.
(3) Preheat the oven to 230°C.
(4) Put the canola oil into a small bowl and melt it in the microwave. Coat both sides of the chicken breasts with the oil and place them in a baking dish.
(5) Mix together the salt, pepper, garlic salt, and paprika in a separate bowl until they are combined, then sprinkle the mixture over both sides of the chicken breasts.
(6) Bake for 15-18 minutes, or until the chicken is cooked through and no longer pink. Rest the chicken breasts for 5-10 minutes then serve.

Nutrition Information:

Calories: 384 (192 per serving)
Fat: 16.9 g (8.5 g per serving)
Protein: 54.5 g (27.3 g per serving)
Net Carbs: 0 g (0 g per serving)
Fibre: 0 g (0 g per serving)

Smokey Hotpot

This recipe makes two servings.

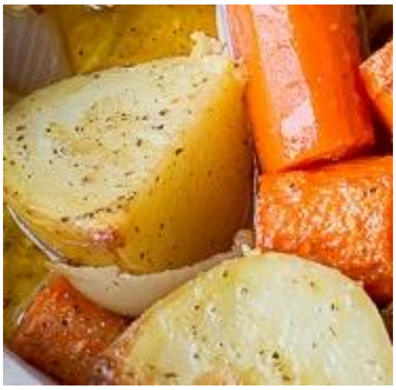

Ingredients:

1 teaspoon canola oil
1 small brown onion
1 clove garlic
1 celery stalk (or ½ courgette)
1 carrot
2 potatoes
1 red capsicum
400 g cannellini beans
2 cups vegetable stock
2 teaspoons paprika
Salt and pepper to taste

Preparation:

(1) Heat the oil in a large pan over medium heat.
(2) Chop the onion and garlic and add them to the pan. Cook 2-3 minutes.
(3) Chop the celery, carrot, potatoes, and capsicum and add them to the pan. Cook another 6-7 minutes, stirring frequently. Transfer everything to a large pot.
(4) Drain and rinse the beans, then add the beans, stock, paprika, salt, and pepper to the pot. Raise the stove to high heat and bring to a boil, then reduce to low heat and simmer for 40 minutes with the lid on. Ready to serve.

Nutrition Information:

Calories: 832.5 (416.3 per serving)
Fat: 6.1 g (3.1 g per serving)
Protein: 33.9 g (17 g per serving)
Net Carbs: 137.1 g (68.6 g per serving)
Fibre: 32.7 g (16.4 g per serving)

Bean Burritos

This recipe makes two servings (one serving = one burrito).

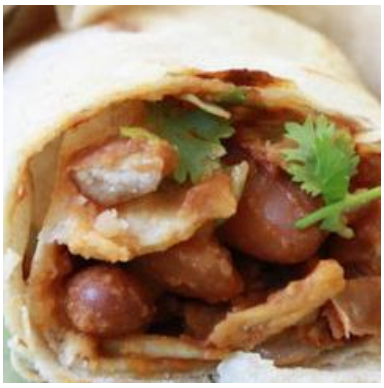

Ingredients:

2 Old El Paso regular size tortillas
400 g canned kidney beans
½ teaspoon chili powder
½ cup salsa
30 g cheddar cheese
2 spring onion stalks
¼ cup fresh coriander (chopped)
3 tablespoons light sour cream

Preparation:

(1) Preheat the oven to 180°C.
(2) Drain and rinse the kidney beans.
(3) Mash the beans with a potato masher in a large bowl. Add the chili powder and half the salsa - do not use the other half yet. Mix well.
(4) Place the tortillas on a large plate. Spoon half of the bean mixture onto each tortilla, down the centre but not to the edge.
(5) Grate the cheese and chop up the spring onion. Sprinkle half of the cheese, half of the spring onion, and half of the coriander over each tortilla. Fold up the tortillas.
(6) Place the tortillas on an ungreased baking tray, seam side down. Bake in the oven for 10-15 minutes. Remove and top with sour cream and the rest of the salsa. Serve!

Nutrition Information:

Calories: 791 (395.5 per serving)
Fat: 23.9 g (12 g per serving)
Protein: 36.1 g (18.1 g per serving)
Net Carbs: 95.3 g (47.7 g per serving)
Fibre: 17.2 g (8.6 g per serving)

Vermicelli & Homestyle Tomato Sauce

This recipe makes one serving.

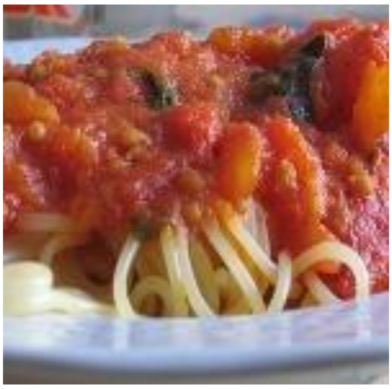

Ingredients:

2 teaspoons canola oil
¼ small brown onion
1 clove garlic
1 tomato
2 tablespoons tomato paste
½ teaspoon brown sugar
¼ teaspoon salt
¼ teaspoon black pepper
1 teaspoon basil
1 teaspoon thyme
1 teaspoon oregano
1 tablespoon water
60 g (2 cm diameter) San Remo vermicelli

Preparation:

(1) Heat the oil in a pan over medium heat.
(2) Add the chopped onion and cook until soft and clear, about 5 minutes.
(3) Add the minced garlic and cook for 30-60 seconds.
(4) Stir in the chopped tomato, tomato paste, sugar, spices, and water. Turn the heat down to low and simmer for 10-12 minutes, stirring every 1-2 minutes.
(5) To make the vermicelli, heat a pot containing 4-5 cups of water over high heat to boiling. Slowly add the vermicelli while maintaining a rapid boil, then cook 5-6 minutes; do not cover the pot. You can add one tablespoon of canola oil to prevent vermicelli sticking to the pot.
(6) Drain the vermicelli, pour the sauce on top, and enjoy!

Nutrition Information:

Calories: 389
Fat: 10.3 g
Protein: 11.3 g
Net Carbs: 60.6 g
Fibre: 2 g

Tuna Casserole

This recipe makes three servings.

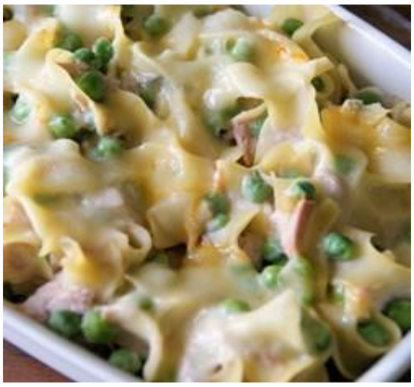

Ingredients:

¾ cup penne (uncooked)
1 cup frozen peas
1 teaspoon Anchor lite dairy blend
½ small brown onion
1 celery stalk (or ½ courgette)
210 g cream of mushroom soup
½ cup Value trim low fat milk
190 g canned tuna in spring water
1 teaspoon thyme

Preparation:

(1) Preheat the oven to 180°C.
(2) Measure 4 cups of water into a pot and bring to a boil. Once it boils, add the penne and cook for 5-6 minutes, then add the peas. When it simmers again, reduce the heat to low-medium and simmer 2-3 minutes. Drain the noodles and peas mixture and set it aside.
(3) Melt the dairy blend in a pan over medium heat and add the chopped onion and celery. Cook the onion and celery until they are tender, about 5 minutes. Set aside.
(4) Mix the cream of mushroom soup with the milk in a large bowl, then add the noodles, vegetables, tuna, and thyme. Mix everything together well.
(5) Transfer the mixture into a casserole dish and bake for 20-25 minutes, or until brown on top. Cool and serve!

Nutrition Information:

Calories: 910.6 (303.5 per serving)
Fat: 19.9 g (6.6 g per serving)
Protein: 78.5 g (26.2 g per serving)
Net Carbs: 94.6 g (31.5 g per serving)
Fibre: 12.5 g (4.2 g per serving)

Roast Vegetables

This recipe makes three servings.

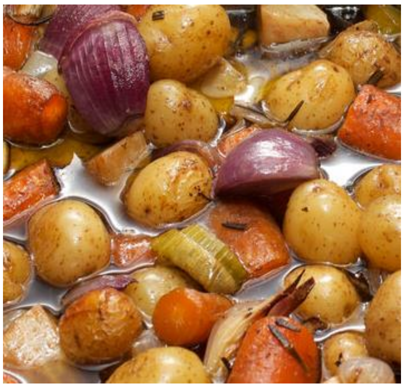

Ingredients:

1 tablespoon maple syrup (or honey)
1 teaspoon cinnamon
1 tablespoon minced ginger
1 teaspoon canola oil
1 cup vegetable stock
Salt and pepper to taste

1 small brown onion
1 kumara (sweet potato)
1 carrot
1 parsnip
1 potato
¼ cup prunes

Preparation:

(1) Preheat the oven to 180°C.
(2) Mix the maple syrup, cinnamon, ginger, oil, stock, salt, and pepper together in a small bowl to create a liquid mixture. Set aside.
(3) Chop the onion, kumara (your choice whether to peel it or not), carrot, parsnip, potato, and prunes into large chunks and place them into a large bowl.
(4) Pour the liquid mixture over the vegetables and stir until they are evenly coated.
(5) Place the vegetables in a baking dish and pour the remaining liquid mixture on top.
(6) Cover the baking dish and roast for 30 minutes, then uncover the baking dish and roast for another 15 minutes. Serve!

Nutrition Information:

Calories: 715.5 (238.5 per serving)
Fat: 6.1 g (2 g per serving)
Protein: 11.5 g (3.8 g per serving)
Net Carbs: 132.2 (44.1 g per serving)
Fibre: 23.1 g (7.7 g per serving)

Homestyle Vegetable Lasagne

This recipe makes three servings.

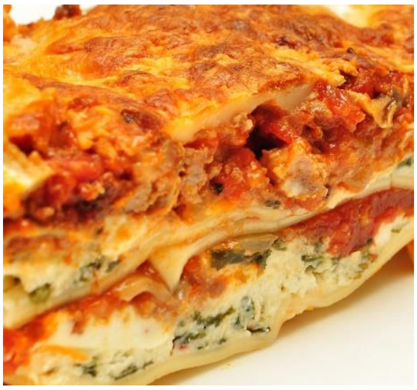

Ingredients:

2 servings Homestyle Tomato Sauce
½ cup lite cottage cheese
60 g spinach (or kale) leaves
1 egg
1 garlic clove
¼ teaspoon salt
¼ teaspoon black pepper
1 teaspoon canola oil
½ small brown onion
4 small mushrooms
3 Diamond instant lasagne sheets
60 g mozzarella cheese

Preparation:

(1) Preheat the oven to 190°C.
(2) Double the Homestyle Tomato Sauce recipe and make it now, without the vermicelli. When the sauce is halfway done, increase the heat to low-medium, add the chopped onions and mushrooms, and simmer another 10 minutes. Set aside.
(3) Use your hands to mix together the cottage cheese, spinach, egg, minced garlic, salt, and pepper in a large bowl. Set aside.
(4) Spoon one third of the sauce into a casserole dish. Cover with one lasagne sheet (cut and add part of a second sheet if needed to fit the dish). Spread another third of the sauce over the sheet, then cover with another sheet. Spread the cheese and spinach mixture over the sheet and cover with the last sheet. Spoon the final third of the lasagne sauce over the sheet and top with grated mozzarella cheese.
(5) Bake uncovered for 30-35 minutes until the cheese is melted. Serve.

Nutrition Information:

Calories: 1115 (371.7 per serving)
Fat: 45.4 g (15.1 g per serving)
Protein: 56.7 g (18.9 g per serving)
Net Carbs: 109 g (36.3 g per serving)
Fibre: 9.8 g (3.3 g per serving)

Side Dishes

Spanish Rice

This recipe makes one serving.

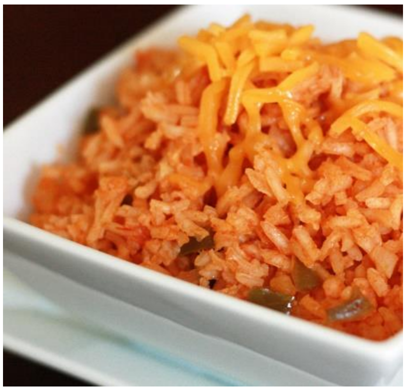

Ingredients:

2 teaspoons canola oil
¼ small brown onion
½ clove garlic
½ cup basmati rice (uncooked)
1 cup chicken stock
2 teaspoons tomato paste
Pinch of oregano
Dash of salt

Preparation:

(1) Heat the oil in a large pan set to medium high heat, then add the rice and stir until much of the rice has browned.
(2) Chop up and add the onion and garlic, and cook another 2-3 minutes, then add the stock, tomato paste, oregano, and salt.
(3) Reduce heat to low medium and simmer with the cover on for 15-25 minutes, until the rice has absorbed the stock. Remove from heat and cool for 5 minutes. Serve!

Nutrition Information:

Calories: 445.7
Fat: 9.3 g
Protein: 7.3 g
Net Carbs: 80.3 g
Fibre: 1.5 g

Indian Rice

This recipe makes four servings.

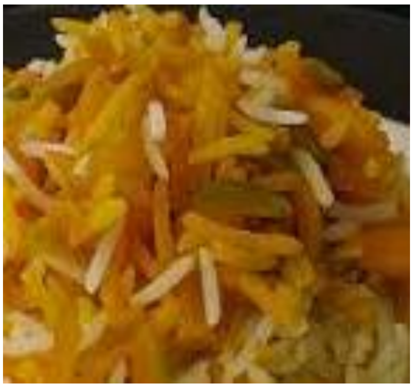

Ingredients:

1 teaspoon canola oil
1 small brown onion
1 clove garlic
2 tomatoes
2 carrots

1 cup basmati rice (uncooked)
3 cups vegetable stock
2 teaspoons ground coriander
Salt and pepper to taste
60 g spinach (or kale) leaves

Preparation:

(1) Heat the oil in a large pan over low-medium heat.
(2) Chop up the onion and garlic, and heat them in the pan for 2-3 minutes.
(3) Chop up the tomatoes and grate the carrots, then add the tomatoes, carrots, rice, stock, coriander, salt, and pepper to the pan.
(4) Raise heat to high and bring to a boil, then reduce heat to low and gently simmer everything for 20-25 minutes, until the rice is tender.
(5) Fold in the spinach and serve!

Nutrition Information:

Calories: 919.5 (229.9 per serving)
Fat: 6.3 g (1.6 g per serving)
Protein: 18.7 g (4.7 g per serving)
Net Carbs: 184.9 g (46.2 g per serving)
Fibre: 12 g (3 g per serving)

Stir-Fried Vegetables

This recipe makes two servings.

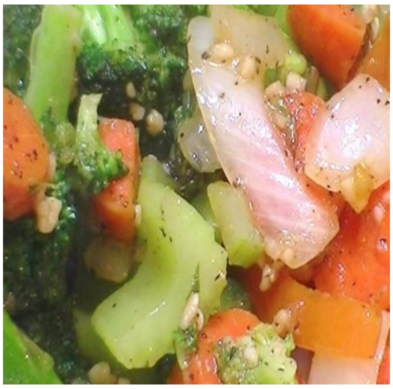

Ingredients:

1 teaspoon canola oil
1 clove garlic
1 teaspoon minced ginger
½ head of broccoli
¼ small brown onion
1 carrot
1 celery stalk (or ½ courgette)
1 teaspoon cornmeal flour
2 tablespoons cold water
1 tablespoon soy sauce
1 tablespoon honey
Black pepper to taste

Preparation:

(1) Chop up the broccoli, onion, carrot, and celery.
(2) Heat the oil in a pan over medium heat.
(3) Add the minced garlic and ginger. Saute briefly, for 30-60 seconds.
(4) Reduce to low-medium heat and add the broccoli, onion, carrot, and celery. Stir-fry until the vegetables are tender crisp, about 5-6 minutes.
(5) While the vegetables are cooking, mix the cornmeal flour, water, soy sauce, and honey in a small bowl.
(6) When the vegetables are done, stir in the sauce mixture and add pepper to taste. Cook and stir until the sauce thickens, about 2-3 minutes. Serve immediately.

Nutrition Information:

Calories: 198 (99 per serving)
Fat: 4.7 g (2.4 g per serving)
Protein: 4.9 g (2.5 g per serving)
Net Carbs: 32.4 g (16.2 g per serving)
Fibre: 5.7 g (2.9 g per serving)

Steamed Broccoli

This recipe makes one serving.

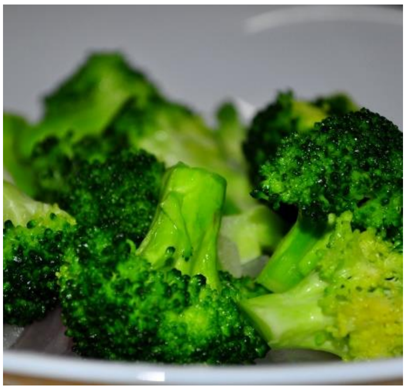

Ingredients:

½ head of broccoli

Salt to taste

Preparation:

(1) Boil some water in a pot.
(2) Chop the broccoli into florets and add them to the water.
(3) Cook until desired doneness is reached.
(4) Remove to a plate and add salt to taste.

Nutrition Information:

Calories: 39
Fat: 0 g
Protein: 3 g
Net Carbs: 5 g
Fibre: 3 g

Low Fat Vegetable Medley

This recipe makes two servings.

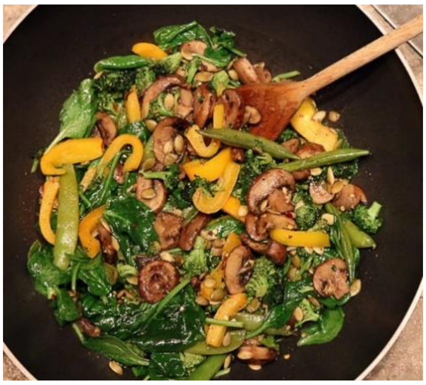

Ingredients:

2 teaspoons canola oil
1 clove garlic
2 small mushrooms
¼ head of broccoli
½ green capsicum
2 tablespoons raisins
30 g spinach (or kale) leaves

Preparation:

(1) Prepare all the vegetables by chopping them into bite-size pieces.
(2) Heat the oil in a pan on high heat. Once hot, add the minced garlic and mushrooms. Let the mushrooms soak up all the oil and cook for 1-2 minutes.
(3) Add the broccoli and mix it all together. Cook another 1-2 minutes.
(4) Add the capsicum and mix it all together. Cook another 1-2 minutes.
(5) Turn off the stove and add the raisins and spinach, but don't mix the spinach in yet - wait until it wilts, then mix in with the vegetables and serve.

Nutrition Information:

Calories: 195.5 (97.8 per serving)
Fat: 9.3 g (4.7 g per serving)
Protein: 5.6 g (2.8 g per serving)
Net Carbs: 21.3 g (10.7 g per serving)
Fibre: 5.6 g (2.8 g per serving)

Dessert

Berry Hot Drink

This recipe makes one serving.

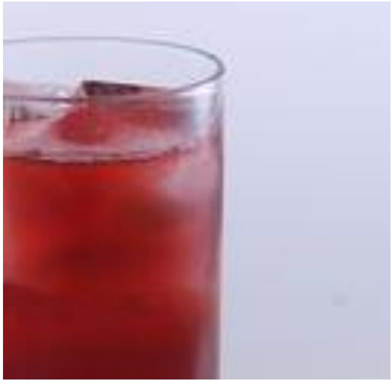

Ingredients:

¾ cup grape juice
2 tablespoons raisins
1 cup mixed berries
2 tablespoons honey (or maple syrup)

Preparation:

(1) Add the grape juice to a saucepan, bring to a boil.
(2) Meanwhile, place all the other ingredients in an electric blender.
(3) Add the boiling mix to the blender and pulse until smooth.
(4) Pour into a mug and enjoy!

Nutrition Information:

Calories: 375
Fat: 0.9 g
Protein: 2 g
Net Carbs: 90.1 g
Fibre: 6.2 g

Berry Fruit Salad

This recipe makes one serving.

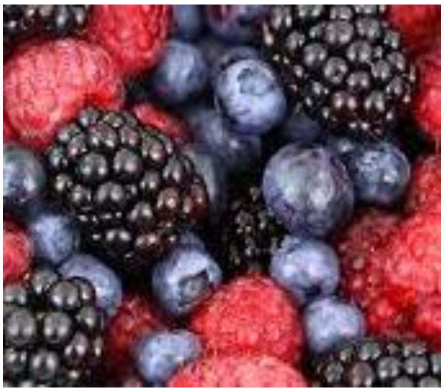

Ingredients:

1 cup strawberries
1 cup cherries (pitted)
1 cup blackberries
1 cup blueberries
1 cup raspberries
2 tablespoons mint leaves (chopped)
1 tablespoon (½ lemon) juice
1 tablespoon honey

Preparation:

(1) Cut the strawberries in half, then add the strawberries, cherries, blackberries, blueberries, raspberries, and chopped mint to a large bowl (if you can't find all the berry types in the fresh and frozen sections of the supermarket, compensate with those berry types you do have available). Set aside.
(2) Whisk together the lemon juice and honey in a separate bowl until well combined to create the dressing.
(3) Pour the dressing on top of the berries, then gently toss the fruit salad until everything is mixed together. Chill or serve, and enjoy!

Nutrition Information:

Calories: 494
Fat: 3.3 g
Protein: 7.4 g
Net Carbs: 100.2 g
Fibre: 25.5 g

Banana Bread

This recipe makes six servings (one serving = one slice).

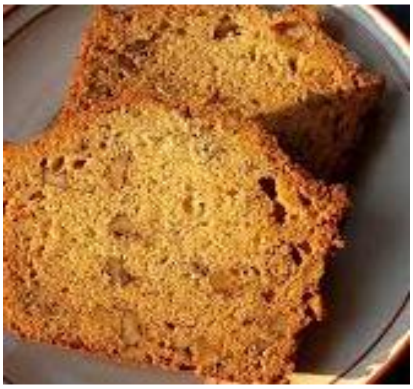

Ingredients:

¼ cup canola oil
¼ cup brown sugar
1 egg
1 teaspoon pure vanilla extract
1 tablespoon honey (or maple syrup)
2 overripe bananas
½ cup pure plain flour
¼ cup wholemeal flour
½ teaspoon baking soda
Pinch of salt

Preparation:

(1) Preheat the oven to 180°C, then grease a small baking dish (or loaf pan, if you have one) with oil.
(2) In a large bowl, mix together the oil and sugar. Add the eggs, vanilla, and honey, then mash the bananas and mix everything together as well as you can.
(3) Add all the dry ingredients and mix until just combined - do not overmix.
(4) Pour the batter into the baking dish and bake 45 minutes, or until a toothpick inserted into the centre comes out clean.
(5) Lift the loaf out of the pan and put it on a rack to cool. Enjoy!

Nutrition Information:

Calories: 1302.5 (217.1 per serving)
Fat: 61.8 g (10.3 g per serving)
Protein: 20.2 g (3.4 g per serving)
Net Carbs: 165.1 g (27.5 g per serving)
Fibre: 11.5 g (1.9 g per serving)

Lemon Lime Fruit Salad

This recipe makes two servings.

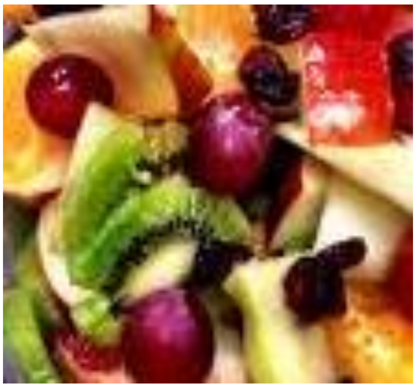

Ingredients:

450 g pineapple chunks in juice
1 orange
1 kiwi
1 cup seedless grapes
1 cup raspberries (or blueberries)
¼ teaspoon lime zest
2 tablespoons (1 lemon) juice
1 tablespoon honey

Preparation:

(1) Drain the can with the pineapple chunks, but save the juice in a small bowl (or a sealed container if you are going to work).
(2) Peel and segment the orange, then peel and chop up the kiwi. Mix the pineapple, orange, kiwi, grapes, and raspberries together in a large bowl.
(3) To zest the lime, grate the outer peel of the lime. You just want the outer layer - when you get to the white bit, roll the lime to new section of peel. Add the lime zest, lemon juice, and honey to the pineapple juice to create the dressing.
(4) When ready to eat, pour the dressing over the fruit. Toss gently and serve.

Nutrition Information:

Calories: 593.5 (296.8 per serving)
Fat: 1.6 g (0.8 g per serving)
Protein: 5.3 g (2.7 g per serving)
Net Carbs: 134.5 g (67.3 g per serving)
Fibre: 19.8 g (9.9 g per serving)

Apple Crisp

This recipe makes four servings.

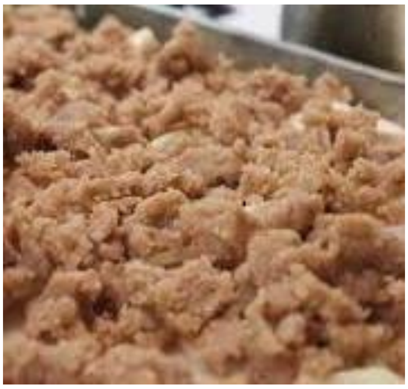

Ingredients:

2 apples
¼ cup brown sugar
½ cup wholemeal flour
½ cup wholegrain oats
1 tablespoon cinnamon
1 teaspoon nutmeg
¼ cup Anchor lite dairy blend

Preparation:

(1) Preheat the oven to 180°C.
(2) Core and cut the apples into very small slices - the smaller the better. Then, place the slices in a cake pan or small baking dish.
(3) Mix together the sugar, flour, oats, cinnamon, and nutmeg in a bowl. Melt the dairy blend for 30 seconds in a microwave, then thoroughly mix it with the dry ingredients to form the crumble mixture.
(4) Sprinkle the crumble mixture over the apple slices.
(5) Bake for 20 minutes, or until the topping is golden brown and the apples are soft. Serve and enjoy!

Nutrition Information:

Calories: 1212 (303 per serving)
Fat 43.3 g (10.8 g per serving)
Protein: 23.8 g (6 g per serving)
Net Carbs: 169.2 g (42.3 g per serving)
Fibre: 27.8 g (7 g per serving)

Sunshine Salad

This recipe makes one serving.

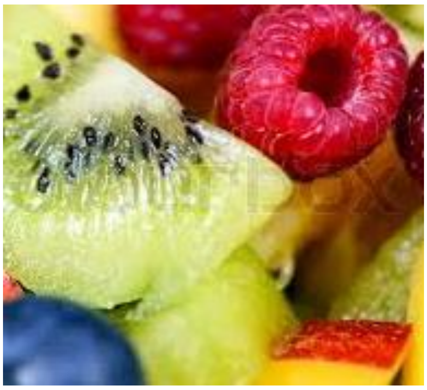

Ingredients:

1 apple
225 g pineapple chunks in juice
205 g peach slices in juice
1 banana
1½ kiwi
½ cup raspberries
½ cup blueberries

Preparation:

(1) Chop up the apples, then place them in the reserved pineapple juice in a small bowl. Let sit for 5-10 minutes.
(2) Combine the pineapple chunks and peach slices in a large bowl.
(3) Remove the apples from the juice (but keep the juice) and add them to the pineapple and peach mixture.
(4) Slice the bananas, then place them in the pineapple juice. Let sit for 5-10 minutes, then add them to the pineapple and peach mixture.
(5) Peel and slice the kiwi, then add the kiwi and berries to the mixture. Chill and serve!

Nutrition Information:

Calories: 540.1
Fat: 1.9 g
Protein: 4.9 g
Net Carbs: 119.3 g
Fibre: 17.4 g
