# Supplementary material for: Randomized crossover trial of a modified ketogenic diet in Alzheimer’s disease
Source: Alzheimers Res Ther. 2021 Feb 23;13:51. doi: 10.1186/s13195-021-00783-x (PMC7901512; doi:10.1186/s13195-021-00783-x)
Supplement: Supplementary file 1 — Additional file 1. [file 13195_2021_783_MOESM1_ESM.zip › NZ Nutrition Guidelines.pdf]

Healthy eating, active living

FOOD AND ACTIVITY ADVICE FOR ADULTS FROM 19 TO 64 YEARS

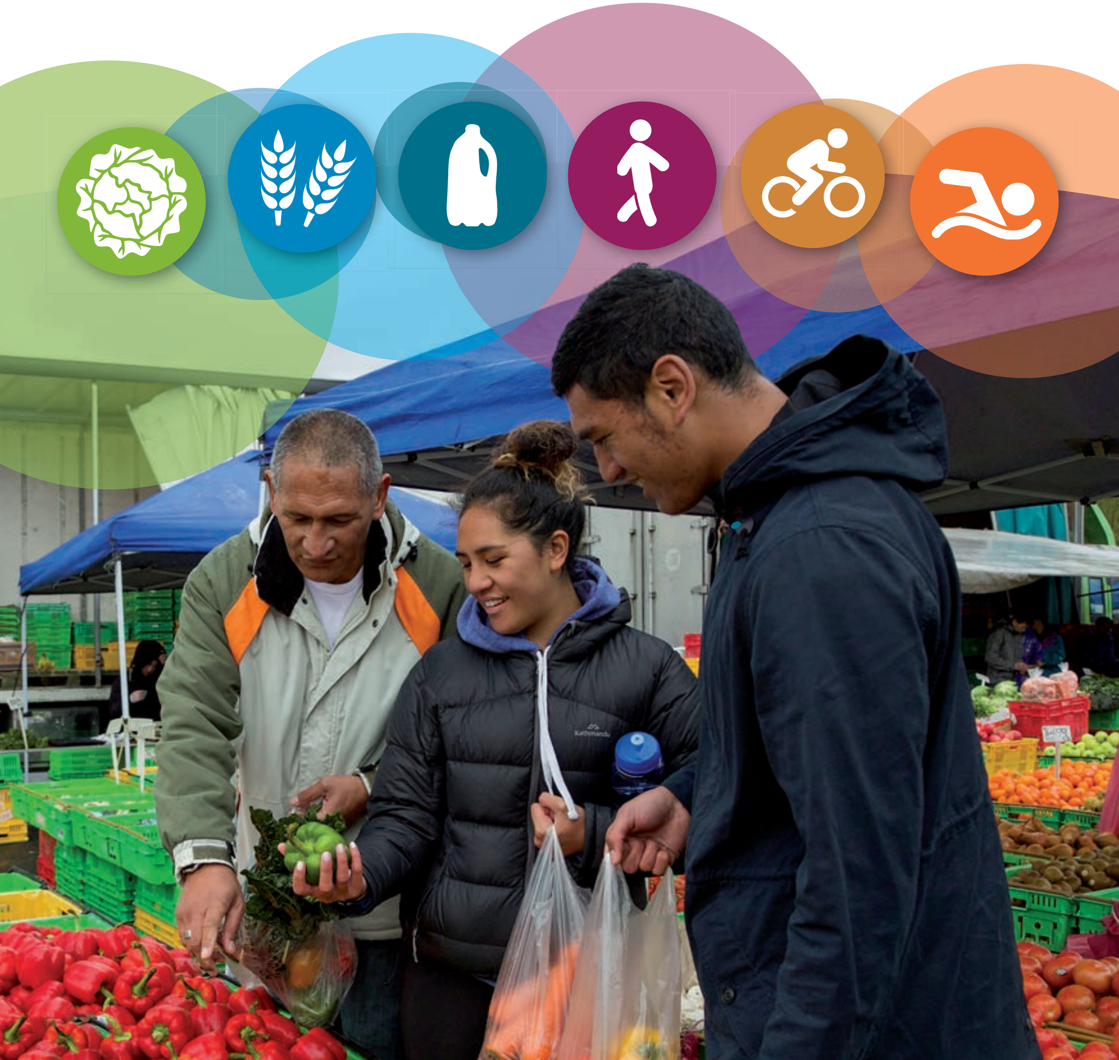

Healthy eating, active living

What and how much you eat and drink, and being physically active are important for your health. Being healthy improves your quality of life and your sense of wellbeing. Being healthy also means that you are more likely to be around longer for your whānau.

The advice in this booklet is to help you:

- choose healthy foods and drinks (pages 2–12)
- have a healthy bodyweight (page 13)
- be active in everyday life (pages 14–17).

This booklet is based on the Ministry of Health's *Eating and Activity Guidelines for New Zealand Adults.*

Healthy eating

Healthy eating:

- helps your body to work well and helps you to feel good
- can lower your risk of heart disease, stroke and some cancers and help you to have a healthy bodyweight
- means eating a variety of foods that give you the nutrients your body needs.

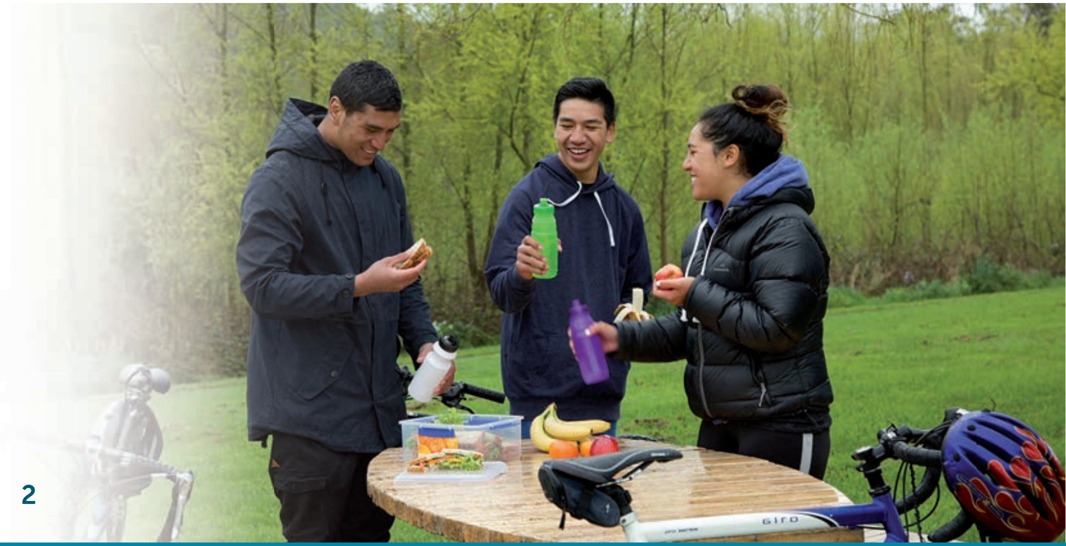

The four food groups

Enjoy a variety of nutritious foods, including:	
	Plenty of vegetables and fruit
	Grain foods, mostly whole grain and those naturally high in fibre
	Some milk and milk products, mostly low- and reduced-fat
	Some legumes*, nuts, seeds, fish and other seafood, eggs or poultry, or red meat with the fat removed.

* Legumes include lentils, split peas, chickpeas and cooked dried beans (eg, red kidney beans, baked beans).

Vegetables and fruit

▶ At least **3** servings of vegetables and at least **2** servings of fruit every day

Vegetables and fruit provide fibre, vitamins, minerals and antioxidants.

- Include them in most meals and have as a healthy snack.
- Eat many different coloured vegetables and fruit.

See **Appendix 1 on page 18** for serving sizes and examples.

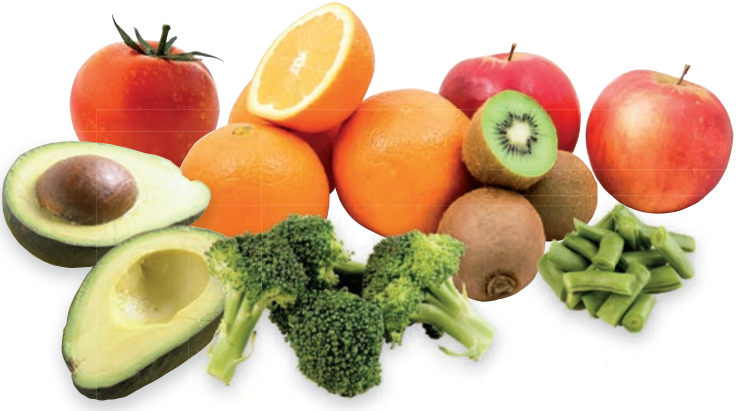

Grain foods

▶ **At least 6 servings every day – choose mostly whole grain and those naturally high in fibre.**

See **Appendix 1** on **page 18** for serving sizes and examples.

Whole grain foods are naturally high in fibre and contain vitamins and minerals. Examples include:

- whole grain bread
- porridge made with whole or rolled oats
- brown rice.

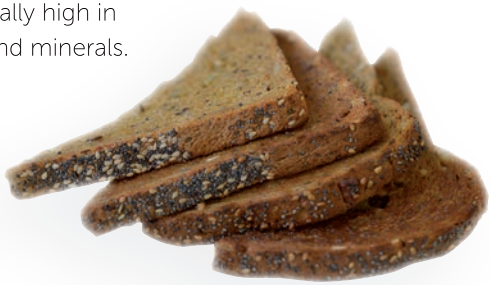

Refined grains have fewer naturally-occurring nutrients and much less fibre than whole grains. Examples of refined grains include:

- white bread
- white rice
- many breakfast cereals (eg, puffed rice).

Milk and milk products

▶ **At least 2 servings every day. Choose low-fat or reduced-fat milk and milk products.**

See **Appendix 1** on **page 18** for serving sizes and examples.

Milk, yoghurt and cheese provide protein and vitamins, and minerals including calcium.

- If you choose a plant-based milk (eg, soy, rice or almond), make sure that it has added calcium (and vitamin B12 if you avoid animal-based foods).

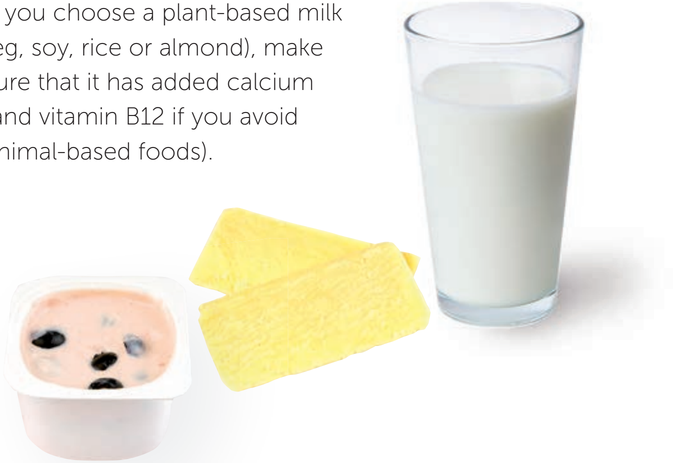

Legumes, nuts, seeds, fish and other seafood, eggs or poultry (eg, chicken), or red meat with the fat removed

▶ At least **2** servings every day of legumes, nuts or seeds **OR** at least **1** serving of seafood, eggs, poultry or red meat every day.

See **Appendix 1** on **page 18** for serving sizes and examples.

This wide range of foods all provide protein to the diet. Legumes include lentils, split peas, chickpeas and cooked dried beans (eg, red kidney beans, baked beans). Legumes, nuts and seeds are high in fibre, vitamins and minerals.

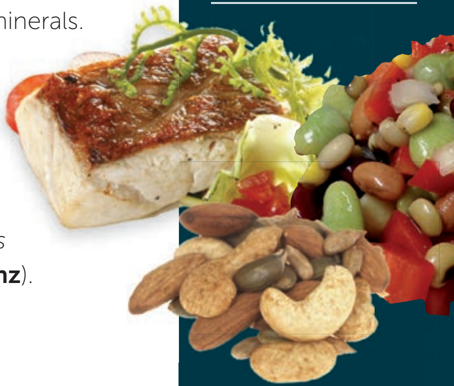

- Try to include legumes in some of your meals. For example, add lentils or a can of kidney beans to mince or a casserole.
- For more meal ideas using legumes, see the Heart Foundation's *Full O' Beans* cookbook (**www.heartfoundation.org.nz**).

Nuts and seeds are also high in healthy (unsaturated) fats (see page 7).

- Eat small amounts to avoid weight gain (see page 13).
- Choose unsalted, raw or dry-roasted nuts and seeds.

Oily fish (eg, salmon, tuna, sardines, and mackerel) and some seafood such as mussels are good sources of omega 3, which may reduce your risk of heart disease and stroke.

- Fresh or frozen fish or fish canned in spring water are all good choices.

Red meat, chicken and fish all contain iron in a form that your body can easily absorb.

- If choosing red meat, eat less than 500 g of cooked red meat a week.
- If you choose not to eat red meat, chicken or fish, see the booklet *Eating for Healthy Vegetarians* (code **HE1519**) available at **www.healthed.govt.nz**

Make healthier choices

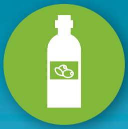

Choose and/or prepare foods:

- With unsaturated fats instead of saturated fats
- That are low in salt (sodium); if using salt, choose iodised salt
- With little or no added sugar
- That are mostly 'whole' or less processed.

As much as possible choose whole or less-processed foods. Whole foods are very close to their natural state, and they have no added fat, salt or sugar. Examples include fresh vegetables and fruit, raw nuts, fish, eggs, chicken or red meat with fat removed.

Less-processed foods have undergone some processing but still retain most of their original nutrients and can be healthy food choices. Frozen or canned vegetables and fruit, canned legumes and fish canned in spring water are healthy, convenient and affordable options. Other healthy examples include milk that is pasteurised to make it safe to drink, and whole grains such as oats, whole wheat and brown rice.

Highly-processed foods tend to be high in energy (kilojoules), added fat, sugar and/or salt but low in vitamins, minerals and fibre. Highly-processed foods include sweets, sugary drinks, biscuits, muesli bars, cakes, pastries, pies, instant noodles, salami, luncheon, chippies and store-bought burgers and pizzas. Avoid or have them only occasionally.

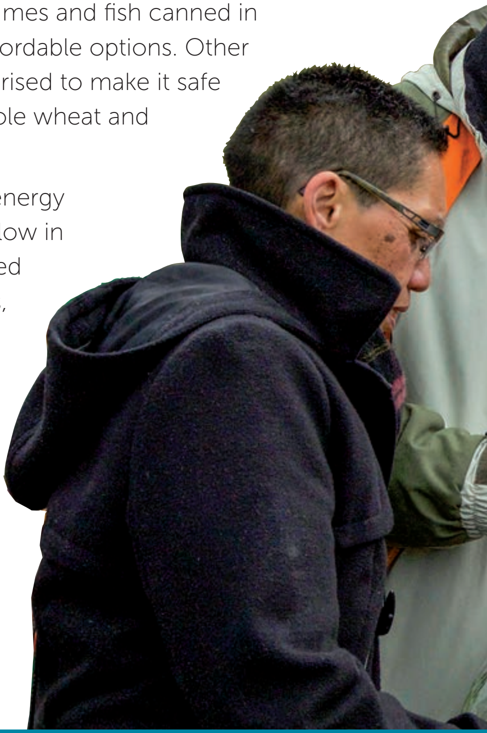

Eating too many foods that are high in saturated fat (animal fat and coconut and palm oils), sugar and salt can be bad for your health. Choosing foods with unsaturated fats (from plants, excluding coconut and palm oils) rather than saturated fat can lower your risk of heart disease.

When choosing packaged foods, look at the food product as a whole – some foods may be low in fat but high in sugar or salt. Compare the food labels of similar foods and choose those that are lower in saturated fat, sugar and/or salt and with the highest amount of fibre per 100 g of food. In the labels shown below, breakfast cereal 1 is the best choice.

Breakfast cereal 1

NUTRITION INFORMATION
Servings per package: 36
Serving size: 33 g

	Per Serve	Per 100 g
Energy (kJ)	488	1480
(Cal)	116	353
Protein (g)	4.0	12.0
Fat, total (g)	0.5	1.4
- Saturated fat (g)	0.1	0.3
Carbohydrate, total (g)	22.1	67.0
- Sugars (g)	0.9	2.8
Sodium (mg)	89	270
Dietary fibre (g)	3.3	10.1

Breakfast cereal 2

NUTRITION INFORMATION
Servings per package: 9
Serving size: 30 g

	Per Serve	Per 100 g
Energy (kJ)	477	1590
(Cal)	114	380
Protein (g)	1.9	6.3
Fat, total (g)	0.2	0.5
- Saturated fat (g)	<0.1	0.2
Carbohydrate, total (g)	25.6	85.4
- Sugars (g)	2.6	8.6
Sodium (mg)	165	550
Dietary fibre (g)	0.3	1.0

The Health Star Rating system is used on some packaged foods. You could use it to compare similar packaged foods – the more stars a food has, the healthier it is.

5 HEALTH STAR RATING

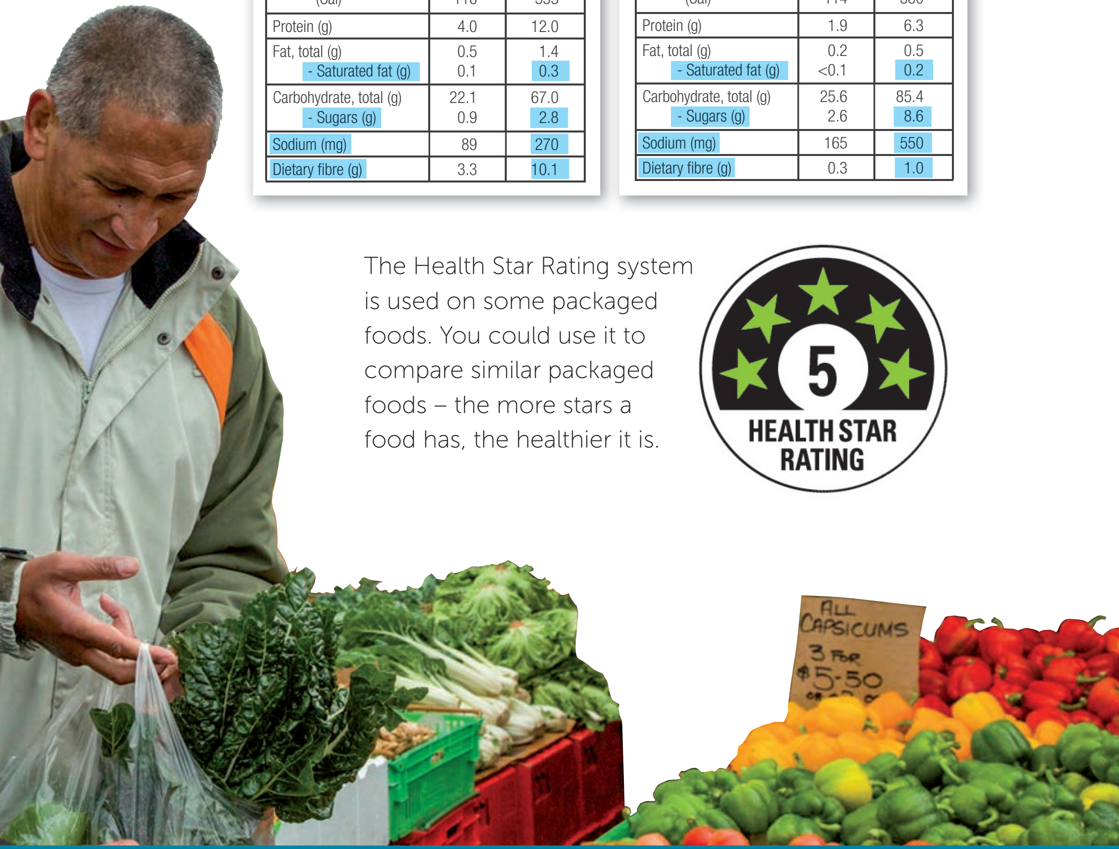

How to eat less saturated fat, sugar and salt

Cooking your own meals as often as possible is a great way to make healthy food choices. Use mostly whole or less-processed ingredients. Check out the tips below and the table opposite to see how you can eat less saturated fat, sugar and salt. For easy, fast and affordable meal ideas, see the My Family Food website (**myfamily.kiwi/foods**).

Tips for eating less saturated fat, sugar and salt

- Limit highly processed foods
- Remove the chicken skin after cooking
- Add flavour to food using herbs, spices and lemon or lime juice instead of using salt
- Use fruit to replace sugar on your breakfast cereal
- If you are adding salt, use iodised salt and try to use less salt over time
- When roasting or baking, place meat on a rack so the fat can drip off
- Choose foods with the least amount of salt. Avoid high-salt foods with more than 600 mg of sodium per 100 g of food
- Choose meat with little visible fat or remove the fat before cooking

When choosing or preparing foods

Instead of this	Use this
Butter	Margarine or other plant-based unsaturated spreads
Lard, and/or dripping	Water (for roasting meat), small amount of plant-based unsaturated oils (see below)
Coconut oil	Small amount of plant-based unsaturated oils, eg, canola, olive, sunflower or rice bran oils
Full-fat milk	Low- and reduced-fat milk
High-fat cheese eg, mild, colby	Reduced-fat cheese eg, edam
Coconut cream	'Lite' coconut cream or milk, or use half water and half coconut cream
White bread	Higher fibre, dense whole grain bread
High-fat takeaways	Healthier option takeaways, eg, kebabs or wraps with plenty of salad, non-fried Asian rice or noodle dishes with plenty of vegetables
Sugar-coated breakfast cereal	Whole grain cereal, eg, porridge or whole wheat breakfast biscuits
Muesli bars	Fresh fruit or a small handful of unsalted nuts
Chippies and a cream-based dip	Raw vegetable sticks and hummus, or homemade popcorn (go easy on the salt)
Sugary drinks	Water. It is nice chilled, with fresh mint and/or a slice of lemon
Fruit juice	Glass of chilled water and a piece of fruit
Dried fruit	Fresh fruit

What can you do to make healthier choices when choosing and preparing food?

Drinks

Make plain water your first choice over other drinks.

You need about 8–10 cups of fluid each day to keep your body hydrated and working properly.

Plain fluoridated tap water is best. Keep a jug of water in the fridge and add flavour with fresh mint or slices of lemon.

Low-fat milk is also a good drink because it contains protein and many vitamins and minerals. Soy or rice milk with added calcium are dairy-free choices.

Tea and coffee are ok but have no more than 7 cups of tea or instant coffee or 3 single shot espresso-type coffees a day.

Limit the amount of sugary drinks that you have. Fruit juices, cordials, non-diet fizzy and energy drinks all contain a lot of sugar.

Drink more water in warm weather and when you are doing lots of physical work or activity.

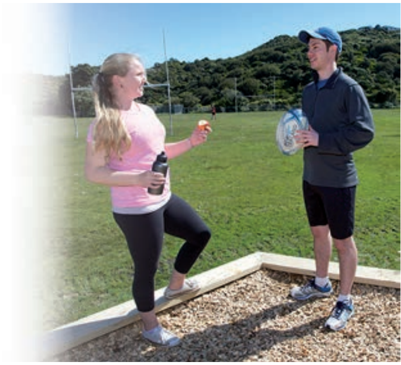

Limit alcohol

Limit alcohol

- **If you drink alcohol, keep your intake low.**
- **Stop drinking alcohol if you could be pregnant, are pregnant or are trying to get pregnant.**

Drinking too much alcohol can cause health problems as well as injury. It can also make some existing health conditions worse.

There are times when it is best not to drink alcohol at all, such as when you are pregnant, or if you are on medication that interacts with alcohol or are about to operate machinery or a vehicle.

The standard drinks measure is a simple way to work out how much alcohol we're having. One standard drink equals 10 grams of pure alcohol.

The picture below shows how many standard drinks there are in different kinds of alcohol.

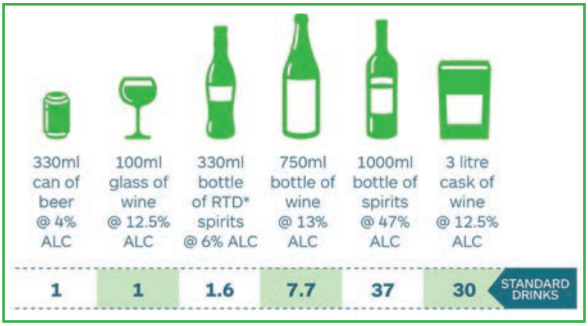

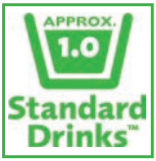

The number inside the image tells you approximately how many standard drinks are in that bottle, can or cask.

*RTD (Ready-to-drink)

All bottles of wine, beer, cider and spirits and all cans and casks are required to have standard drinks content on the labels. Look out for wording about standard drinks or for the standard drinks image.

When you pour an alcoholic drink at home, you're probably pouring different amounts every time. By checking the standard drinks label, you'll have a good idea of how many drinks you are serving out of each bottle, can or cask.

You can reduce your long-term risks and your risk of injury by following the Health Promotion Agency's low-risk alcohol drinking advice below.

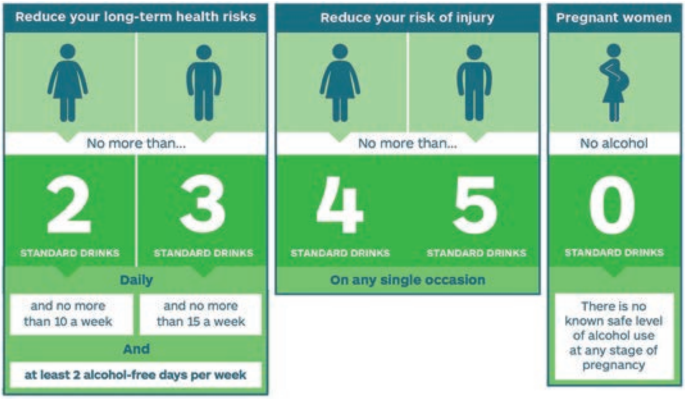

For more information about alcohol, see the Alcohol section of the Health Promotion Agency's alcohol website: **alcohol.org.nz**

Food safety

Buy or gather, prepare, cook and store food in ways that keep it safe to eat.

Food safety is about making sure that food is safe to eat. Harmful bacteria and viruses (bugs) can live in some foods, and if the food is not safely gathered, prepared, cooked or stored the bugs can make you or other people ill.

Buy safe food

- Check the "use by" date before you buy.
- Avoid foods with damaged packaging, such as dented cans or broken seals.
- Choose undamaged and unripe (or just ripe) fresh vegetables and fruit.

Gathering food

- Always wash food that you have gathered or bought from someone who has gathered it, such as pūha and watercress.
- Before gathering seafood, check with your local public health unit or the Foodsmart website (see below) that the area you will be gathering from is clean and is free from pollution.

Storing food

- Make sure the fridge temperature is at or less than 4°C.
- Store raw meat away from other food.
- Follow storage advice on labels.
- Cover leftovers and store them in the fridge (within two hours). Use leftovers within two days of cooking.

Preparing and cooking food

- Always wash and dry your hands before and after preparing food.
- Use clean surfaces and utensils to prepare foods.
- Make sure reheated food is steaming hot right through and only reheated once.
- Some foods are more likely to have harmful bugs in them, such as meat, chicken, fish, milk products, rice and legumes. Make sure you safely store and cook these foods. If in doubt, throw them out.

You can find out more about food safety on the Ministry for Primary Industries' website (**http://mpi.govt.nz/food-safety/**).

A healthy bodyweight

Making good choices about what you eat and drink and being physically active are important to achieve and keep a healthy bodyweight.

Having a healthy bodyweight is about balancing the energy (kilojoules) you eat and drink with the energy you use through physical activity. Weight gain occurs when you eat and drink more energy than you use.

To avoid gaining weight:

- eat mostly nutritious foods that are low in energy (for example, include vegetables or fruit where possible in meals and as snacks)
- drink plain water instead of sugary drinks and/or alcoholic drinks
- eat smaller portions of food
- sit less and move more
- be as active as possible.

For more information, including a guide to a healthy weight range, see the booklet *Healthy Weight for Adults* (code **HE1324**), available at **www.healthed.govt.nz**

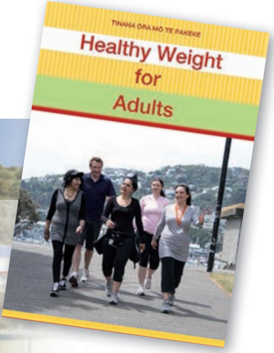

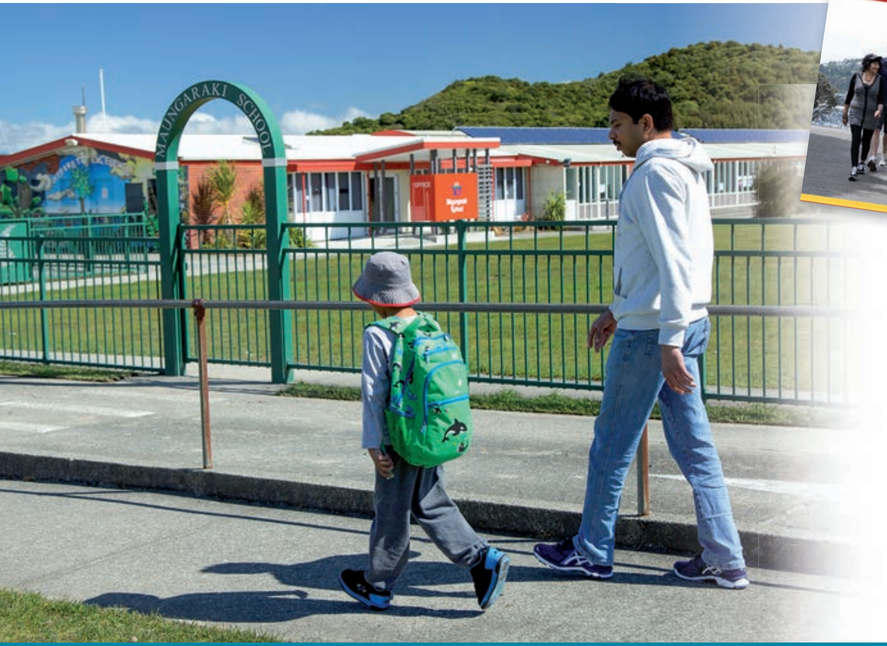

Active living

Active living means being regularly physically active, sitting less, and moving more.

Together with healthy eating, active living can lower your risk of heart disease, stroke and some cancers and help you to have a healthy bodyweight. Being active can help you feel more positive and able to keep up with children, friends and whānau. You'll feel better and be healthier.

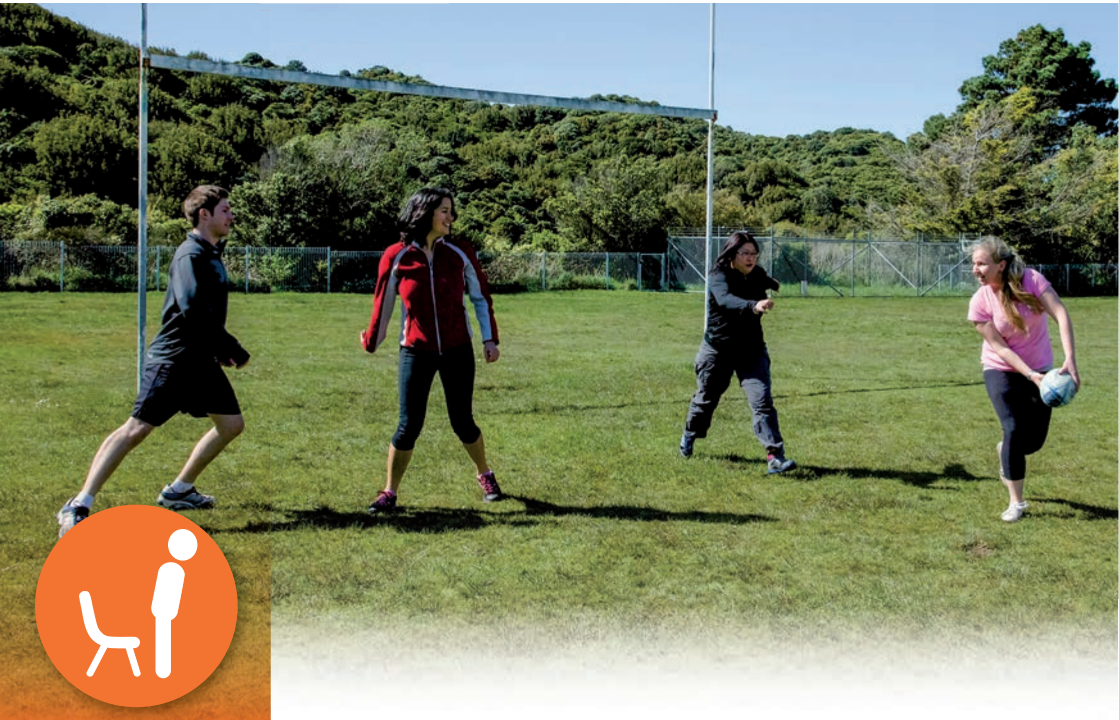

See **Appendix 2** on **page 19** for examples of great activities that you could do.

Sit less, move more! Break up long periods of sitting.

Sitting for long periods of time (even if you are already physically active) can increase your risk of diabetes, heart disease and obesity (when you have too much body fat).

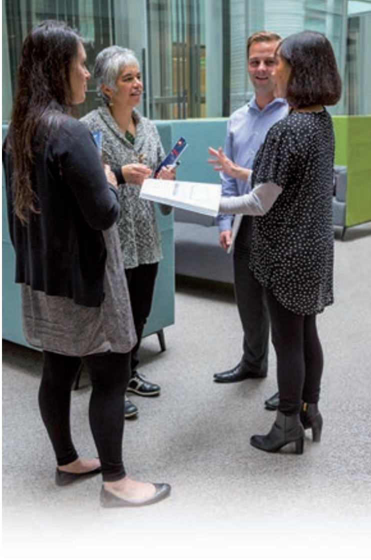

Break up long periods of sitting

- Stand up to stretch often – for at least a few minutes each hour.
- Have standing or walking meetings while you are at work.
- Stand while you are travelling on buses, trains and ferries.
- Get off the bus or train one stop earlier and walk the rest of the way, or park further away.
- Limit TV, computer and other electronic device use when you are at home. Go for a walk instead. Stand up and stretch when the ads come on TV.
- Stand up while texting or talking on the phone.

What can you do to sit less and move more?

Do at least 2 ½ hours of moderate or 1 ¼ hours of vigorous physical activity spread throughout the week.

Moderate and vigorous physical activities are great for your heart and lungs, as well as your overall fitness and wellbeing. Moderate activities include brisk walking, dancing or actively playing with children. Vigorous activities include running, biking uphill, or playing sport.

Regular moderate (or vigorous) physical activity can help you to:

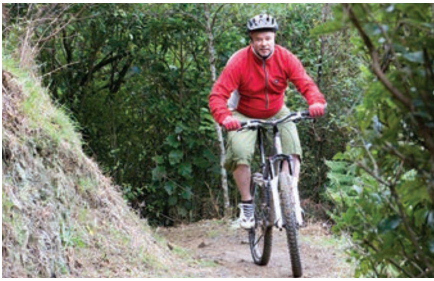

- have more energy – so you can have fun and play with your children, grandchildren, nieces or nephews
- lower your stress levels
- improve your posture and balance
- maintain a healthy weight
- keep bones and muscles strong and joints flexible
- feel more relaxed and sleep better
- reduce your risk of heart disease, obesity, strokes, diabetes, some cancers, depression and falls
- be healthier – so you're around longer for your whānau
- live independently for longer.

Why spread activity throughout the week?

Any physical activity is good, but being physically active every day is better for you than doing all your activity on one or two days a week.

You could:

- do at least 30 minutes of moderate, or 15 minutes of vigorous physical activity on five days a week
- break up your activity sessions into smaller chunks that you do more often, for example, 10 minutes at a time.

For extra health benefits, aim for 5 hours of moderate or 2 ½ hours of vigorous physical activity spread throughout the week.

Doing more physical activity has extra health benefits, including management of weight and a greater reduction in the risk of some cancers.

To do more physical activity you could:

- do a brisk walk for 1 hour instead of 30 minutes
- change from brisk walking (moderate) to running (vigorous).

You could even set yourself a goal or challenge, such as an event to complete.

Do muscle strengthening activities on at least 2 days each week.

Muscle strengthening activities will help keep your body strong and flexible so you can continue to do everyday activities such as walking, hanging out the washing, gardening and carrying heavy objects, as you get older.

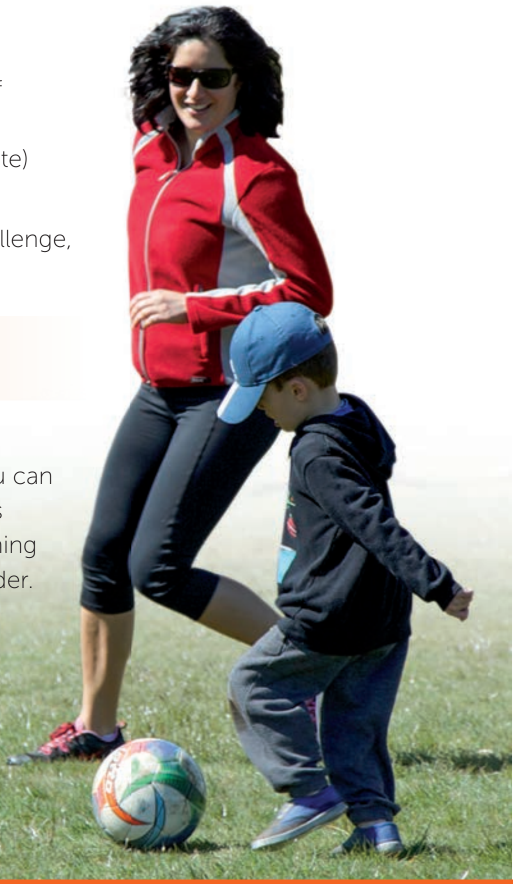

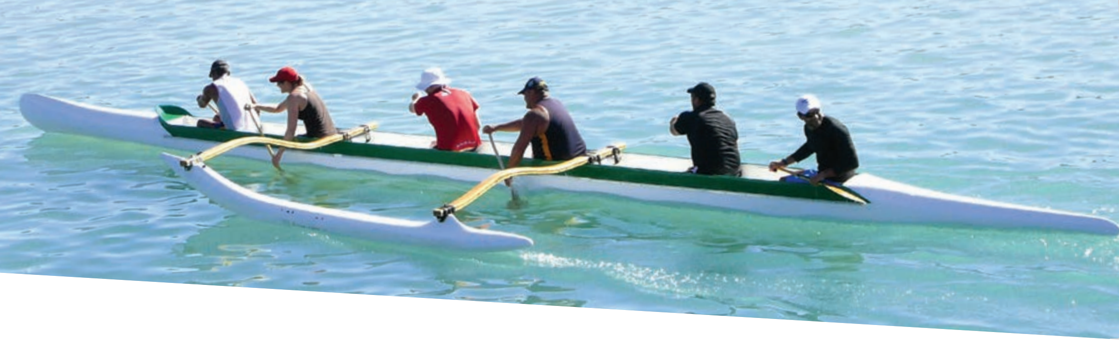

There are plenty of ways that you can do muscle strengthening activities – many of them are free and can be done anywhere. You could:

- carry young children or heavy shopping
- do push ups, sit ups or squats
- walk up hills
- go tramping, aqua jogging or to the gym to do weights
- do waka ama, oe vaka or kayaking.

Doing some physical activity is better than doing none.

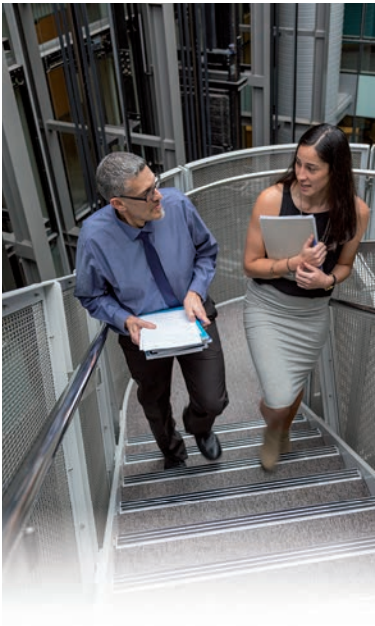

Doing any physical activity is good for your health – even if you do less than the recommended 2 ½ hours of moderate physical activity each week.

It's easy to add some physical activity to your day. You could:

- take the stairs rather than the lift
- get off the bus early and walk or park further away
- cycle or walk from place to place
- do the housework.

Choosing a physical activity that you enjoy means you're more likely to do it more often. Doing a physical activity with whānau or friends can also be a fun way to be more active.

Talk to your doctor or nurse if it's been a long time since you were active or if you have any health problems. Your doctor or practice nurse can also give you a Green Prescription – written advice on getting active and feeling better.

For fun ideas to keep your family active, visit the Healthy Kids website (**www.healthykids.org.nz**).

What activities do you enjoy doing?

Appendix 1
– Food groups and serving sizes

Food group	Advice	Serving size examples
Vegetables and fruit (includes fresh, frozen and canned)	At least 5 servings per day: at least 3 servings of vegetables and at least 2 servings of fruit	½ cup of cooked vegetables or ½ cup of salad<br>1 medium potato, or similar size piece of kumara, yam, or taro<br>1 medium apple, pear, banana or orange<br>½ cup of fresh or stewed fruit salad
Grain foods	At least 6 servings per day (mostly whole grain and those naturally high in fibre)	2 breakfast wheat biscuits<br>1 whole grain bread roll or 1 sandwich slice of whole grain bread<br>½ cup of cooked porridge/rolled oats or ½ cup of muesli<br>1 cup of cooked pasta or brown rice
Milk and milk products	At least 2 servings per day (choose low- or reduced-fat options)	1 glass (250 mL) of milk or calcium-added soy or rice milk<br>1 small pottle of yoghurt (125–150 g)<br>2 slices (40 g) of cheese (eg, edam)
Legumes*, nuts, seeds, fish and other seafood, eggs, poultry or red meat with fat removed	At least 2 servings of legumes, nuts, and seeds per day<br>**OR**<br>At least 1 serving of fish and other seafood, eggs, poultry or red meat per day	¾ cup of cooked dried beans, peas or lentils<br>Small handful (30 g) of nuts or seeds<br>1 medium fillet of cooked fish (100 g)<br>1 egg<br>2 chicken drumsticks or 1 chicken leg<br>2 slices of cooked meat (100 g) (eg, roast lamb, chicken, beef or pork)<br>¾ cup of mince or casserole

* Legumes include lentils, split peas, chickpeas and cooked dried beans (eg, red kidney beans, baked beans)

Appendix 2
– Moderate, vigorous and muscle strengthening activities

Do at least 2 ½ hours of moderate or 1 ¼ hours of vigorous physical activity spread throughout the week, plus muscle strengthening activities on at least 2 days each week. For extra health benefits, aim for 5 hours of moderate (or 2 ½ hours of vigorous) physical activity spread throughout the week.

**Moderate activity:** I'm breathing faster and my heart is beating a bit more. I can still talk.	**Vigorous activity:** I'm breathing much harder and my heart is beating faster. I can only say a few words without taking a breath.	**Muscle strengthening activity:** I can feel my muscles working harder.
Actively playing with children	Rope skipping*	Carrying young children
Vacuuming, washing the car	Heavy home repair, moving heavy furniture	Carrying heavy shopping bags
Strenuous gardening* – carrying loads in a wheelbarrow or digging	Heavy digging, chopping wood	Sawing
Brisk walking, stair climbing*	Hill walking,* running*	Tramping
Biking on the flat	Biking faster than 16 km per hour*	Uphill mountain biking
Aerobics	High-intensity intermittent training*	Push ups, sit ups, squats or weight training
Surfing	Fast swimming*	Water aerobics, water jogging
Sports, eg, cricket, kilikiti, surfing, golf (carrying your bag)*	Sports, eg, waka ama*, rugby, ki o Rahi, football, netball, touch, hockey, tennis	Sports, eg oe vaka, kayaking, canoeing

* These activities will also strengthen your muscles.

More information

For more information about healthy eating and active living speak to your doctor or nurse. You can also visit the following websites.

HealthEd – see the Healthy Eating and Physical Activity health topics
- **www.healthed.govt.nz**

Ministry of Health: Food, physical activity and sleep
- **www.health.govt.nz/your-health/healthy-living/food-activity-and-sleep**

This resource is available from www.healthed.govt.nz or the Authorised Provider at your local DHB.
Revised October 2015. 04/2018. Code **HE1518.**

ISBN 978-0-478-41153-9
HE1518 Healthy eating, active living (print)

ISBN 978-0-478-41154-6
HE1518 Healthy eating, active living (online)

MINISTRY OF HEALTH
MANATŪ HAUORA

hpa health promotion agency

New Zealand Government
