# Supplementary material for: Randomized crossover trial of a modified ketogenic diet in Alzheimer’s disease
Source: Alzheimers Res Ther. 2021 Feb 23;13:51. doi: 10.1186/s13195-021-00783-x (PMC7901512; doi:10.1186/s13195-021-00783-x)
Supplement: Supplementary file 1 — Additional file 1. [file 13195_2021_783_MOESM1_ESM.zip › The Ketogenic Way - Alzheimers Dietary Study.pdf]

The Ketogenic Way

Ketogenic Diet Recipes

The Alzheimer’s Dietary Study

2019-2020

Table Of Contents

The Commitment….3

Your Nightly Record (Write Down Your Numbers)….5

Ketogenic Recipes….9

Lazy Days (Eating In)….100

Lazy Days (Eating Out)….101

The Commitment

“Let food be thy medicine, and medicine be thy food.”
- Hippocrates

The ketogenic diet is a high-fat, adequate-protein, low-carb diet that aims to put your body into an **altered metabolic state**. The ketones produced by this state have many theoretical advantages over glucose, especially as fuel for neurons; compared to glucose, ketones produce more energy and spur the growth of neurons, which is what we want for Alzheimer’s.

The potential power of this meal plan does not stem from one single meal or day, but rather, from **sticking to it** for a sustained length of time, keeping carbohydrates low in a sustained fashion, so that your body is orchestrated into an altered metabolic state. Such a state may be extremely susceptible to even small deviations from the meal plan - thus, temptation is the enemy in this study. Please try your best not to deviate from the plan!

Generally, most **temptations** will take one of two forms:

(1) Temptation from yourself.
You will experience cravings for carbohydrates, especially sugar. The best way to prepare for this is to remove all carbs and sugar from the kitchen. Pack them into a big box and stuff them in the garage! Believe me, this will make things much easier for you.

(2) Temptation from others.
Well-meaning family and friends may offer you foods that could disrupt your metabolic state. These offerings may seem harmless, but even a small amount of carbohydrate may hijack your state of ketosis. The best response is to politely decline; hopefully, they will understand.

There are five **guidelines** that will confer success with this meal plan:

(1) **Eat at least one serving of breakfast, lunch, dinner, and a side dish every day** - Aim to have at least one serving of each meal, and more if you like (as long as it is from this plan). We don't want you to lose too much weight, and the best way to keep up your weight is to eat more; eating more will also push your ketones higher.

(2) **Drink at least 5 cups of water a day, and use adequate salt** - We must make sure you do not become dehydrated or low in salt, which can happen on a ketogenic diet and may lead to low blood pressure, dizziness, and falls. Drink (and tick) the 5 cups of water every day, and make sure you use extra salt on all your food.

(3) **Limited black coffee and red wine are ok** - If you drink coffee, limit it to 2-3 cups black coffee per day (no milk or sugar), or you can try adding 1 tbsp coconut oil or cream to it. If you like wine, please limit to half a cup (125 ml) red wine per day, maximum (absolutely no white wine or beer please).

(4) **Record your bedtime glucose and ketones** - Check your blood glucose and ketones every night at bedtime, then write the numbers in the boxes on pages 5-8. Try to keep the ketones **over 1 mmol/L**, if you can!

(5) **If you don't feel like cooking, it's ok** - Sometimes, it's nice to have a break from cooking and eat at a restaurant or social function! However, please abide by the *Lazy Days* food and meal suggestions at the end of this plan to determine which meals are "ok" and which are "not ok."

You may email me at Matthew.Phillips@waikatodhb.health.nz any time. You may also email Deborah at nutritioninalzheimers@gmail.com any time.

We are trying to get your body into an altered metabolic state. To succeed, **make a commitment with yourself** - stick to the plan!

Food is the real medicine.

Sincerely,

Matt, Deborah, Stacey, Grace…and the rest of the Alzheimer's Dietary Study team.

Your Nightly Record (Write Down Your Numbers, Every Night At Bedtime)

Week 1

February 17, 2020	Bedtime Glucose		Bedtime Ketones	
February 18, 2020	Bedtime Glucose		Bedtime Ketones	
February 19, 2020	Bedtime Glucose		Bedtime Ketones	
February 20, 2020	Bedtime Glucose		Bedtime Ketones	
February 21, 2020	Bedtime Glucose		Bedtime Ketones	
February 22, 2020	Bedtime Glucose		Bedtime Ketones	
February 23, 2020	Bedtime Glucose		Bedtime Ketones	

Week 2

February 24, 2020	Bedtime Glucose		Bedtime Ketones	
February 25, 2020	Bedtime Glucose		Bedtime Ketones	
February 26, 2020	Bedtime Glucose		Bedtime Ketones	
February 27, 2020	Bedtime Glucose		Bedtime Ketones	
February 28, 2020	Bedtime Glucose		Bedtime Ketones	
February 29, 2020	Bedtime Glucose		Bedtime Ketones	
March 1, 2020	Bedtime Glucose		Bedtime Ketones	

Week 3

March 2, 2020	Bedtime Glucose		Bedtime Ketones	
March 3, 2020	Bedtime Glucose		Bedtime Ketones	
March 4, 2020	Bedtime Glucose		Bedtime Ketones	
March 5, 2020	Bedtime Glucose		Bedtime Ketones	
March 6, 2020	Bedtime Glucose		Bedtime Ketones	
March 7, 2020	Bedtime Glucose		Bedtime Ketones	
March 8, 2020	Bedtime Glucose		Bedtime Ketones	

Week 4

March 9, 2020	Bedtime Glucose		Bedtime Ketones	
March 10, 2020	Bedtime Glucose		Bedtime Ketones	
March 11, 2020	Bedtime Glucose		Bedtime Ketones	
March 12, 2020	Bedtime Glucose		Bedtime Ketones	
March 13, 2020	Bedtime Glucose		Bedtime Ketones	
March 14, 2020	Bedtime Glucose		Bedtime Ketones	
March 15, 2020	Bedtime Glucose		Bedtime Ketones	

Week 5

March 16, 2020	Bedtime Glucose		Bedtime Ketones	
March 17, 2020	Bedtime Glucose		Bedtime Ketones	
March 18, 2020	Bedtime Glucose		Bedtime Ketones	
March 19, 2020	Bedtime Glucose		Bedtime Ketones	
March 20, 2020	Bedtime Glucose		Bedtime Ketones	
March 21, 2020	Bedtime Glucose		Bedtime Ketones	
March 22, 2020	Bedtime Glucose		Bedtime Ketones	

Week 6

March 23, 2020	Bedtime Glucose		Bedtime Ketones	
March 24, 2020	Bedtime Glucose		Bedtime Ketones	
March 25, 2020	Bedtime Glucose		Bedtime Ketones	
March 26, 2020	Bedtime Glucose		Bedtime Ketones	
March 27, 2020	Bedtime Glucose		Bedtime Ketones	
March 28, 2020	Bedtime Glucose		Bedtime Ketones	
March 29, 2020	Bedtime Glucose		Bedtime Ketones	

Week 7

March 30, 2020	Bedtime Glucose		Bedtime Ketones	
March 31, 2020	Bedtime Glucose		Bedtime Ketones	
April 1, 2020	Bedtime Glucose		Bedtime Ketones	
April 2, 2020	Bedtime Glucose		Bedtime Ketones	
April 3, 2020	Bedtime Glucose		Bedtime Ketones	
April 4, 2020	Bedtime Glucose		Bedtime Ketones	
April 5, 2020	Bedtime Glucose		Bedtime Ketones	

Week 8

April 6, 2020	Bedtime Glucose		Bedtime Ketones	
April 7, 2020	Bedtime Glucose		Bedtime Ketones	
April 8, 2020	Bedtime Glucose		Bedtime Ketones	
April 9, 2020	Bedtime Glucose		Bedtime Ketones	
April 10, 2020	Bedtime Glucose		Bedtime Ketones	
April 11, 2020	Bedtime Glucose		Bedtime Ketones	
April 12, 2020	Bedtime Glucose		Bedtime Ketones	

Week 9

April 13, 2020	Bedtime Glucose		Bedtime Ketones	
April 14, 2020	Bedtime Glucose		Bedtime Ketones	
April 15, 2020	Bedtime Glucose		Bedtime Ketones	
April 16, 2020	Bedtime Glucose		Bedtime Ketones	
April 17, 2020	Bedtime Glucose		Bedtime Ketones	
April 18, 2020	Bedtime Glucose		Bedtime Ketones	
April 19, 2020	Bedtime Glucose		Bedtime Ketones	

Week 10

April 20, 2020	Bedtime Glucose		Bedtime Ketones	
April 21, 2020	Bedtime Glucose		Bedtime Ketones	
April 22, 2020	Bedtime Glucose		Bedtime Ketones	
April 23, 2020	Bedtime Glucose		Bedtime Ketones	
April 24, 2020	Bedtime Glucose		Bedtime Ketones	
April 25, 2020	Bedtime Glucose		Bedtime Ketones	
April 26, 2020	Bedtime Glucose		Bedtime Ketones	

Week 11

April 27, 2020	Bedtime Glucose		Bedtime Ketones	
April 28, 2020	Bedtime Glucose		Bedtime Ketones	
April 29, 2020	Bedtime Glucose		Bedtime Ketones	
April 30, 2020	Bedtime Glucose		Bedtime Ketones	
May 1, 2020	Bedtime Glucose		Bedtime Ketones	
May 2, 2020	Bedtime Glucose		Bedtime Ketones	
May 3, 2020	Bedtime Glucose		Bedtime Ketones	

Week 12

May 4, 2020	Bedtime Glucose		Bedtime Ketones	
May 5, 2020	Bedtime Glucose		Bedtime Ketones	
May 6, 2020	Bedtime Glucose		Bedtime Ketones	
May 7, 2020	Bedtime Glucose		Bedtime Ketones	
May 8, 2020	Bedtime Glucose		Bedtime Ketones	

BREAKFAST..10
Avocado Smoothie..11
Chia Pudding..12
Berry Smoothie..13
Keto Porridge..14
Bacon & Eggs..15
Keto Pancakes..16
Spinach, Onion, & Goat Cheese Omelette..17
Keto Granola..18
Kale, Chorizo, & Eggs..19
Bacon Avocado Muffins..20
Vanilla Pecan Bars..21

LUNCH (SALADS)..22
Raspberry Arugula Salad..23
Strawberry Kale Salad..24
Greek Salad..25
Caprese Salad..26
Mediterranean Salad..27
Bacon, Broccoli, & Almond Salad..28
Salmon Salad..29
Basil Pesto Chicken Salad..30

LUNCH (STEWS & SOUPS)..31
Crockpot Chicken Stew..32
Crockpot Steak Stew..33
Keto Hamburger Soup..34
Chicken Enchilada Soup..35
Thai Coconut Chicken Soup..36

LUNCH (SANDWICHES & BREADS)..37
Keto Cheeseburgers..38
Chicken Avocado Sandwiches..39
Light Keto Bread..40
Sarona's Keto Bread..41
Keto Nutloaf..42

DINNER (EUROPEAN)..43
Keto Pizza..44
Caprese Chicken..45
Tuscan Salmon..46
Lemony Gurnard..47
Bacon & Basil Pesto Mushroom Cups..48
Seasoned Salmon Fry..49
Tarragon Dill Salmon..50
Chicken Bacon Drumsticks..51
Greek Chicken..52

DINNER (PAN-AMERICAN)..53
Bacon Avocado Wedges..54
Salmon Con Salsa..55
Chicken Fajita Bowl..56
Reverse Seared Steak..57
Steak Fajitas..58
Tex-Mex Casserole..59

DINNER (EAST ASIAN)..60
Pushpa's Miti Fish..61
Sticky Chicken Stirfry..62
Thai Coconut Fish Curry..63
Beef & Cashew Stirfry..64

DINNER (SOUTH ASIAN)..65
Palak Paneer..66
Butter Chicken..67
Keto Paneer Makhanwala..68
Salmon Curry..69
Chicken Korma..70
Indian Eggplant..71
Lamb Curry..72
Vegetable & Cashew Korma..73
Chicken Curry Drumsticks..74

SIDE DISHES..75
Real Mayonnaise..76
Keto Guacamole..77
Plain Cauliflower Rice..78
Indian Cauliflower Rice..79
Marinated Olives..80
Worcester Brussels Sprouts..81
Warm Kale Salad..82
Buttered Broccoli..83
Keto Asparagus..84
Garlic Spinach..85
Asian Broccoli Slaw..86
Lemon Roasted Broccoli..87
Vegetable Medley..88

DESSERT..89
Chocolate Shake..90
Hot Cocoa..91
Dark Chocolate Pudding..92
Berries & Cream..93
Chocolate Cupcakes..94
Cinnamon Butter Cookies..95
Chocolate Pecan Cookies..96
Berry & Dark Chocolate Cups..97
Chocolate Chip Cookies..98
Keto Cheesecake..99

***Aim for <u>at least one serving</u> of a breakfast, lunch, dinner, and side dish <u>every day</u>.**

Breakfast

Avocado Smoothie

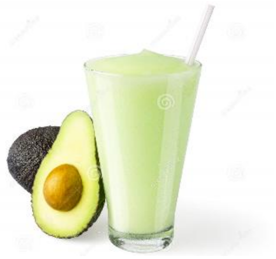

Makes 1 serve.
Preparation time: SHORT (<15 minutes).

1 avocado
1 cup canned coconut cream
1/4 cup water
1 tsp vanilla extract
2 tsp Natvia
2 ice cubes (optional)

(1) Place all ingredients in a blender and pulse until smooth.
(2) Pour into a mug, serve, and enjoy.

PER SERVE:

**Calories**	**553**
**Fat**	**52.3 g (20.3 g saturated)**
**Protein**	**6.7 g**
**Fibre**	**17.2 g**
**Net carb**	**4.6 g**

Chia Pudding

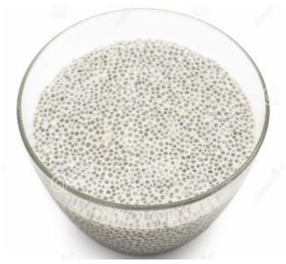

Makes 1 serve.
Preparation time: SHORT (<15 minutes).

1/2 cup any flavour Raglan/Cathedral Cove coconut yogurt
1/4 cup chia seeds
1 tsp vanilla extract
1/2 tsp ground cinnamon (optional)

(1) Place all ingredients in a bowl.
(2) Mix together and serve.

PER SERVE:

**Calories**	**525**
**Fat**	**41.0 g (24.0 g saturated)**
**Protein**	**11.6 g**
**Fibre**	**21.2 g**
**Net carb**	**10.2 g**

Berry Smoothie

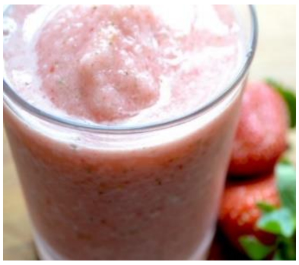

Makes 1 serve.
Preparation time: SHORT (<15 minutes).

2 strawberries OR 8 raspberries OR 8 blackberries OR 12 blueberries
1/2 cup canned coconut cream
1/3 cup water
1 tbsp vanilla extract
1 tbsp chia seeds
2 tsp Natvia
2 ice cubes (optional)

(1) Combine all ingredients in a blender and pulse until smooth.
(2) Pour into a mug, serve, and enjoy.

PER SERVE:

**Calories**	**307**
**Fat**	**27.2 g (16.4 g saturated)**
**Protein**	**5.0 g**
**Fibre**	**9.6 g**
**Net carb**	**3.0 g**

Keto Porridge

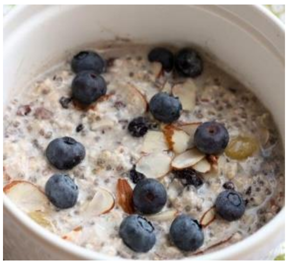

Makes 2 serves.
Preparation time: SHORT (<15 minutes).

Porridge:
1½ cups canned coconut cream
3 tbsp chia seeds
3 tbsp Ceres Organics ground flaxseed
1 tbsp sunflower seeds
1½ tbsp Natvia
1 tsp ground cinnamon
Pinch of salt

Toppings (all of the following PER SERVE):
2 strawberries OR 8 raspberries OR 8 blackberries OR 12 blueberries
1/4 cup canned coconut cream (optional)
1 tsp maple syrup (optional)

(1) Mix all porridge ingredients in a saucepan and bring to a boil on high heat.
(2) Lower the heat and simmer 1-2 minutes, until desired thickness is reached.
(3) Add the toppings and serve!

PER SERVE:

**Calories**	**440**
**Fat**	**37.3 g (17.4 g saturated)**
**Protein**	**9.5 g**
**Fibre**	**16.5 g**
**Net carb**	**4.1 g**

Bacon & Eggs

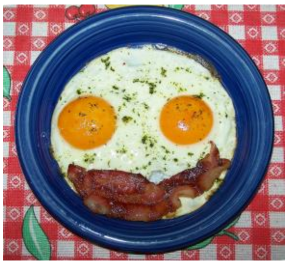

Makes 1 serve.
Preparation time: SHORT (<15 minutes).

1 tbsp cold pressed extra virgin coconut oil
2 strips streaky bacon
2 eggs
Salt and pepper to taste

(1) Heat the coconut oil in a pan over medium heat. Fry the bacon strips until they are just the way you like them and put aside on a plate; leave the oil and bacon grease in the pan.
(2) Crack the eggs into the pan and cook. If you like sunny side up, leave the eggs to fry on one side and cover the pan with a lid to make sure they get cooked on top. For eggs cooked over easy, flip the eggs over after a few minutes and cook for another minute.
(3) Add plenty of salt and pepper and serve.

PER SERVE:

**Calories**	**449**
**Fat**	**40.0 g (21.6 g saturated)**
**Protein**	**22.4 g**
**Fibre**	**0 g**
**Net carb**	**1.0 g**

Keto Pancakes

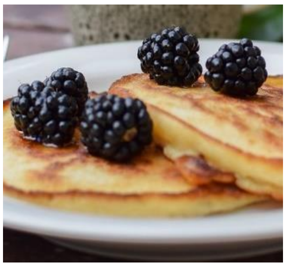

Makes 2 serves (4 pancakes) total; 1 serve = 2 pancakes.
Preparation time: SHORT (<15 minutes).

Pancake Mix:
3/4 cup almond meal
3 eggs
3 tbsp canned coconut cream
1 tsp vanilla extract
1/2 tsp baking powder
2 tbsp cold pressed extra virgin coconut oil

Toppings (all of the following PER SERVE):
2 strawberries <u>OR</u> 8 raspberries <u>OR</u> 8 blackberries <u>OR</u> 12 blueberries
1/4 cup canned coconut cream
1 tbsp unsalted butter (optional)

(1) Place the almond meal, eggs, coconut cream, vanilla, and baking powder into a large bowl and whisk together to create the pancake mix.
(2) Heat 1 tbsp coconut oil over medium-high heat; when hot, reduce to low medium and spoon 1/4 cup pancake mix onto the pan for each pancake. Cook 3-4 minutes, flip over, cook another 1-2 minutes, then transfer to a plate (after cooking 2 pancakes, add the remaining 1 tbsp coconut oil to the pan for the next 2 pancakes).
(3) Top with the coconut cream and berries. Serve and enjoy!

PER SERVE:

**Calories**	**607**
**Fat**	**54.7 g (22.1 g saturated)**
**Protein**	**21.3 g**
**Fibre**	**3.3 g**
**Net carb**	**4.7 g**

Spinach, Onion, & Goat Cheese Omelette

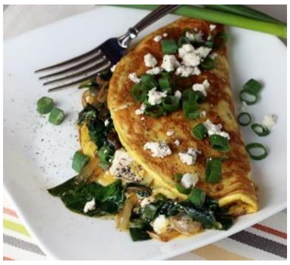

Makes 1 serve.
Preparation time: MODERATE (15-30 minutes).

2 tbsp unsalted butter
1/4 brown onion
2 stalks spring onions
60 g (1/2 bag) spinach (or kale) leaves
3 eggs
2 tbsp dairy cream
Salt and pepper to taste
30 g goat feta cheese

(1) Melt the butter in a pan over medium heat. Add the sliced onion and spring onion to the pan and saute them in the oil for 2-3 minutes.
(2) Add the spinach until it wilts; let the vegetables soak up all the butter, then transfer them to a plate. Leave the pan on the stove, but reduce heat to low-medium.
(3) Mix together the eggs, cream, and salt and pepper in a separate bowl. Pour this mixture into the pan and let the omelette cook.
(4) Once the omelette edges begin to set, spoon the vegetables over one half of the omelette, then crumble the feta cheese over the vegetables.
(5) When the top of the omelette begins to set, fold it over. Serve and enjoy!

PER SERVE:

**Calories**	**613**
**Fat**	**52.4 g (17.2 g saturated)**
**Protein**	**31.4 g**
**Fibre**	**2.3 g**
**Net carb**	**4.9 g**

Keto Granola

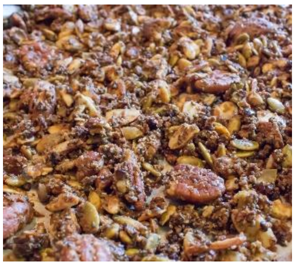

Makes 8 serves.
Preparation time: MODERATE (15-30 minutes).

Granola:
3 cups nuts (suggest 1½ cups almonds, 1½ cups walnuts)
2 tbsp sunflower seeds
2 tbsp pumpkin seeds
2 tbsp chia seeds
2 tbsp Ceres Organics ground flaxseed
1/2 cup almond meal
1 egg
1/3 cup unsalted butter, melted
1/3 cup cold pressed extra virgin coconut oil, melted
1 tbsp vanilla extract
1/4 cup Natvia

Toppings (all of the following PER SERVE):
2 strawberries OR 8 raspberries OR 8 blackberries OR 12 blueberries
1/4 cup canned coconut cream OR 3 tbsp any flavour Raglan/Cathedral Cove coconut yogurt

(1) Preheat oven to 180 C. Partially crush the walnuts and almonds in a plastic bag with a rolling pin (this is optional, you can leave them whole if you prefer).
(2) Mix all the granola ingredients into a large bowl, then smooth them into a flat even layer on a baking tray greased with coconut oil or butter. Place in the oven for 10 minutes.
(3) Pull the granola out of the oven and mix it around, making sure the edges don't burn; place in the oven another 5-10 minutes, or until the granola has turned golden brown all over.
(4) Add the toppings, serve, and enjoy!

PER SERVE:

**Calories**	**631**
**Fat**	**60.2 g (23.5 g saturated)**
**Protein**	**14.2 g**
**Fibre**	**8.0 g**
**Net carb**	**7.7 g**

Kale, Chorizo, & Eggs

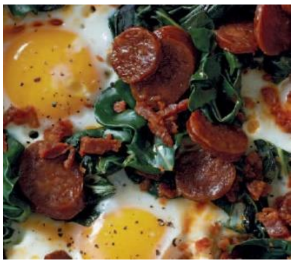

Makes 1 serve.
Preparation time: MODERATE (15-30 minutes).

1 tbsp cold pressed extra virgin coconut oil
1 tbsp butter
1 (10 cm long) chorizo sausage (or your favourite sausage)
60 g (1/2 bag) kale leaves
2 eggs
Salt and pepper to taste

(1) Heat the coconut oil and butter in a pan over medium heat. Gently fry the sliced chorizo until golden brown, then remove to a plate but leave the oil and butter in the pan.
(2) Fry the kale in the pan until it begins to wilt. When it does, shape the kale into a nest and crack the eggs on top of it.
(3) Cook 3 minutes or until the eggs are opaque. Slide the kale nest (plus eggs) onto a second plate, then add the chorizo over top.
(4) Pour the oil and butter over top, add plenty of salt and pepper, and serve.

PER SERVE:

**Calories**	**665**
**Fat**	**58.9 g (31.5 g saturated)**
**Protein**	**30.4 g**
**Fibre**	**1.4 g**
**Net carb**	**2.7 g**

Bacon Avocado Muffins

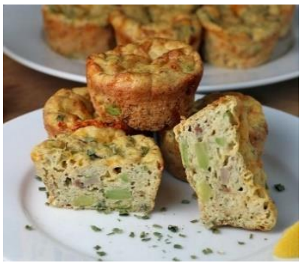

Makes 6 serves (12 muffins) total; 1 serve = 2 muffins.
Preparation time: LONG (30-45 minutes).

1/2 cup almond meal
1/4 cup Ceres Organics ground flaxseed
1½ tbsp psyllium husk
1½ cups canned coconut cream
Juice from 1 lemon
2 tbsp unsalted butter
5 strips streaky bacon
2 avocados
30 g cheddar cheese
3 stalks spring onion
5 eggs
1 clove garlic (or 1 tsp crushed garlic)
1 tbsp coriander
1 tsp baking powder
Salt and pepper to taste

(1) Preheat the oven to 180 C. Mix together the almond meal, flaxseed, psyllium husk, coconut cream, and lemon juice in a large bowl. Set aside.
(2) Add the butter to a pan and cook the sliced bacon over medium heat to your liking. Add the bacon to the mixture in the bowl.
(3) Slice/grate the avocados, cheese, and spring onions, then add them plus the eggs, garlic, coriander, and baking powder to the mixture in the bowl to create the batter.
(4) Measure the batter between 12 greased muffin cups and bake 20-30 minutes; after 20 minutes, check every 2-3 minutes; remove when the tops of the muffins are brown. Serve!

PER SERVE:

**Calories**	**474**
**Fat**	**40.7 g (16.7 g saturated)**
**Protein**	**16.3 g**
**Fibre**	**6.9 g**
**Net carb**	**4.2 g**

Vanilla Pecan Bars

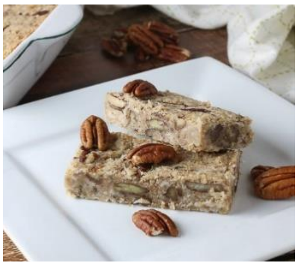

Makes 4 serves (4 bars) total; 1 serve = 1 bar.
Preparation time: VERY LONG (>45 minutes; but nearly all oven and fridge time).

Dry Ingredients:
1 cup pecan (or walnut) halves
1/2 cup almond meal
1/4 cup Ceres Organics ground flaxseed
2 tbsp Natvia

Wet Ingredients:
1/4 cup cold pressed extra virgin coconut oil, melted
1 tbsp unsalted butter, melted
2 tbsp vanilla extract

(1) Preheat the oven to 180 C. Place the pecans in a small plastic bag and crush with a rolling pin.
(2) Add the pecans and rest of the dry ingredients to a large bowl and mix together.
(3) Add the coconut oil, butter, and vanilla to the dry ingredients; mix into a crumbly dough.
(4) Press the dough into a small baking dish and bake for 15-20 minutes.
(5) Remove the dish from the oven and let it cool for at least 15 minutes, then place it in the fridge for at least 1 hour (or overnight) to solidify. Cut into four equal-sized bars and enjoy.

PER SERVE:

**Calories**	**467**
**Fat**	**46.7 g (16.4 g saturated)**
**Protein**	**8.2 g**
**Fibre**	**5.5 g**
**Net carb**	**3.2 g**

Lunch
(Salads)

Raspberry Arugula Salad

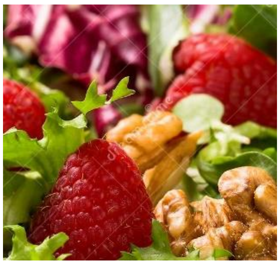

Makes 3 serves.
Preparation time: SHORT (<15 minutes).

Salad:
60 g (1/2 bag) arugula (or any other type of lettuce)
24 raspberries (or blueberries)
1/2 cup pine nuts (or cashews, almonds, walnuts)

Dressing:
1/2 lemon
1/3 cup extra virgin olive oil
Salt and pepper to taste

(1) Add the arugula, berries, and nuts to a large bowl.
(2) Squeeze the lemon juice over top, followed by the oil, then add salt and pepper. Serve!

PER SERVE:

**Calories**	**362**
**Fat**	**37.9 g (4.3 g saturated)**
**Protein**	**3.9 g**
**Fibre**	**2.3 g**
**Net carb**	**3.2 g**

Strawberry Kale Salad

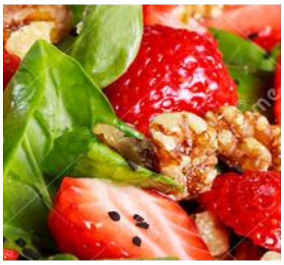

Makes 3 serves.
Preparation time: SHORT (<15 minutes).

Salad:
60 g (1/2 bag) kale leaves
8 strawberries (or 24 raspberries)
1 cup walnuts (or almonds)

Dressing:
1/3 cup extra virgin olive oil
1 tbsp balsamic vinegar
Salt and pepper to taste

(1) Add the kale leaves, sliced berries, and nuts to a large bowl.
(2) Pour the oil and vinegar over top, then add salt and pepper. Serve!

PER SERVE:

**Calories**	**431**
**Fat**	**44.4 g (5.2 g saturated)**
**Protein**	**5.8 g**
**Fibre**	**3.4 g**
**Net carb**	**4.4 g**

Greek Salad

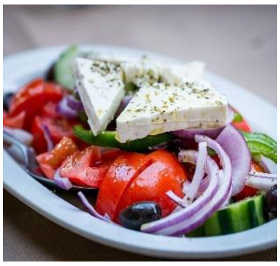

Makes 3 serves.
Preparation time: SHORT (<15 minutes).

Salad:
2 tomatoes
2 Lebanese (small) cucumbers
1/2 red onion
1/2 capsicum
1 avocado
150 g whole pitted olives
150 g goat feta cheese
1 tsp oregano
1 tsp salt

Dressing:
1/2 cup extra virgin olive oil
2-4 tbsp balsamic vinegar

(1) Combine tomatoes, cucumbers, onion, capsicum, avocado, olives, and feta in a salad bowl. Sprinkle over the oregano and salt.
(2) Pour the oil and vinegar over the salad and toss to combine. Serve (eat all the oil)!

PER SERVE:

**Calories**	**554**
**Fat**	**52.0 g (16.2 g saturated)**
**Protein**	**12.9 g**
**Fibre**	**8.2 g**
**Net carb**	**9.6 g**

Caprese Salad

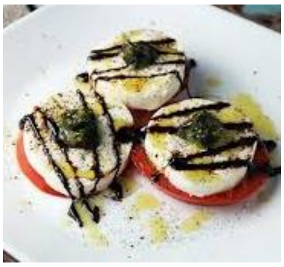

Makes 2 serves.
Preparation time: SHORT (<15 minutes).

4 tbsp fresh basil, chopped
4 tbsp extra virgin olive oil
1 tomato
125 g ball Massimo's mozzarella cheese
Balsamic vinegar to taste
Salt and pepper to taste

(1) Add the basil leaves and one tablespoon of the olive oil to a blender. Pulse until you have a basil paste. Set aside.
(2) Slice the tomato half into three thick slices, then do the same to the mozzarella half. Arrange them on a plate by placing each mozzarella slice on top of a tomato slice.
(3) When ready to eat, pour the basil paste over the top of the tomato and mozzarella, then pour the extra olive oil on top, dribble over the Balsamic vinegar, season with salt and pepper, and serve!

PER SERVE:

**Calories**	**440**
**Fat**	**39.6 g (10.9 g saturated)**
**Protein**	**16.9 g**
**Fibre**	**0.8 g**
**Net carb**	**4.1 g**

Mediterranean Salad

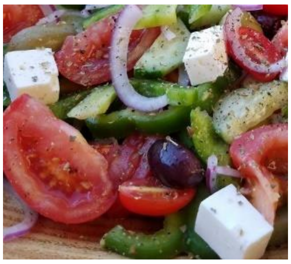

Makes 3 serves.
Preparation time: SHORT (<15 minutes).

Salad:
3 Lebanese (small) cucumbers
10 cherry tomatoes (or 2 tomatoes)
1/4 red onion
1/2 capsicum
2/3 cup almonds
75 g whole pitted olives
75 g goat feta cheese
3 tbsp fresh basil (or parsley), chopped

Dressing:
Juice from 1/2 lemon
1/3 cup extra virgin olive oil
2-3 tbsp balsamic vinegar
Salt and pepper to taste

(1) Slice and add the cucumbers, tomatoes, onion, capsicum, olives, almonds (whole or slivered), feta, and basil to a large bowl.
(2) Pour the lemon juice, oil, vinegar, and plenty of salt and pepper over the salad and toss to combine. Serve (eat all the oil).

PER SERVE:

**Calories**	**544**
**Fat**	**51.6 g (10.4 g saturated)**
**Protein**	**13.1 g**
**Fibre**	**6.4 g**
**Net carb**	**11.0 g**

Bacon, Broccoli, & Almond Salad

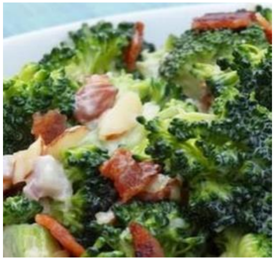

Makes 3 serves.
Preparation time: MODERATE (15-30 minutes).

1 tbsp cold pressed extra virgin coconut oil
6 strips streaky bacon
2 tbsp extra virgin olive oil
1/4 cup Real Mayonnaise (or full-fat sour cream)
1 tbsp apple cider vinegar
Juice from 1/2 lemon
Salt and pepper to taste
1 head broccoli
1/4 red onion
1/4 cup almonds (or pine nuts)

(1) Heat the coconut oil in a pan over medium heat. Fry the bacon strips until they are just the way you like them.
(2) While the bacon cooks, combine the olive oil, mayonnaise, vinegar, lemon juice, and salt and pepper in a small bowl.
(3) Finely slice the broccoli, onion, and almonds (or leave the almonds whole) and add to a large bowl. Then add the sliced bacon (include all the oil it cooked in) to the large bowl.
(4) Pour the dressing in the small bowl over top. Mix everything together and serve!

PER SERVE:

**Calories**	**506**
**Fat**	**48.5 g (13.7 g saturated)**
**Protein**	**13.0 g**
**Fibre**	**3.5 g**
**Net carb**	**4.8 g**

Salmon Salad

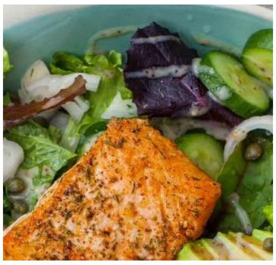

Makes 3 serves.
Preparation time: MODERATE (15-30 minutes).

Dressing:
Juice from 1/2 lemon
1/3 cup extra virgin olive oil
8 fresh basil leaves

Salad:
60 g (1/2) salad mix
1 Lebanese (small) cucumber
1 avocado
1/4 red onion
60 g goat feta cheese
1/4 cup walnut halves (or almonds)
1 tbsp cold pressed extra virgin coconut oil
360 g salmon fillets
Salt and pepper to taste

(1) Blend the dressing ingredients in a blender. Set aside.
(2) Add the salad to a large salad bowl, then mix in the sliced cucumber, avocado, onion, feta, and walnuts. Mix in the dressing.
(3) Heat the coconut oil in a pan over medium heat while you season the salmon fillets with salt and pepper.
(4) Cook the fillets for 3-4 minutes on one side. Flip them over and cook the other side until the middle is pink (do not overcook).
(5) Cube the salmon fillets and add them, plus all the oil, to the salad base. Mix the salad well, add more salt and pepper, and serve.

PER SERVE:

**Calories**	**695**
**Fat**	**60.5 g (15.9 g saturated)**
**Protein**	**31.4 g**
**Fibre**	**5.9 g**
**Net carb**	**4.4 g**

Basil Pesto Chicken Salad

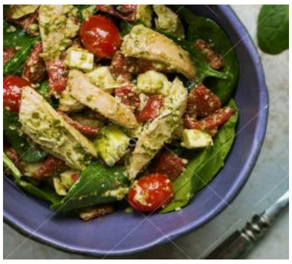

Makes 3 serves.
Preparation time: MODERATE (15-30 minutes).

Marinade:
1/3 cup basil pesto
1/3 cup extra virgin olive oil
1/4 cup balsamic vinegar

Salad:
400 g chicken thighs
60 g (1/2 bag) salad mix
60 g mozzarella (or your favourite) cheese, grated
1 avocado
2 tomatoes
2 tbsp fresh basil, chopped
Salt and pepper to taste

(1) To make the marinade, mix the pesto, oil, and vinegar in a large bowl. Put half the marinade into a smaller bowl, so you have two bowls of marinade.
(2) Place the chicken thighs in the large bowl of marinade, turn to fully coat them, then place in the fridge for 10-15 minutes. Set the smaller bowl of marinade aside for now.
(3) While the chicken is in the fridge, put the salad mix into another large bowl, followed by the grated cheese, sliced avocado and tomatoes, and basil.
(4) When the fridge time is done, set a large pan over medium heat. Place the chicken thighs and its marinade into the pan and cook for 6-7 minutes on each side of the thighs.
(5) Slice the chicken and place on the salad. Pour the smaller bowl of marinade over top, season with salt and pepper, and serve.

PER SERVE:

**Calories**	**706**
**Fat**	**57.3 g (10.6 g saturated)**
**Protein**	**35.4 g**
**Fibre**	**6.9 g**
**Net carb**	**6.9 g**

Lunch
(Stews & Soups)

Crockpot Chicken Stew

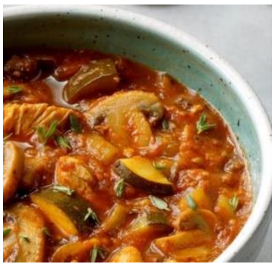

Makes 4 serves.
Preparation time (excluding crockpot time): SHORT (<15 minutes).

Crockpot:
600 g chicken thighs
2 tsp Moroccan (or your favourite) seasoning
1 tsp cumin seeds
Salt and pepper to taste
1 green capsicum
6-8 mushrooms (depends on size)
400 g (1 can) canned tomatoes, whole with juice
1/4 cup unsalted butter
3 tbsp cold pressed extra virgin coconut oil

Toppings (any or all of the following PER SERVE):
1/4 avocado
1-2 tbsp full- fat sour cream, room temperature
1 tbsp fresh coriander, chopped

(1) Slice the chicken into 1-inch chunks. Place ingredients (in order listed) in the crockpot.
(2) Mix the ingredients together set the crockpot on high, and cook for 6-7 hours.
(3) Once cooked, place in a bowl, add toppings and more seasoning, and enjoy!

PER SERVE:

**Calories**	**505**
**Fat**	**37.7 g (20.4 g saturated)**
**Protein**	**32.7 g**
**Fibre**	**5.7 g**
**Net carb**	**3.8 g**

Crockpot Steak Stew

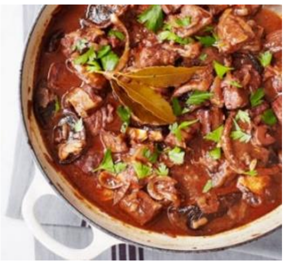

Makes 4 serves.
Preparation time (excluding crockpot time): SHORT (<15 minutes).

Crockpot:
600 g scotch fillet steak
2 tsp steak or grill seasoning
1 tsp cumin seeds
Salt and pepper to taste
1 green capsicum
3-4 mushrooms (depends on size)
6 stalks spring onions, sliced
400 g (1 can) canned tomatoes, whole with juice
1/2 cup beef stock
3 tbsp cold pressed extra virgin coconut oil

Toppings (any or all of the following PER SERVE):
1/4 avocado
1-2 tbsp full-fat sour cream, room temperature
1 tbsp fresh coriander, chopped

(1) Slice the steak into 1-inch chunks. Place ingredients (in order listed) in the crockpot.
(2) Mix the ingredients together, set the crockpot on high, and cook for 6 hours.
(3) Once cooked, place in a bowl, add toppings and more seasoning, and enjoy!

PER SERVE:

**Calories**	**595**
**Fat**	**43.9 g (20.0 g saturated)**
**Protein**	**40.8 g**
**Fibre**	**5.6 g**
**Net carb**	**6.5 g**

Keto Hamburger Soup

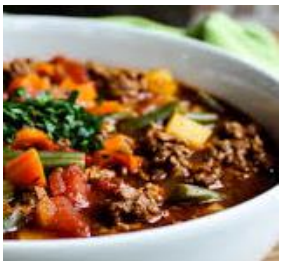

Makes 3 serves.
Preparation time: MODERATE (15-30 minutes).

3 tbsp cold pressed extra virgin coconut oil
450 g regular beef mince
1 carrot
4 stalks celery
1 courgette
1/4 brown onion
2 cups beef stock
1 tomato
10 fresh basil leaves
Salt and pepper to taste

(1) Heat the oil in a large pot over medium heat. Saute the mince until completely browned.
(2) Slice and mix in the carrot, celery, courgette, and onion. Brown the vegetables slightly.
(3) Pour in the stock, followed by the tomato. Bring to a boil, then turn down the heat to maintain a strong simmer for 10-15 minutes, stirring occasionally.
(4) Season with the basil leaves followed by lots of salt and pepper, then serve.

PER SERVE:

**Calories**	**445**
**Fat**	**34.0 g (19.6 g saturated)**
**Protein**	**28.2 g**
**Fibre**	**1.9 g**
**Net carb**	**5.9 g**

Chicken Enchilada Soup

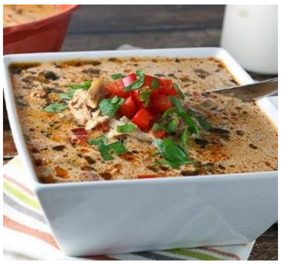

Makes 3 serves.
Preparation time: LONG (30-45 minutes).

2 tbsp cold pressed extra virgin coconut oil
3 stalks celery
1 capsicum
1 tomato
2 cloves garlic (or 2 tsp crushed garlic)
2 tsp (each) of cumin seeds and oregano
3 cups chicken stock
1/2 cup fresh coriander, chopped
90 g cream cheese
1/2 roast chicken (eat all the skin, and remove all stuffing)
Juice from 1 lime

(1) Heat the coconut oil in a large pan over medium heat, then add the sliced celery and capsicum. Once the celery is cooked, mix in sliced tomato, garlic, and spices and cook another 2-3 minutes.
(2) Transfer the mixture to a large pot. Pour in the stock, then add the coriander. Bring to a boil, then reduce to low heat and simmer for 10-15 minutes.
(3) Add the cream cheese and simmer another 10-15 minutes.
(4) Shred the chicken with a fork and add to the pot. Juice the lime over top and serve.

PER SERVE:

**Calories**	**467**
**Fat**	**32.0 g (11.4 g saturated)**
**Protein**	**34.7 g**
**Fibre**	**1.4 g**
**Net carb**	**7.7 g**

Thai Coconut Chicken Soup

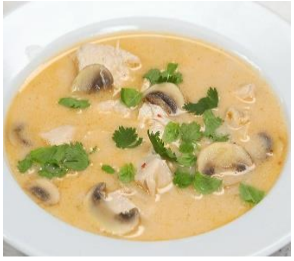

Makes 2 serves.
Preparation time: LONG (30-45 minutes).

4 cups chicken stock
2 tbsp cold pressed extra virgin coconut oil
2 stalks spring onions
Juice from 1 lime
1 inch ginger (optional)
1/2 tsp salt
400 g chicken thighs
8-12 mushrooms (depends on size)
1½ cups canned coconut cream
3 tbsp fresh coriander, chopped

(1) Heat the stock and coconut oil in a large pot over medium-high heat. Add the sliced spring onions as well as the lime juice, ginger, and salt. Reduce the heat and simmer 5-10 minutes.
(2) Add the whole chicken thighs and sliced mushrooms. Simmer 15-20 minutes.
(3) Remove the chicken thighs and shred them, then add them back in along with the coconut cream. Simmer 5 minutes; taste and add more salt if needed.
(4) Garnish with the coriander and serve.

PER SERVE:

**Calories**	**585**
**Fat**	**38.8 g (25.7 g saturated)**
**Protein**	**47.5 g**
**Fibre**	**3.1 g**
**Net carb**	**8.4 g**

Lunch
(Sandwiches & Breads)

Keto Cheeseburgers

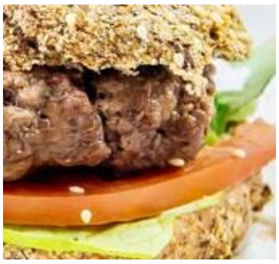

Makes 4 serves (4 burgers) total; 1 serve = 1 burger.
Preparation time: MODERATE (15-30 minutes, unless you also make Keto Bread).

Lettuce buns (4-8 lettuce leaves) <u>OR</u> any Keto Bread recipe (8 buns or slices)
1/2 cup full-fat sour cream (or Real Mayonnaise)
1 tbsp Worcester sauce
400 g regular beef mince
2 tsp steak or grill seasoning
1/2 tbsp apple cider vinegar
Salt and pepper to taste
2 tbsp cold pressed extra virgin coconut oil
4-8 slices mozzarella (or your favourite) cheese
1 tomato
4 more lettuce leaves (if using bread buns)

(1) Mix together the sour cream and Worcester sauce in a bowl. Set aside.
(2) Use your hands to mix together the mince, seasoning, vinegar, salt, and pepper in a bowl. Create four equal-sized patties (1-2 cm thick). Pierce each patty several times with a fork.
(3) Heat the coconut oil in a large pan over medium-high heat. Place the patties in the sizzling oil. Cook 4-5 minutes, then flip them and cook another 3-4 minutes; make sure the patties are fully cooked on the inside.
(4) When done, transfer each patty to a leaf (or bun), and quickly top with 1 or 2 cheese slices so the cheese melts. Add sliced tomato, lettuce, and mayo Worcester sauce followed by more seasoning, and close with the remaining leaf (or bun) to make four burgers. Serve!

PER SERVE:

**Calories**	**534**
**Fat**	**47.8 g (19.1 g saturated)**
**Protein**	**23.6 g**
**Fibre**	**0.6 g**
**Net carb**	**1.1 g**

Chicken Avocado Sandwiches

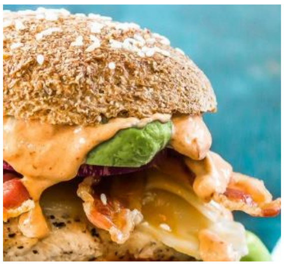

Makes 4 serves (4 big sandwiches) total; 1 serve = 1 sandwich.
Preparation time: MODERATE (15-30 minutes, unless you also make Keto Bread).

Lettuce buns (4-8 lettuce leaves) OR any Keto Bread recipe (8 buns or slices)
1/2 cup full-fat sour cream (or Real Mayonnaise)
1 tbsp Worcester sauce
2 tbsp cold pressed extra virgin coconut oil
400 g (four) chicken thighs
4 strips streaky bacon (or ham)
Salt, pepper, and steak or grill seasoning to taste
4 slices mozzarella (or your favourite) cheese
1 avocado

(1) Mix together the sour cream and Worcester sauce in a bowl. Set aside.
(2) Heat the coconut oil in a pan over medium heat, then fry the chicken thighs (leave them whole, do not slice) and bacon strips, adding liberal amounts of salt, pepper, and seasoning.
(3) When done, transfer one chicken thigh to each leaf (or bun) and quickly top with a cheese slice so the cheese melts, followed by the bacon, sliced avocado, mayo Worcester sauce, and more seasoning, and close with the remaining leaf (or bun) to make four sandwiches. Serve!

PER SERVE:

**Calories**	**596**
**Fat**	**51.8 g (18.3 g saturated)**
**Protein**	**29.5 g**
**Fibre**	**3.4 g**
**Net carb**	**1.0 g**

Light Keto Bread

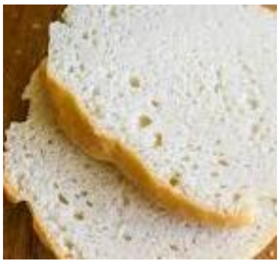

Makes 12 serves (12 slices) total; 1 serve = 1 slice.
Preparation time: LONG (30-45 minutes; but nearly all oven time).

6 eggs
1/4 tsp cream of tartar
1½ cups almond meal
1/4 cup unsalted butter, melted
1 tbsp baking powder
1 pinch salt
1/2 tsp Natvia

(1) Preheat oven to 190 C. Separate the egg whites from the yolks.
(2) Put the egg whites into a bowl, add the cream of tartar, and beat until soft peaks are achieved.
(3) Put the yolks into a blender, then add the almond meal, butter, baking powder, salt, Natvia, and a third of the beaten egg whites. Blend until combined.
(4) Add the remaining egg whites and blend until incorporated; do not blend too much though, the egg whites are what give the bread its volume.
(5) Pour mixture into a buttered loaf pan. Bake 30 minutes. Serve with butter.

PER SERVE:

**Calories**	**169**
**Fat**	**14.9 g (4.0 g saturated)**
**Protein**	**6.8 g**
**Fibre**	**0.4 g**
**Net carb**	**1.1 g**

Sarona’s Keto Bread

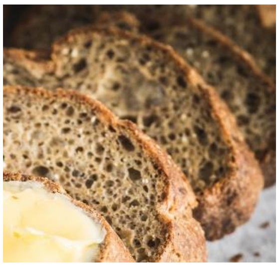

Makes 8 serves (8 buns or muffins) total; 1 serve = 1 bun or muffin.
Preparation time: VERY LONG (>45 minutes; but nearly all oven time).

Dry Ingredients:
1 cup Ceres Organics ground flaxseed
1/4 cup almond meal
5 tbsp psyllium husk
1/2 tbsp yeast
2 tsp baking powder
1/2 tsp salt

Wet Ingredients:
3 egg whites
1/2 tbsp apple cider vinegar
1 cup boiling water

(1) Preheat oven to 175 C. Mix the dry ingredients together in a large bowl, then add the egg whites and apple cider vinegar and stir together.
(2) Add the boiling water and stir until the mixture resembles a dough.
(3) If you are making the bread as part of a sandwich recipe, spoon out and shape the dough into eight equal-sized buns (diameter 10 cm, height 1 cm) on a baking tray greased with coconut oil or butter. Take your time; do this right. Alternatively, if you will eat the bread on its own, this recipe also fits nicely into eight muffin cups.
(4) Bake for 45 minutes. Serve warm with butter and enjoy!

PER SERVE:

**Calories**	**135**
**Fat**	**10.0 g (0.9 g saturated)**
**Protein**	**5.6 g**
**Fibre**	**7.6 g**
**Net carb**	**0.7 g**

Keto Nutloaf

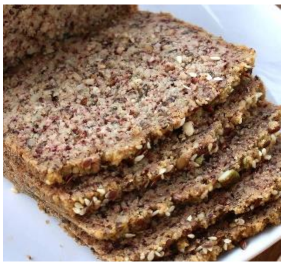

Makes 12 serves (12 slices) total; 1 serve = 1 slice.
Preparation time: VERY LONG (>45 minutes; but nearly all oven time).

Dry Ingredients:
1 cup almonds
1 cup Ceres Organics ground flaxseed
1/2 cup cashews (or peanuts)
1/2 cup pumpkin seeds
1 cup sunflower seeds
1/4 cup walnuts
1/2 tbsp baking powder
1 tsp salt

Bind Ingredients:
1/2 cup chia seeds
3 cups warm water

(1) Preheat the oven to 180 C. Combine the dry ingredients together in a large bowl (however, set aside 1/2 cup of the sunflower seeds for later).
(2) Blend the nuts mixture in a processor or blender until they are ground; you may need to do this in stages rather than all at once. Once done, place the ground mixture back in the large bowl with the whole sunflower seeds that you set aside.
(3) In a separate bowl, mix the bind ingredients into a paste. Add the paste to the large bowl with the dry ingredients and mix together (add more water if required).
(4) Bake for 45 minutes in a buttered loaf pan. Serve warm with butter and enjoy!

PER SERVE:

**Calories**	**324**
**Fat**	**26.6 g (3.0 g saturated)**
**Protein**	**11.5 g**
**Fibre**	**10.3 g**
**Net carb**	**4.9 g**

Dinner (European)

Keto Pizza

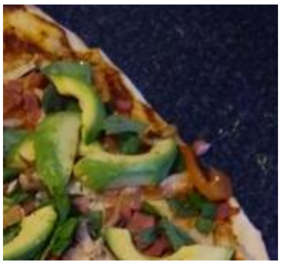

Makes 4 serves.
Preparation time: MODERATE (15-30 minutes).

Pizza Base:
6 eggs
1/4 cup coconut flour
1/4 cup psyllium husk
1 tbsp Italian seasoning
Salt and pepper to taste

Toppings:
1/4 cup pizza sauce
1/4 cup full-fat sour cream
6 slices Italian salami
3 mushrooms
1 tomato
1/2 capsicum
1 avocado
100 g cheddar cheese

(1) Preheat the oven to 200 C. Combine all pizza base ingredients in a blender; blend well.
(2) Drizzle olive oil over a circle (or rectangular) baking tray and place the pizza base on top; it will be a bit runny, that's ok just shape it into a circle (or square) as best you can (1/2 to 1 cm thick). Place in the oven for 10 minutes.
(3) Mix the pizza sauce and sour cream together in a small bowl, slice the vegetable toppings, and grate the cheese while you wait for the pizza base to bake.
(4) Remove the pizza base, flip it over, and top with the tomato paste and sour cream mixture, followed by the rest of the toppings in the order shown above.
(5) Place in the oven 4-5 more minutes until cheese is melted, and serve!

PER SERVE:

**Calories**	**508**
**Fat**	**33.4 g (15.0 g saturated)**
**Protein**	**25.8 g**
**Fibre**	**18.5 g**
**Net carb**	**7.8 g**

Caprese Chicken

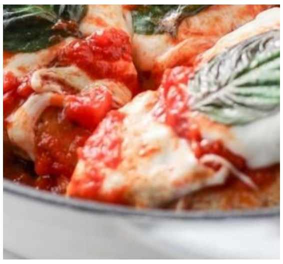

Makes 2 serves.
Preparation time: MODERATE (15-30 minutes).

1/4 cup extra virgin olive oil
400 g chicken thighs
Salt and pepper to taste
1/4 cup balsamic vinegar
2 cloves garlic (or 2 tsp crushed garlic)
10 cherry tomatoes (or 2 tomatoes)
2 tbsp freshly chopped basil
4 slices mozzarella cheese

(1) Heat the olive oil in a large pan over medium-high heat. Season chicken with salt and pepper and cook through, about 6 minutes per side. Transfer to a plate.
(2) Add balsamic vinegar to the pan, then cook minced garlic 1 minute. Add sliced tomatoes and season with salt. Simmer another 6 minutes then stir in the basil.
(3) Return the chicken thighs and nestle them amongst the tomatoes. Top each thigh with a slice of mozzarella, then cover the pan with a lid for 1-2 minutes so the cheese melts.
(4) Spoon the tomatoes over the chicken and serve (eat all the oil and vinegar)!

PER SERVE:

**Calories**	**634**
**Fat**	**46.8 g (14.3 g saturated)**
**Protein**	**48.5 g**
**Fibre**	**0 g**
**Net carb**	**2.2 g**

Tuscan Salmon

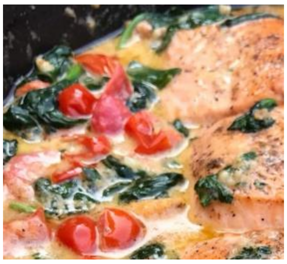

Makes 3 serves.
Preparation time: MODERATE (15-30 minutes).

3 tbsp cold pressed extra virgin coconut oil
400 g salmon (or whitefish) fillets
Salt and pepper to taste
3 tbsp unsalted butter
3 cloves garlic (or 3 tsp crushed garlic)
2 tomatoes (or 10 cherry tomatoes)
60 g (1/2 bag) spinach (or kale) leaves
1/2 cup canned coconut cream
50 g parmesan cheese
1/4 cup fresh herbs (basil or parsley), chopped
Juice from 1 lemon

(1) Heat the coconut oil in a pan over medium heat. Season salmon fillets with salt and pepper.
(2) Cook the salmon fillets 3-4 minutes on one side. Flip them over and cook the other side until the middle is pink (do not overcook). Transfer the fillets to a plate, but leave the oil in the pan.
(3) Add the butter to the pan. When melted, stir in the minced garlic and cook for 1 minute, then add the sliced tomatoes, season with more salt and pepper, and cook another 3-4 minutes. Add the spinach and cook until it begins to wilt (just begins to wilt, not fully wilted).
(4) Stir in the coconut cream, sliced cheese, and herbs. Reduce heat to low and simmer 3 minutes, until the sauce is slightly reduced (mix to prevent burning).
(5) Return the salmon to the pan and mix it in with the sauce; cook another 3 minutes. Squeeze lemon juice over top before serving. Enjoy!

PER SERVE:

Calories	601
Fat	48.7 g (27.9 g saturated)
Protein	35.6 g
Fibre	2.1 g
Net carb	3.3 g

Lemony Gurnard

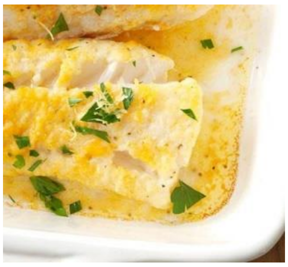

Makes 2 serves.
Preparation time: MODERATE (15-30 minutes).

1/4 cup almond meal
1/2 tbsp (each) of dill and chives
1 tsp onion powder
1 tsp garlic powder
Salt and pepper to taste
400 grams fresh Gurnard (or any whitefish) fillets
3 tbsp cold pressed extra virgin coconut oil
3 tbsp unsalted butter
Juice from 2 lemons

(1) Mix together the almond meal, all spices, and salt and pepper in a large bowl.
(2) Take the fish fillets, one at a time, and press into the flour mix. Turn and repeat. You want to really cover them well and place on a separate plate once done. Use all the flour mix!
(3) In a large pan, heat the coconut oil, butter, and lemon juice over medium-high heat. You want it hot enough to crust the flour mix but not so hot to burn the butter.
(4) Fry the fish fillets 4-5 minutes, flip, and fry another 2-3 minutes. Wriggle the pan frequently so the fillets soak up all the fats and juice. Don't let your pan dry out; add more coconut oil or butter if necessary. The fillet coating should be golden brown when done.
(5) Check if the fish fillets are done with a fork and remove, including all the sauce, from the pan when almost done; do not overcook. Serve (eat all the oil and butter)!

PER SERVE:

**Calories**	**649**
**Fat**	**56.1 g (31.7 g saturated)**
**Protein**	**37.5 g**
**Fibre**	**0.5 g**
**Net carb**	**0.9 g**

Bacon & Basil Pesto Mushroom Cups

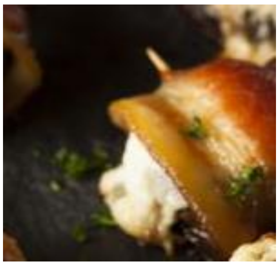

Makes 3 serves (12 mushroom cups) total; 1 serve = 4 mushroom cups.
Preparation time: MODERATE (15-30 minutes).

75 grams cream cheese
2 tbsp basil pesto
12 mushrooms
12 strips streaky bacon

(1) Preheat oven to 175 C. Combine the cream cheese with the pesto in a bowl.
(2) Gently remove the stalk from each mushroom. Where the stalk has been removed place a spoonful of the cheese and pesto mix into the mushroom cup. Repeat this process until all mushrooms have been filled; use all the mix (it's ok if there's not enough room in the mushroom cup, pile the mix on top).
(3) Take a strip of bacon and wrap it around one of the mushrooms (top to bottom, not around the bell) so the mushroom is nicely covered. Repeat for all mushrooms.
(4) Place in an oven dish and bake until the bacon looks golden brown and slightly crispy. This should take around 15-20 minutes depending on the size of your mushrooms (small mushrooms need less time, so keep an eye on them to ensure they don't burn). Enjoy.

PER SERVE:

**Calories**	**476**
**Fat**	**42.8 g (18 g saturated)**
**Protein**	**19.6 g**
**Fibre**	**0.4 g**
**Net carb**	**2.4 g**

Seasoned Salmon Fry

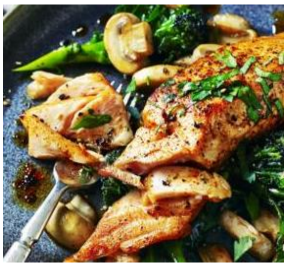

Makes 2 serves.
Preparation time: MODERATE (15-30 minutes).

3 mushrooms
1/2 capsicum
2 stalks spring onions
3 tbsp cold pressed extra virgin coconut oil
240 g salmon (or whitefish) fillets
1 tsp (each) of oregano and cumin seeds
Salt and pepper to taste
Juice of 1/2 lemon
2 tbsp pumpkin seeds
60 g (1/2 bag) spinach (or kale) leaves

(1) Slice the mushrooms, capsicum, and spring onions, then set aside.
(2) Heat 1 tbsp coconut oil in a pan over medium-high heat. Generously season the salmon fillets with seasonings, salt, and pepper. Cook the fillets for 3-4 minutes on one side, flip them over, and cook the other side until the middle is pink (do not overcook).
(3) Drip lemon juice over the fillets and transfer them to a plate, but leave the oil in the pan. Place another plate on top of the fillets to keep them warm while you make the vegetables.
(4) Turn heat to high and add the remaining 2 tbsp coconut oil. When melted, add the mushrooms, capsicum, spring onion, and pumpkin seeds and cook for 2-3 minutes. Turn off the heat and add the spinach leaves; after 2-3 minutes, stir in the leaves (they should be slightly wilted).
(5) Sprinkle salt and pepper over the vegetables and transfer them (plus all the oil) to the plate with the fillets. Serve and enjoy!

PER SERVE:

**Calories**	**460**
**Fat**	**37.6 g (21.3 g saturated)**
**Protein**	**27.5 g**
**Fibre**	**1.8 g**
**Net carb**	**3.0 g**

Tarragon Dill Salmon

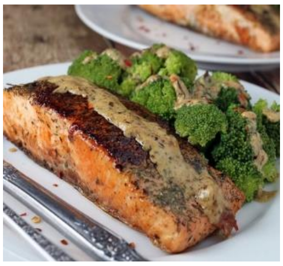

Makes 2 serves.
Preparation time: MODERATE (15-30 minutes).

240 g salmon (or whitefish) fillets
2 tsp (each) of tarragon and dill weed
Salt and pepper to taste
2 tbsp cold pressed extra virgin coconut oil
2 tbsp unsalted butter
1/4 cup canned coconut cream

(1) Season the salmon fillets with half the tarragon and half the dill weed, plus salt and pepper.
(2) Heat the coconut oil in a pan over medium-high heat. Meanwhile, season the salmon fillets with seasonings, salt, and pepper.
(3) Cook the fillets for 3-4 minutes on one side. Flip them over and cook the other side until the middle is pink (do not overcook). When done, remove the fillets to a plate. Set aside.
(3) Add the butter to the pan. Once it has melted, throw in the rest of the tarragon, dill weed, and plenty of salt and pepper, then add the cream. Mix together to create the tarragon dill cream sauce and pour it all over the salmon fillets. Serve and enjoy (eat all the sauce)!

PER SERVE:

**Calories**	**467**
**Fat**	**41.1 g (23.8 g saturated)**
**Protein**	**24.4 g**
**Fibre**	**0.5 g**
**Net carb**	**0.1 g**

Chicken Bacon Drumsticks

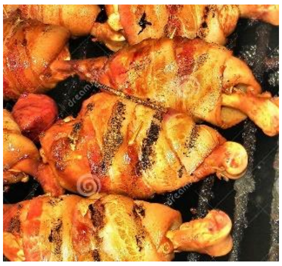

Makes 2 serves.
Preparation time: LONG (30-45 minutes; but nearly all oven time).

4 chicken drumsticks (skin on)
4 strips streaky bacon
Salt and pepper to taste

(1) Preheat oven to 200 C. Line a baking tray with aluminium foil.
(2) Wrap one slice of bacon around each drumstick, working from the bottom of the drumstick to the top (if you really like bacon, you can wrap two slices around each drumstick). Place on the baking tray and season with salt and pepper.
(3) Bake 45 minutes, season with more salt and pepper, and serve!

PER SERVE:

**Calories**	**637**
**Fat**	**39.6 g (12.7 g saturated)**
**Protein**	**66.8 g**
**Fibre**	**0 g**
**Net carb**	**0.2 g**

Greek Chicken

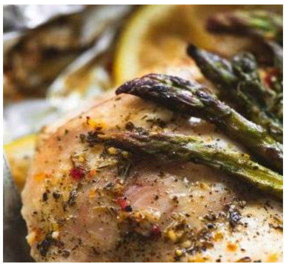

Makes 2 serves.
Preparation time: LONG (30-45 minutes; but nearly all fridge and oven time).

3 tbsp extra virgin olive oil
1 lemon
3 cloves garlic (or 3 tsp crushed garlic)
1-2 tsp oregano
3 tbsp cold pressed extra virgin coconut oil
400 g chicken thighs
Salt and pepper to taste
1 bunch of asparagus
1 courgette

(1) Preheat the oven to 200 C. In a large bowl, combine olive oil, juice from half the lemon, garlic, and oregano; whisk to combine. Add the chicken thighs, turn to fully coat them, and marinate in the fridge for 10-15 minutes.
(2) Heat the coconut oil in a large oven-proof pan over medium-high heat. Remove the chicken thighs from the fridge, season with salt and pepper, then add the chicken (plus marinade) to the pan. Cook for 10 minutes.
(3) Flip chicken over and add the sliced asparagus, courgette, and juice from the remaining lemon half. Cook another 2-3 minutes.
(4) Transfer to the oven and bake until the chicken is cooked through and vegetables are tender, about 15 minutes. Serve (eat as much oil as you can)!

PER SERVE:

**Calories**	**624**
**Fat**	**48.8 g (22.5 g saturated)**
**Protein**	**42.0 g**
**Fibre**	**2.5 g**
**Net carb**	**4.5 g**

Dinner
(Pan-American)

Bacon Avocado Wedges

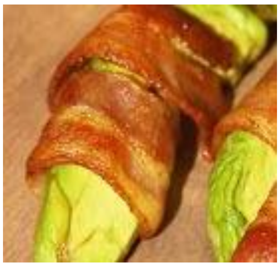

Makes 4 serves (12 wedges) total; 1 serve = 3 wedges.
Preparation time: MODERATE (15-30 minutes).

3 avocados
12 strips streaky bacon
1/4 cup full-fat sour cream (or Real Mayonnaise)

(1) Preheat oven to 200 C. Slice each avocado into four equally sized wedges.
(2) Wrap each wedge in bacon and place on a baking sheet.
(3) Bake 12-15 minutes, until the bacon is the way you want it.
(4) Top each wedge with 1 tsp sour cream and enjoy.

PER SERVE:

**Calories**	**588**
**Fat**	**55.5 g (13.9 g saturated)**
**Protein**	**15.6 g**
**Fibre**	**10.1 g**
**Net carb**	**3.0 g**

Salmon Con Salsa

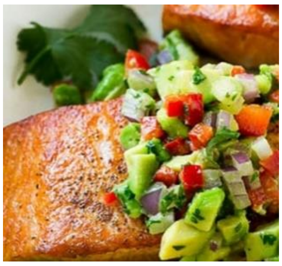

Makes 3 serves.
Preparation time: MODERATE (15-30 minutes).

Salsa:
1 avocado
1/4 red onion
1-2 tbsp fresh coriander, chopped
Juice from 2 limes

Salmon:
2 tbsp cold pressed extra virgin coconut oil
400 g salmon fillets
1 tsp (each) of cumin seeds, paprika, and onion powder
Salt and pepper to taste

(1) Mix together the sliced avocado, onion, and coriander in a bowl, then add lime juice and mix in well to create the salsa. Place the salsa in the fridge while you prepare the salmon.
(2) Melt the coconut oil in a pan over medium heat. Season the salmon fillets with the spices, salt, and pepper and place in the pan.
(3) Cook the fillets for 3-4 minutes on one side. Flip them over and cook the other side until the middle is pink (do not overcook).
(4) Remove from the pan onto a plate (include as much oil as you want). Top with the salsa and enjoy.

PER SERVE:

**Calories**	**431**
**Fat**	**33.4 g (12.2 g saturated)**
**Protein**	**27.9 g**
**Fibre**	**4.6 g**
**Net carb**	**1.6 g**

Chicken Fajita Bowl

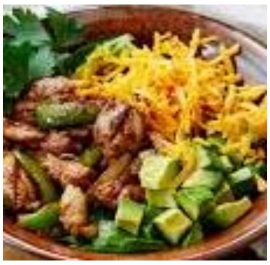

Makes 3 serves.
Preparation time: MODERATE (15-30 minutes).

1/2 head of lettuce
5 cherry tomatoes (or 1 tomato)
3 tbsp cold pressed extra virgin coconut oil
400 g chicken thighs
Salt and pepper to taste
1 tsp of (each) of oregano, garlic powder, and cumin seeds
1/2 red (or brown) onion
1/2 green capsicum
50 g cheddar (or your favourite) cheese
1 avocado
1/3 cup full-fat sour cream (or Keto Guacamole)
2-3 tbsp fresh coriander, chopped

(1) Finely slice the lettuce and add to a large bowl, then slice and mix in the tomato halves.
(2) Melt the coconut oil in a large pan over medium heat. Slice the chicken and add to the pan. After 2-3 minutes, add the salt, pepper, and seasonings and mix together. Cook 4-5 minutes.
(3) Slice the onion and capsicum length-wise and add to the chicken. Cook another 4-5 minutes, until chicken is cooked through, then mix the chicken, vegetables, and all oil into the bowl.
(4) Top the fajita bowl with the grated cheese, sliced avocado, sour cream, and coriander, and serve; mix it all together if you like!

PER SERVE:

**Calories**	**503**
**Fat**	**35.7 g (17.8 g saturated)**
**Protein**	**34.3 g**
**Fibre**	**7.4 g**
**Net carb**	**6.8 g**

Reverse Seared Steak

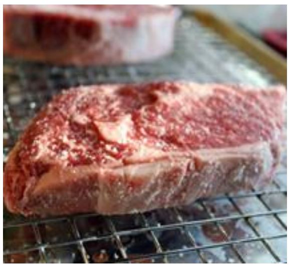

Makes 2 serves (2 steaks); 1 serve = 1 steak.
Preparation time: MODERATE (15-30 minutes; but nearly all oven time).

400 g scotch fillet steaks
Steak seasoning to taste
Salt and pepper to taste
1/4 cup cold pressed extra virgin coconut oil

(1) Preheat oven to 125 C. Put the steaks on a wire rack over top of a cookie sheet; do not remove the fat. Season heavily with seasoning, salt, and pepper on all sides of the meat.
(2) Bake for 30 minutes, then turn the steaks over and bake another 10-20 minutes, depending how well you like your steaks done. Remove and let the steaks rest a few minutes.
(3) Heat the oil in a large pan or pot over medium-high or high heat. There should be a solid film of oil covering the bottom. Wait until the oil is very hot, then sear the steaks 20-30 seconds on each side (you can sear the edges too if you want).
(4) Let the steaks rest 2-3 minutes and serve warm. Enjoy!

PER SERVE:

**Calories**	**609**
**Fat**	**45.1 g (23.0 g saturated)**
**Protein**	**49.5 g**
**Fibre**	**0 g**
**Net carb**	**3.0 g**

Steak Fajitas

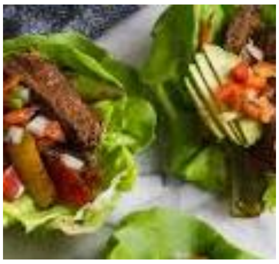

Makes 4 serves.
Preparation time: MODERATE (15-30 minutes).

3 tbsp cold pressed extra virgin coconut oil
3 capsicums (one red, one green, one yellow is best)
1/2 red (or brown) onion
Salt and pepper to taste
Steak seasoning
400 g scotch fillet steaks
4-8 large lettuce leaves
4 tbsp full-fat sour cream (or Keto Guacamole)

(1) Heat a large pan over medium heat, then melt 2 tbsp coconut oil in the pan. Slice up capsicums and onion, then add to the pan and season with salt and pepper. You will cook all the vegetables for 12-15 minutes total, but proceed to the next step when they start cooking.
(2) Heat another pan over medium heat, then melt 1 tbsp coconut oil in that pan. Season the steak with salt and pepper and the steak seasoning, then cook each side for 4-5 minutes, until seared.
(3) Remove both pans from the heat. Let the steak cool for 5 minutes, then slice it into slices of your desired size.
(4) Divide the vegetables and steak into four equal-sized portions. Wrap one portion of each in a large lettuce leaf (or two leaves, if you want to make it less likely to fall apart) and top each portion with 1 tbsp sour cream, then serve (can get messy, don't worry about it just enjoy)!

PER SERVE:

**Calories**	**392**
**Fat**	**28.8 g (16.0 g saturated)**
**Protein**	**26.4 g**
**Fibre**	**2.4 g**
**Net carb**	**7.0 g**

Tex-Mex Casserole

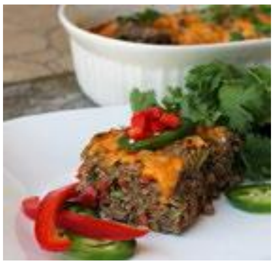

Makes 2 serves.
Preparation time: LONG (30-45 minutes; but nearly all oven time).

1/2 capsicum
2 stalks spring onions
2 tbsp fresh coriander, chopped
1 tbsp cold pressed extra virgin coconut oil, melted
240 g regular beef mince
1 egg
1 tsp (each) of cumin seeds, garlic salt, and oregano
Salt and pepper to taste
60 g cheddar cheese

(1) Preheat oven to 180 C. Slice the capsicum, spring onion, and coriander. Place these ingredients along with the coconut oil in a blender; pulse into a fine mixture.
(2) Combine the beef mince, egg, cumin, garlic salt, oregano, and salt and pepper in a large bowl. Add the blended mixture and mix it all together with your hands. Press the mixture into a baking dish, then top with grated cheddar cheese.
(3) Bake 30-40 minutes. Cool 10 minutes before serving.

PER SERVE:

**Calories**	**485**
**Fat**	**37.5 g (20.2 g saturated)**
**Protein**	**33.6 g**
**Fibre**	**0.6 g**
**Net carb**	**1.9 g**

Dinner
(East Asian)

Pushpa's Miti Fish

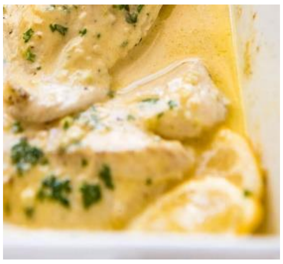

Makes 2 serves.
Preparation time: MODERATE (15-30 minutes).

1½ cups canned coconut cream
2 tbsp cold pressed extra virgin coconut oil, melted
Juice of 1 lemon
1-2 chilli, finely grated
4 stalks spring onions, finely sliced
1/2 tsp salt
400 g whitefish
1 head broccoli

(1) In a bowl, combine all ingredients to make the sauce (except for the fish and broccoli).
(2) If you have a steamer, steam the fish and sliced vegetables, then pour the sauce over the fish and vegetables (if you do not, go the next step).
(3) If you do not have a steamer, preheat the oven to 200 C and grease a baking pan with coconut oil. Place the fish in the pan, pour the sauce over the fish, and bake 20 minutes or until the fish is done. Boil the sliced vegetables in a pot and serve with the fish; goes well with cauliflower rice.

PER SERVE:

**Calories**	**556**
**Fat**	**40.6 g (26.9 g saturated)**
**Protein**	**50.6 g**
**Fibre**	**5.8 g**
**Net carb**	**5.8 g**

Sticky Chicken Stirfry

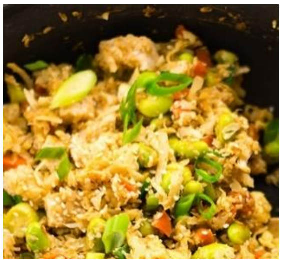

Makes 2 serves.
Preparation time: MODERATE (15-30 minutes).

400 g chicken thighs
1/4 cup Worcester (or low-sugar soy) sauce
1 cup grated cauliflower
3 mushrooms
3 stalks spring onion
1/2 capsicum
1/4 cup cold pressed extra virgin coconut oil
1 tbsp almond (or peanut) butter
1/4 cup plain Raglan/Cathedral Cove coconut yogurt
Salt and pepper to taste
2 tsp (each) of cumin seeds, coriander, and curry powder
120 g (1 bag) spinach (or kale) leaves

(1) Slice the chicken and marinate in the Worcester sauce while you prepare the cauliflower rice and vegetables.
(2) Grate the cauliflower into a large bowl, followed by the sliced mushrooms, spring onions, and capsicum; mix together and set aside.
(3) Melt the coconut oil in a large pan over medium heat. Fry the chicken in Worcester sauce for 10-12 minutes, then add the cauliflower rice and vegetables and fry another 2-3 minutes.
(4) Mix in the almond butter, coconut yogurt, salt, pepper, and spices. Fry for 2-3 minutes or until everything is melted and mixed well.
(5) Finally, add the spinach and fry for a final 2-3 minutes. Mix in the spinach and serve!

PER SERVE:

**Calories**	**622**
**Fat**	**46.1 g (31.5 g saturated)**
**Protein**	**44.6 g**
**Fibre**	**3.8 g**
**Net carb**	**6.9 g**

Thai Coconut Fish Curry

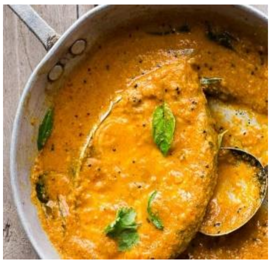

Makes 3 serves.
Preparation time: MODERATE (15-30 minutes).

1 tbsp cold pressed extra virgin coconut oil
400 grams whitefish
Salt and pepper to taste
4 tbsp butter (or ghee)
1½ cups canned coconut cream
2 tbsp red (or green) curry paste
1/2 cup fresh coriander, chopped
1/2 head cauliflower (or 1 head broccoli)

(1) Preheat oven to 200 C. Grease a medium-sized baking dish with the coconut oil.
(2) Place 2-4 fish pieces (depends on size) snuggly in the baking dish. Salt and pepper generously and place 1-2 tbsp of butter (depends on fish size) on top of each fish piece.
(3) Mix the coconut cream, curry paste, and coriander in a small bowl and pour this over the fish. Bake in the oven for 20 minutes, or until the fish is done.
(4) In the meantime, cut the cauliflower or broccoli into small florets and boil in lightly salted water for a couple of minutes. Serve with the fish (eat all the creamy curry) and enjoy with cauliflower rice.

PER SERVE:

**Calories**	**557**
**Fat**	**45.7 g (24.6 g saturated)**
**Protein**	**30.0 g**
**Fibre**	**4.8 g**
**Net carb**	**2.2 g**

Beef & Cashew Stirfry

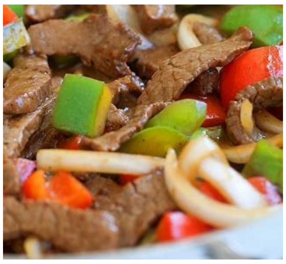

Makes 2 serves.
Preparation time: MODERATE (15-30 minutes).

4 tbsp cold pressed extra virgin coconut oil
2 cloves garlic (or 2 tsp crushed garlic)
1 carrot
1/4 brown onion
1 courgette
Salt and pepper to taste
1 tbsp Chinese 5 spice powder (or your favourite spices)
400 g diced beef
1/4 cup beef stock
1/2 cup canned coconut cream
1/4 cup roasted, salted cashews
3 tbsp fresh basil, chopped

(1) Heat 2 tbsp coconut oil in a wok over medium heat and cook the sliced garlic, carrot, and onion until fragrant.
(2) Mix in the courgette and salt and pepper; toss everything to combine. Set aside on a plate.
(3) Heat another 2 tbsp coconut oil in a pan over medium heat and brown the beef slices on both sides. Season with more salt and pepper, plus the Chinese 5 spice powder.
(4) Add the vegetables back in to the browned beef and mix it all together. Pour in the beef stock and coconut cream. Add the cashews and cook uncovered for 8 minutes.
(5) Lastly, add the basil and cook another 5 minutes. Enjoy!

PER SERVE:

**Calories**	**574**
**Fat**	**48.2 g (27.3 g saturated)**
**Protein**	**28.3 g**
**Fibre**	**2.0 g**
**Net carb**	**5.6 g**

Dinner
(South Asian)

Palak Paneer

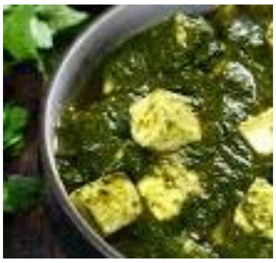

Makes 2 serves.
Preparation time: SHORT (<15 minutes).

1½ tbsp cold pressed extra virgin coconut oil
1/2 brown onion
2 cloves garlic (or 2 tsp crushed garlic)
1 tsp cumin seeds
120 g (1 bag) spinach leaves
Salt to taste
1/3 cup water
1½ tbsp unsalted butter
1/2 tsp (each) of turmeric, garam masala, coriander powder
1/3 cup canned coconut cream
200 g paneer (or haloumi) cheese

(1) Melt the coconut oil in a large pan over medium heat. Add the sliced onion, garlic, and cumin seeds and cook 2-3 minutes.
(2) Add the spinach leaves and salt, and cook another 2-3 minutes. Blend the spinach mixture and water in a blender until a spinach puree forms and set aside.
(3) Melt the butter in the pan. When melted, add the remaining spices and mix together.
(4) Pour in the spinach puree and cook 1-2 minutes. Then add the cream and cubed paneer and cook for another 1-2 minutes. Serve with cauliflower rice.

PER SERVE:

**Calories**	**571**
**Fat**	**51.3 g (37.0 g saturated)**
**Protein**	**24.7 g**
**Fibre**	**2.4 g**
**Net carb**	**4.5 g**

Butter Chicken

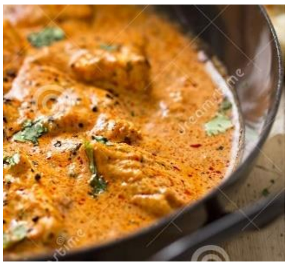

Makes 3 serves.
Preparation time: MODERATE (15-30 minutes).

Chicken Marinade:
1 cup plain Raglan/Cathedral Cove coconut yogurt
Juice from 1 lemon
1 tbsp turmeric
500 g chicken thighs

Sauce:
3 tbsp unsalted butter
1/2 brown onion
2 cloves garlic (or 2 tsp crushed garlic)
1/2 tsp ground cinnamon
2 tomatoes
Salt to taste
1/3 cup chicken stock
1/2 cup canned coconut cream
2 tbsp almond meal
2 tbsp fresh coriander, chopped

(1) Combine the marinade ingredients (except the chicken) in a large bowl. Once done, slice the thighs into bite-size pieces and mix into the bowl. Marinate while you prepare the rest.
(2) Melt the butter in a large pan over medium heat. Add the onion, garlic, and cinnamon and stir until the onion is browned. Next, add the sliced tomatoes and salt; cook 2-3 minutes.
(3) Add the chicken marinade; cook 6-7 minutes. Next add the stock, bring to a boil, reduce heat and simmer for another 6-7 minutes.
(4) Stir in the cream and almond meal and simmer another 8 minutes, or until chicken is fully cooked. Season with coriander and serve with cauliflower rice!

PER SERVE:

**Calories**	**556**
**Fat**	**40.5 g (26.4 g saturated)**
**Protein**	**36.1 g**
**Fibre**	**2.1 g**
**Net carb**	**9.1 g**

Keto Paneer Makhanwala

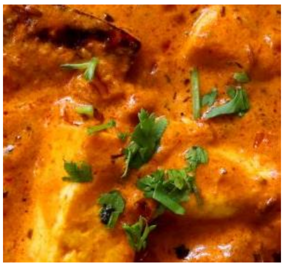

Makes 2 serves.
Preparation time: MODERATE (15-30 minutes).

1 tbsp unsalted butter
2 tbsp cold pressed extra virgin coconut oil
1/2 brown onion
1 tsp cumin seeds
1 tsp (each) of turmeric and coriander
Salt to taste
2 tbsp tomato puree
1/2 cup water
200 g paneer (or haloumi) cheese
1/3 cup canned coconut cream
2-3 tbsp fresh coriander, chopped

(1) Heat a large pan over medium and add the butter and 1 tbsp coconut oil. Add the onion and cumin seeds and cook until the onion is translucent.
(2) Mix in the rest of the spices and salt, and cook for 2 minutes. Then add the tomato puree and water, lower the heat, and simmer 7-8 minutes.
(3) While the sauce is simmering, melt 1 tbsp of coconut oil in another pan, and fry the cubed cheese until golden brown.
(4) Add the cheese (plus coconut oil), cream, and coriander to the onion and spices and mix well. Simmer 1-2 minutes, then serve with cauliflower rice!

PER SERVE:

**Calories**	**576**
**Fat**	**51.7 g (38.0 g saturated)**
**Protein**	**23.3 g**
**Fibre**	**2.0 g**
**Net carb**	**6.4 g**

Salmon Curry

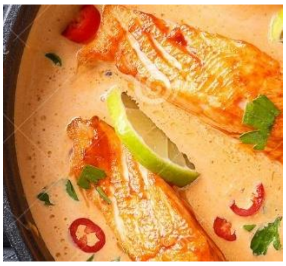

Makes 2 serves.
Preparation time: MODERATE (15-30 minutes).

Spice Mix:
2 tsp (each) of curry powder, turmeric, and coriander
1 tsp (each) of cumin seeds and ground cinnamon
Salt and pepper to taste

Salmon Sauce:
360 g salmon fillets
2 tbsp cold pressed extra virgin coconut oil
1/2 brown onion
2 cloves garlic (or 2 tsp crushed garlic)
2 tomatoes
3/4 cup canned coconut cream
Juice from 1 lime
1 tsp almond (or peanut) butter
2 tbsp fresh coriander, chopped

(1) Combine the spice mix ingredients in a small bowl. Cube the fillets and set aside on a plate.
(2) Heat the coconut oil in a large pan over medium heat. Add the sliced onion and garlic; saute for 2-3 minutes. Add the spice mix; saute another 2-3 minutes. Mix continuously to prevent burning.
(3) Add the sliced tomatoes, cream, lime juice, and almond butter. Bring to a simmer.
(4) Once simmering, turn to low heat and add the cubed fillets and fresh coriander. Cover and cook 8-12 minutes or until the fillets flake with a fork (do not overcook). Serve with cauliflower rice.

PER SERVE:

**Calories**	**587**
**Fat**	**43.7 g (21.9 g saturated)**
**Protein**	**38.7 g**
**Fibre**	**3.4 g**
**Net carb**	**6.6 g**

Chicken Korma

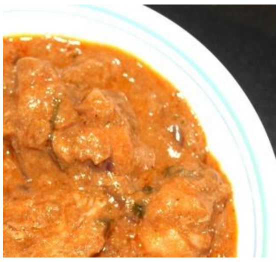

Makes 2 serves.
Preparation time: MODERATE (15-30 minutes).

400 g chicken thighs
1/4 cup plain Raglan/Cathedral Cove coconut yogurt
2 cloves garlic (or 2 tsp crushed garlic)
2 tbsp cold pressed extra virgin coconut oil
1 tsp (each) of curry powder and turmeric
Pinch of pepper
1/4 brown onion
1/2 capsicum
1/2 cup chicken stock
1 cup canned coconut cream
1 tomato
1 Lebanese (small) cucumber

(1) Chop the chicken into small chunks and mix it into a bowl with the yogurt and minced garlic to marinate as you prepare the rest.
(2) Heat the coconut oil in a large pan over low heat. Once melted, mix in the curry, turmeric, and pepper. Stir intermittently from here on so that the spices don't stick to the pan.
(3) Turn heat to medium, then add the marinated chicken, onion, and capsicum. Cook 7-8 minutes, until no raw chicken bits are showing.
(4) Stir in the stock and cream, then simmer 8 minutes.
(5) Stir in the sliced tomato and cucumber, simmer 4 minutes, and enjoy with cauliflower rice.

PER SERVE:

**Calories**	**583**
**Fat**	**40.2 g (27.4 g saturated)**
**Protein**	**44.3 g**
**Fibre**	**3.8 g**
**Net carb**	**10.5 g**

Indian Eggplant

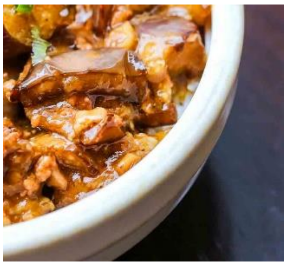

Makes 2 serves.
Preparation time: LONG (30-45 minutes; but nearly all oven time).

2 eggplants
4 tbsp cold pressed extra virgin coconut oil
1 tsp cumin seeds
1/2 brown onion
2 cloves garlic (or 2 tsp crushed garlic)
1/2 inch ginger (optional)
1 tomato
1 tsp (each) of turmeric, garam masala, and paprika
Salt and pepper to taste
100 g paneer (or haloumi) cheese
1-2 tbsp fresh coriander, chopped

(1) Preheat oven to 180 C. Make four slits lengthwise in each eggplant and drizzle 1 tbsp melted coconut oil over top to fully cover each. Place on a baking sheet and roast in the oven for 30-40 minutes, flipping halfway.
(2) While the eggplants are baking, add 2 tbsp coconut oil to a pot over medium heat. Add the cumin and onion, then saute for 10 minutes.
(3) Add the sliced garlic, ginger, tomato, and spices and mix well. Cover the pan with a lid and cook 10-12 minutes, then remove the lid and fry another 2-3 minutes.
(4) The eggplant should be ready now. Remove from the oven and cool, remove the skin and chop the flesh. Stir the eggplant into the fry; cook another 5 minutes. Garnish with coriander and serve with cauliflower rice.

PER SERVE:

**Calories**	**518**
**Fat**	**42.3 g (33.7 g saturated)**
**Protein**	**15.6 g**
**Fibre**	**13.7 g**
**Net carb**	**13.3 g**

Lamb Curry

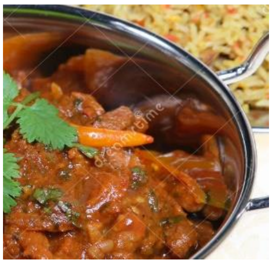

Makes 3 serves.
Preparation time: LONG (30-45 minutes).

2 tbsp cold pressed extra virgin coconut oil
400 g diced lamb (or beef)
1/2 brown onion
2 stalks celery
2 cloves garlic (or 2 tsp crushed garlic)
1 tsp (each) of garam masala and turmeric
1 tbsp unsalted butter
1½ cups canned coconut cream
1/4 cup water
1/2 tsp salt
1 carrot
Juice from 1/2 lemon
2-3 tbsp fresh coriander, chopped

(1) Melt the coconut oil in a large pan over medium-high heat. Add the lamb and stir until slightly browned, about 4-5 minutes.
(2) Add the sliced onion and celery and cook another 2-3 minutes, until the vegetables are soft. Bring the heat down to medium. Add the minced garlic, spices, and butter and cook another 2-3 minutes, until the lamb is cooked through.
(3) Add the sliced carrot, coconut cream, water, and salt. Bring to a simmer, and cook for 10-12 minutes.
(4) Sprinkle with lemon juice and coriander before serving with cauliflower rice.

PER SERVE:

**Calories**	**529**
**Fat**	**44.4 g (26.1 g saturated)**
**Protein**	**26.6 g**
**Fibre**	**2.8 g**
**Net carb**	**3.5 g**

Vegetable & Cashew Korma

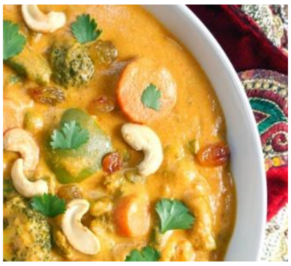

Makes 3 serves.
Preparation time: LONG (30-45 minutes; but nearly all soaking time).

1 cup cashews
1½ cups canned coconut cream
2 cloves garlic (or 2 tsp crushed garlic)
1/2 inch ginger (optional)
1 tbsp curry powder
1 tsp turmeric
3 tbsp cold pressed extra virgin coconut oil
1/4 brown onion
1/2 head broccoli
1/2 cup water
Salt to taste

(1) Stir the cashews into the coconut cream and soak for 20-30 minutes in a bowl.
(2) Pour the cashews and cream into a blender. Add the sliced garlic, ginger, curry powder, and turmeric. Blend until the mixture is smooth.
(3) In a large pot (or pan), heat the coconut oil over medium-high heat. Add the sliced onion and cook until soft, then add the sliced broccoli a to the pot and pour the blended mixture over top, followed by the water and salt.
(4) Cover the pot and bring to a low boil. Reduce heat to low-medium and simmer 15 minutes, stirring occasionally so the sauce doesn't burn. Add more water if the sauce is too thick.
(5) Add more seasonings and cook another 2-3 minutes before serving. Enjoy with cauliflower rice.

PER SERVE:

**Calories**	**540**
**Fat**	**49.0 g (23.9 g saturated)**
**Protein**	**10.2 g**
**Fibre**	**4.4 g**
**Net carb**	**12.5 g**

Chicken Curry Drumsticks

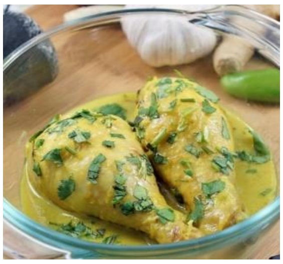

Makes 2 serves.
Preparation time: LONG (30-45 minutes; but nearly all stove time).

1/2 brown onion
1 clove garlic (or 1 tsp crushed garlic)
2 tbsp cold pressed extra virgin coconut oil
1 tsp turmeric
4 chicken drumsticks (skin on)
1/2 cup canned coconut cream
1/2 cup water
1 tsp salt
1 tbsp fresh coriander, chopped

(1) Slice the onion. Mince the garlic. Place them both in a blender and blend together.
(2) Heat the coconut oil in a pan over medium heat. Saute the blended mixture in the coconut oil for 3-4 minutes.
(3) Add the turmeric. Saute another 3-4 minutes, then add the chicken, mix well, and transfer everything to a large pot.
(4) Pour in the coconut cream and water and mix well. Add the salt and simmer while covered about 30 minutes, or until the chicken is cooked.
(5) Sprinkle fresh coriander over the top and serve with cauliflower rice!

PER SERVE:

**Calories**	**426**
**Fat**	**32.1 g (19.3 g saturated)**
**Protein**	**29.3 g**
**Fibre**	**1.6 g**
**Net carb**	**3.2 g**

Side Dishes

Real Mayonnaise

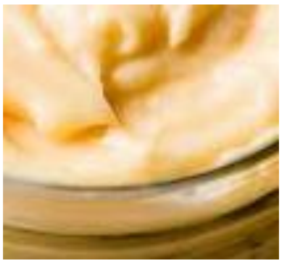

Makes about 3/4 cup of mayonnaise.
Preparation time: SHORT (<15 minutes).

1 egg yolk
Juice from 1/2 to 1 lemon (how lemony is your choice)
1 tsp mustard (Dijon is best)
1 tsp white wine or apple cider vinegar
1/4 tsp salt
1 tsp water
3/4 cup cold pressed extra virgin coconut oil, melted
1 tbsp sour cream

(1) Separate the egg yolk and egg white (an easy way to do this is to crack the egg, then move the yolk back and forth between each half-shell while letting the white drip away).
(2) Place the yolk, lemon juice, mustard, vinegar, salt, and water in a bowl and whisk together.
(3) Slowly drip the oil into the bowl, whisking constantly to let the oil incorporate. Do not drip it in too fast or the mayo will not be thick enough.
(4) When the mayo is thick enough, mix in the sour cream, and serve (it will keep in the fridge for 3-4 days).

PER SERVE:

**Calories**	**1,459**
**Fat**	**168.0 g (142.6 g saturated)**
**Protein**	**2.7 g**
**Fibre**	**0 g**
**Net carb**	**0.6 g**

Keto Guacamole

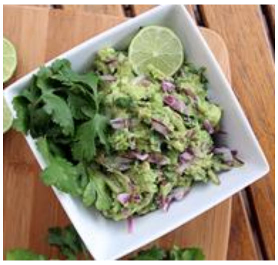

Makes about 3/4 cup of guacamole.
Preparation time: SHORT (<15 minutes).

2 avocados
1/3 medium red onion
2 tbsp pre-made salsa
Juice from 1 lime
Salt and pepper to taste
2 tbsp fresh coriander, chopped

(1) Cut the avocados in half and spoon out into a bowl.
(2) Slice the onion and add to the bowl, followed by the salsa, lime juice, and salt and pepper. Mash everything together; use a fork for the avocado pieces if necessary.
(3) Fold in the coriander carefully (you don't want the guacamole to be too smooth).
(4) Serve with more fresh coriander and lime on the side if you want, and enjoy.

PER SERVE:

**Calories**	**662**
**Fat**	**59.0 g (8.6 g saturated)**
**Protein**	**8.2 g**
**Fibre**	**27.2 g**
**Net carb**	**11.0 g**

Cauliflower Rice

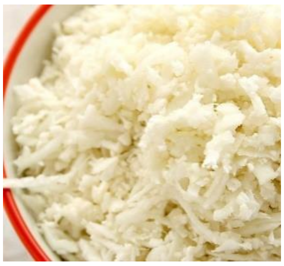

Makes 2 side-serves.
Preparation time: SHORT (<15 minutes).

1 cup grated cauliflower
1 tbsp unsalted butter (or extra virgin olive oil)
1 tbsp cold pressed extra virgin coconut oil
Salt and pepper to taste

(1) Grate the cauliflower into a large bowl.
(2) Heat the butter and coconut oil in a large pan over medium heat. Once melted, add the cauliflower and sprinkle salt over top. Cook 4-6 minutes.
(3) Turn off the heat and add more salt if needed. Serve.

PER SIDE-SERVE:

**Calories**	**131**
**Fat**	**13.8 g (11.8 g saturated)**
**Protein**	**1.1 g**
**Fibre**	**1.4 g**
**Net carb**	**1.5 g**

Indian Cauliflower Rice

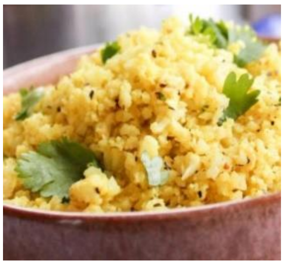

Makes 2 side-serves.
Preparation time: SHORT (<15 minutes).

1 cup grated cauliflower
1/2 brown onion
1 tbsp unsalted butter
1 tbsp cold pressed extra virgin coconut oil
Salt to taste
1/2 tsp (each) of ginger powder, coriander, garam masala, and cumin seeds
2-3 tbsp fresh coriander, chopped

(1) Grate the cauliflower and slice the onion into a large bowl.
(2) Heat the butter and oil in a large pan over medium heat. Once melted, add the cauliflower and onion, and sprinkle salt over the vegetables. Cook 4-6 minutes.
(3) Stir in the spice powders over 1-2 minutes, then turn off the heat.
(4) Stir in the coriander and add more salt if needed. Serve.

PER SIDE-SERVE:

**Calories**	**135**
**Fat**	**12.7 g (9.6 g saturated)**
**Protein**	**1.4 g**
**Fibre**	**1.8 g**
**Net carb**	**4.0 g**

Marinated Olives

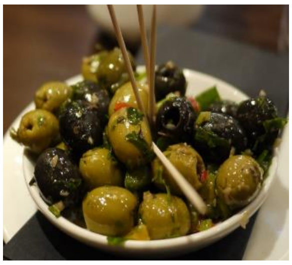

Makes 2 side-serves.
Preparation time: SHORT (<15 minutes).

200 g olives (seed-in is best)
Juice from 1/2 lemon
2 tbsp olive oil
1 tsp coriander
Salt and pepper to taste

(1) Drain and place the olives in a bowl.
(2) Squeeze the lemon juice into a separate bowl. Add the olive oil first followed by the coriander, salt, and pepper to create the dressing.
(3) Pour the dressing over the olives and let marinate for a while - the longer, the better. Serve!

PER SERVE:

**Calories**	**267**
**Fat**	**28.8 g (3.3 g saturated)**
**Protein**	**1.1 g**
**Fibre**	**3.3 g**
**Net carb**	**1.3 g**

Worcester Brussels Sprouts

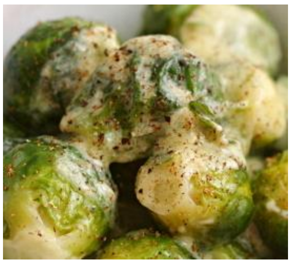

Makes 2 side-serves.
Preparation time: SHORT (<15 minutes).

2 tbsp cold pressed extra virgin coconut oil
2 tbsp unsalted butter
10 Brussels sprouts
Salt and pepper to taste
2 tbsp canned coconut cream
2 tsp Worcester sauce

(1) Place a frying pan over medium-high heat and add the oil and butter.
(2) Wash the Brussels sprouts, cut them lengthwise, and add to the pan. Cook 3-4 minutes, or until sprouts are golden brown. Add salt and pepper.
(3) Pour the cream and Worcester sauce over the Brussels sprouts at the end and cook for another 30 seconds. Serve immediately (eat the oil and cream) and enjoy!

PER SIDE-SERVE:

**Calories**	**274**
**Fat**	**26.6 g (20.2 g saturated)**
**Protein**	**1.5 g**
**Fibre**	**3.8 g**
**Net carb**	**2.5 g**

Warm Kale Salad

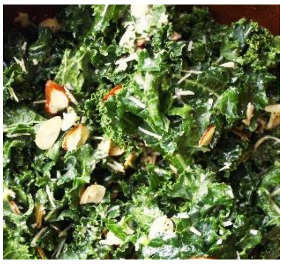

Makes 2 side-serves.
Preparation time: SHORT (<15 minutes).

2 tbsp canned coconut cream
1 tbsp olive oil
1 tsp mustard
Salt and pepper to taste
1 tbsp unsalted butter
120 g (1 bag) kale (or spinach) leaves
15 g goat feta cheese
2 tbsp almonds

(1) Combine the cream, oil, mustard, salt, and pepper in a mug. Stir thoroughly and set aside.
(2) Place a frying pan over medium heat and melt the butter.
(3) Slice the kale into small bite-size pieces and add it to the pan. Saute the kale on medium heat so it turns a nice colour (if using spinach, until it starts to wilt), but no more than that. Add salt and pepper to taste.
(4) When done, place the kale in a bowl and pour the dressing over it. Stir thoroughly and add crumbled feta cheese and almonds (sliced or whole) on top. Serve.

PER SIDE-SERVE:

**Calories**	**212**
**Fat**	**20.3 g (7.0 g saturated)**
**Protein**	**6.2 g**
**Fibre**	**2.7 g**
**Net carb**	**1.9 g**

Buttered Broccoli

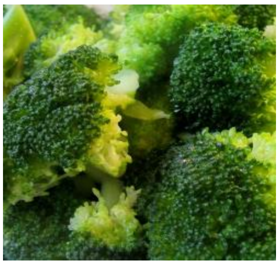

Makes 1 side-serve.
Preparation time: SHORT (<15 minutes).

1/2 head of broccoli
1 tbsp unsalted butter

(1) Boil some water in a pot.
(2) Chop the broccoli into florets and add them to the water.
(3) Cook until desired doneness is reached; this can be anywhere from 1-4 minutes.
(4) Remove to a plate and add the butter on top. Serve.

PER SIDE-SERVE:

**Calories**	**141**
**Fat**	**11.5 g (7.4 g saturated)**
**Protein**	**3.1 g**
**Fibre**	**3.0 g**
**Net carb**	**5.0 g**

Keto Asparagus

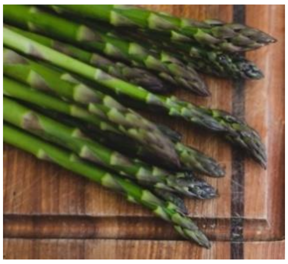

Makes 2 side-serves.
Preparation time: SHORT (<15 minutes).

1 bunch asparagus
1 tbsp cold pressed extra virgin coconut oil
1 tbsp unsalted butter
2 cloves garlic (or 2 tsp crushed garlic)

(1) Wash the asparagus and separate the stalks.
(2) Boil some water in a pot and cook asparagus 2-3 minutes.
(3) Drain the asparagus and cool in cold water.
(4) Heat the coconut oil, butter, and garlic in a pan over low-medium heat.
(5) Fry asparagus 2-3 minutes, or until browning; be careful not to overcook. Enjoy (eat all the oil and butter)!

PER SIDE-SERVE:

**Calories**	**127**
**Fat**	**12.7 g (9.6 g saturated)**
**Protein**	**2.0 g**
**Fibre**	**1.9 g**
**Net carb**	**1.7 g**

Garlic Spinach

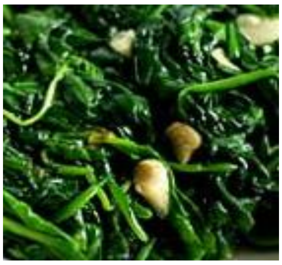

Makes 1 side-serve.
Preparation time: SHORT (<15 minutes).

1 tbsp cold pressed extra virgin coconut oil
1 tbsp olive oil
1 clove garlic (or 1 tsp crushed garlic)
60 g (1/2 bag) spinach (or kale) leaves
Salt and pepper to taste

(1) Bring a pan to medium heat on the stove. Once hot, add the coconut oil.
(2) Add the minced garlic to the pan and saute 1-2 minutes.
(3) Add the spinach to the pan and cook until wilted; mix it in well with the oil.
(4) Season with salt and pepper, and serve (don't leave any oil in the pan).

PER SIDE-SERVE:

**Calories**	**252**
**Fat**	**27.2 g (3.5 g saturated)**
**Protein**	**1.8 g**
**Fibre**	**1.4 g**
**Net carb**	**0.8 g**

Asian Broccoli Slaw

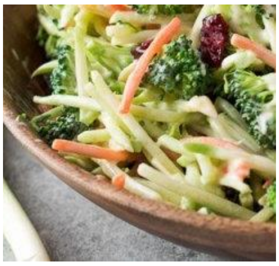

Makes 2 side-serves.
Preparation time: SHORT (<15 minutes).

2 tbsp cold pressed extra virgin coconut oil
225 g (1/2 bag) broccoli slaw
60 g (1/2 bag) spinach (or kale) leaves
1 tbsp coconut amino sauce
Salt and pepper to taste
2 tbsp plain Raglan/Cathedral Cove coconut yogurt
1 tbsp sunflower seeds

(1) Melt coconut oil in a large pan over medium-high heat.
(2) Discard any pre-packaged slaw dressing and place the broccoli slaw in the pan. Cover and cook 6-7 minutes.
(3) Uncover, then stir in the spinach, coconut aminos, salt, and pepper. Cook 2-3 minutes.
(4) Turn off the heat, stir in the coconut yogurt and sunflower seeds and serve.

PER SIDE-SERVE:

**Calories**	**229**
**Fat**	**18.8 g (14.8 g saturated)**
**Protein**	**4.7 g**
**Fibre**	**3.8 g**
**Net carb**	**10.1 g**

Lemon Roasted Broccoli

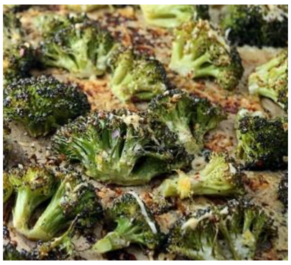

Makes 2 side-serves.
Preparation time: MODERATE (15-30 minutes; but nearly all oven time).

1/2 head of broccoli
2 tbsp extra virgin olive oil
1 tbsp fresh basil, chopped
1 clove garlic (or 1 tsp crushed garlic)
1/2 tsp salt
Juice from 1 lemon
30 g parmesan cheese

(1) Preheat the oven to 220 C. Cut the broccoli into small florets and lay them on a baking tray greased with coconut oil.
(2) Season the broccoli with the olive oil, basil, minced garlic, salt, lemon zest, and lemon juice.
(3) Sprinkle grated parmesan cheese over the broccoli and place in the oven 15-20 minutes. When done, remove from the oven and serve.

PER SIDE-SERVE:

**Calories**	**201**
**Fat**	**17.4 g (4.3 g saturated)**
**Protein**	**6.7 g**
**Fibre**	**1.5 g**
**Net carb**	**3.7 g**

Vegetable Medley

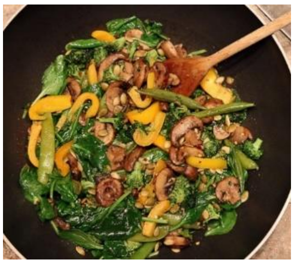

Makes 3 side-serves.
Preparation time: MODERATE (15-30 minutes).

Dressing:
2 tbsp olive oil
2 tsp mustard (optional)

Medley:
2 tbsp cold pressed extra virgin coconut oil
2 cloves garlic (or 2 tsp crushed garlic)
4 mushrooms
1/2 head of broccoli
1/2 green capsicum
1/4 cup pumpkin seeds
Salt and pepper to taste
60 g (1/2 bag) spinach (or kale) leaves

(1) Combine the olive oil and mustard (if using) in a mug; set aside.
(2) Prepare all the vegetables beforehand (important, so you can move quickly later) by slicing them into bite-size pieces; make sure this is done before proceeding to the next step.
(3) Heat the coconut oil in a pan on high heat. Once hot, add the minced garlic and mushrooms; make sure the mushrooms soak up as much oil as possible. Cook 1-2 minutes.
(4) In order, mix in the broccoli, capsicum, and pumpkin seeds. Season with salt and pepper. Cook another 1-2 minutes.
(5) Turn off the heat and add the spinach leaves; after 2-3 minutes, stir in the leaves (they should be slightly wilted). Transfer to a plate, pour the oil and mustard dressing over top, and serve.

PER SIDE-SERVE:

**Calories**	**327**
**Fat**	**32.8 g (11.6 g saturated)**
**Protein**	**4.8 g**
**Fibre**	**2.4 g**
**Net carb**	**4.4 g**

Dessert

Hot Cocoa

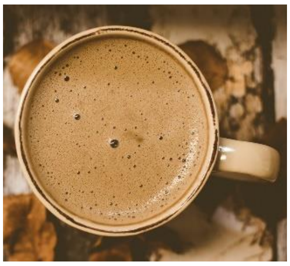

Makes 1 serve.
Preparation time: SHORT (<15 minutes).

2/3 cup water
1 tbsp cold pressed extra virgin coconut oil, melted
1/3 cup canned coconut cream
1 tsp vanilla extract
2 tsp dark cocoa powder
1 tsp Natvia

(1) Bring the water to a boil.
(2) Meanwhile, place all the other ingredients in a blender.
(3) Add the boiling water to the blender and pulse until smooth.
(4) Pour into a mug and enjoy.

PER SERVE:

**Calories**	**256**
**Fat**	**23.5 g (18.5 g saturated)**
**Protein**	**5.3 g**
**Fibre**	**1.2 g**
**Net carb**	**3.5 g**

Chocolate Shake

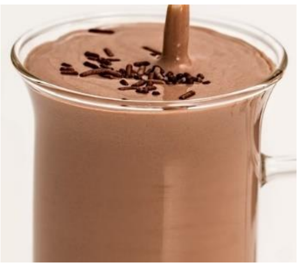

Makes 1 serve.
Preparation time: SHORT (<15 minutes).

1/2 tbsp cold pressed extra virgin coconut oil, melted
1 tbsp chia seeds
1/2 tbsp dark cocoa powder
1/2 tbsp Natvia
1 cup canned coconut cream

(1) Place all ingredients into a blender.
(2) Blend until very smooth. Enjoy!

PER SERVE:

**Calories**	**421**
**Fat**	**36.7 g (25.3 g saturated)**
**Protein**	**7.5 g**
**Fibre**	**8.1 g**
**Net carb**	**4.0 g**

Dark Chocolate Pudding

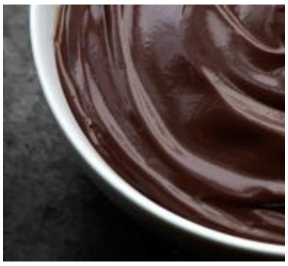

Makes 3 serves.
Preparation time: SHORT (<15 minutes).

2 avocados
1/4 cup dark cocoa powder
3 tbsp cold pressed extra virgin coconut oil, melted
1/2 cup canned coconut cream (or water)
1/4 cup Natvia
1 tsp pure vanilla extract

(1) Finely slice the avocados. Place all the ingredients into a blender.
(2) Blend well, until all the avocado chunks have disappeared and you have a smooth chocolate pudding. You can add a little less (or more) cream or water if you want a thicker (or runnier) pudding.
(3) Serve into three small bowls and enjoy!

PER SERVE:

**Calories**	**456**
**Fat**	**37.7 g (17.5 g saturated)**
**Protein**	**11.5 g**
**Fibre**	**9.0 g**
**Net carb**	**8.8 g**

Berries & Cream

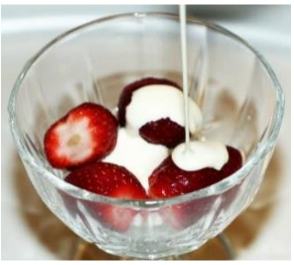

Makes 1 serve.
Preparation time: SHORT (<15 minutes).

3 strawberries <u>OR</u> 12 raspberries <u>OR</u> 12 blackberries <u>OR</u> 18 blueberries
3/4 cup canned coconut cream
1 tsp vanilla extract

Preparation:
(1) Slice up the berries, if needed. Place them in a small bowl.
(2) Mix in the cream with vanilla extract and serve.

PER SERVE:

**Calories**	**185**
**Fat**	**17.3 g (12.0 g saturated)**
**Protein**	**2.3 g**
**Fibre**	**3.6 g**
**Net carb**	**2.9 g**

Chocolate Cupcakes

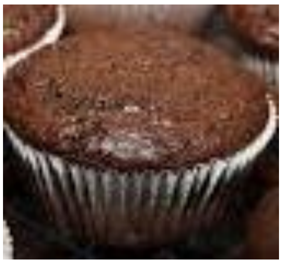

Makes 6 serves (6 cupcakes) total; 1 serve = 1 cupcake.
Preparation time: MODERATE (15-30 minutes; but nearly all oven time).

1/4 cup cold pressed extra virgin coconut oil, melted
2 tbsp unsalted butter, melted
2 eggs
1/4 cup canned coconut cream
1 tsp pure vanilla extract
1/4 cup dark cocoa powder
1/4 cup coconut flour
1 tbsp chia seeds
1/3 cup Natvia
1 tsp baking powder
40 g (4 small squares) Lindt 90% dark chocolate (or Dark Chocolate Pudding)

(1) Preheat the oven to 175 C. Combine the oil, butter, eggs, cream, and vanilla extract in a large bowl.
(2) Combine all the dry ingredients in a separate bowl, then add them to the wet ingredients in the large bowl. Stir until it is a smooth batter.
(3) Grease six muffin cups with coconut oil or butter. Place the batter into the cups and bake for 20 minutes.
(4) Melt the Lindt chocolate in a microwave or small pot on the stove, then use a spoon to drizzle it over the cupcakes (or spread the pudding over top, if using that). Cool for 10 minutes, then serve.

PER SERVE:

**Calories**	**324**
**Fat**	**24.0 g (17.0 g saturated)**
**Protein**	**10.2 g**
**Fibre**	**8.2 g**
**Net carb**	**5.7 g**

Cinnamon Butter Cookies

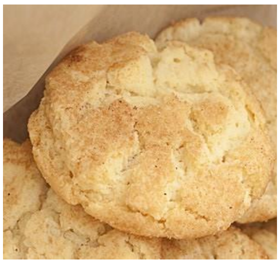

Makes 6 serves (12 cookies) total; 1 serve = 2 cookies.
Preparation time: MODERATE (15-30 minutes; but nearly all oven time).

2 cups almond meal
1/3 cup unsalted butter, melted
2 tbsp cold pressed extra virgin coconut oil, melted
1 egg
2 tsp vanilla extract
2 tsp ground cinnamon
2 tbsp Natvia

(1) Preheat oven to 150 C. Place all ingredients in a large bowl and mix until well combined.
(2) Grease a baking tray with coconut oil or butter. Roll the mixture into 12 balls and place on the tray. Bake in the oven 5 minutes.
(3) Remove from the oven and press the balls down with a fork. Return to the oven and bake another 18-20 minutes.
(4) Remove from the oven, cool for 5 minutes, and serve!

PER SERVE:

**Calories**	**395**
**Fat**	**38.1 g (12.5 g saturated)**
**Protein**	**10.0 g**
**Fibre**	**1.2 g**
**Net carb**	**2.4 g**

Chocolate Pecan Cookies

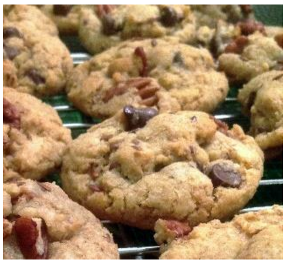

Makes 4 serves (8 cookies) total; 1 serve = 2 cookies.
Preparation time: MODERATE (15-30 minutes; but nearly all oven time).

1/2 cup almond meal
1 cup pecan halves (or walnuts)
1 tsp baking powder
1/4 cup Natvia
1/2 tsp salt
1 tbsp unsalted butter, melted
1 tbsp cold pressed extra virgin coconut oil, melted
1 egg white
40 g (4 small squares) Lindt 90% dark chocolate

(1) Preheat the oven to 175 C. Combine dry ingredients in a blender and blend until pecans are a coarse ground.
(2) Add the butter, coconut oil, and egg white and blend a little more, until the cookie dough is wet and sticky, but still chunky.
(3) Grease a baking tray with coconut oil or butter. Using a spoon, place eight rounded cookie balls onto the tray. Bake 15-20 minutes.
(4) Melt the Lindt chocolate in a microwave or small pot on the stove, then use a spoon to drizzle it over the cookies. Cool for 10 minutes, then place in the fridge 30 minutes before serving.

PER SERVE:

**Calories**	**377**
**Fat**	**36.5 g (10.3 g saturated)**
**Protein**	**9.0 g**
**Fibre**	**3.4 g**
**Net carb**	**4.4 g**

Berry & Dark Chocolate Cups

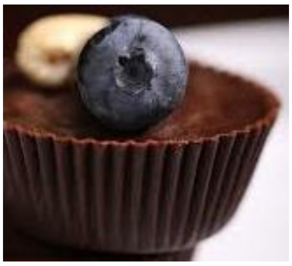

Makes 1 serve (2 chocolate cups) total; 1 serve = 2 chocolate cups.
Preparation time: LONG (30-45 minutes, but nearly all fridge time).

30 g (3 small squares) Lindt 90% dark chocolate
2 tbsp cold pressed extra virgin coconut oil
1/2 tsp Natvia
8 raspberries OR 12 blueberries
8 almonds

(1) Break the dark chocolate squares into little pieces and melt them with the coconut oil in a double boiler (or a heat-proof bowl over a small saucepan filled with water) on medium heat.
(2) Once melted, stir in the Natvia and spoon the hot chocolate mixture into two muffin cups.
(3) Add four raspberries (or six blueberries) and four almonds to each muffin cup.
(4) Refrigerate for at least 45 minutes before serving. Enjoy!

PER SERVE:

**Calories**	**478**
**Fat**	**48.7 g (33.9 g saturated)**
**Protein**	**5.3 g**
**Fibre**	**5.9 g**
**Net carb**	**6.8 g**

Chocolate Chip Cookies

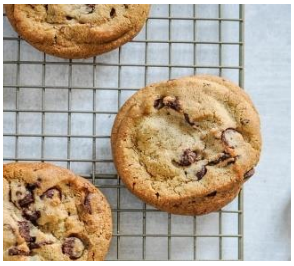

Makes 6 serves (12 cookies) total; 1 serve = 2 cookies.
Preparation time: VERY LONG (>45 minutes; but nearly all oven and fridge time).

2 cups almond meal
1/2 cup cold pressed extra virgin coconut oil, melted
1 egg
2 tsp vanilla extract
1/2 tsp baking soda
3 tbsp Natvia
80 g (8 small squares) Lindt 90% dark chocolate

(1) Preheat oven to 150 C. Place all ingredients (except chocolate) in a large bowl and mix until well combined.
(2) Break or crush the chocolate into small pieces, then gently fold into the cookie mixture.
(3) Grease a baking tray with coconut oil or butter. Roll mixture into 12 balls and place on the tray. Bake in the oven 20-25 minutes.
(4) Cool for 10 minutes, then place in the fridge 1 hour before serving.

PER SERVE:

**Calories**	**502**
**Fat**	**48.9 g (22.3 g saturated)**
**Protein**	**11.2 g**
**Fibre**	**2.9 g**
**Net carb**	**4.7 g**

Keto Cheesecake

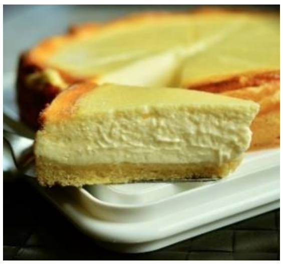

Makes 12 serves.
Preparation time: VERY LONG (>45 minutes; but nearly all oven and fridge time).

Crust:
1/2 cup almond meal
1/2 cup coconut flour
1/4 cup dessicated (or thread) coconut
1/4 cup cold pressed extra virgin coconut oil, melted
1/4 cup unsalted butter, melted

Filling:
240 g cream cheese, softened
480 g full-fat sour cream, room temperature
1/4 cup Natvia
1 tbsp vanilla extract
3 eggs

Toppings (PER SERVE):
2 strawberries <u>OR</u> 8 raspberries <u>OR</u> 8 blackberries <u>OR</u> 12 blueberries

(1) Preheat oven to 150 C. Grease a baking dish with coconut oil. In a small bowl, mix together the crust ingredients. Press the mix into the bottom of the baking dish and place in the fridge while you make the filling.
(2) In a large bowl, beat the cream cheese and sour cream together, followed by the Natvia and vanilla. Beat the eggs in last, one egg at a time. Mix until smooth.
(3) Spread the filling over the crust. Bake the cheesecake in the oven for 1 hour.
(4) Remove and place in the fridge for at least 5 hours (ideally, overnight). Place berry toppings as you like, and serve.

PER SERVE:

**Calories**	**363**
**Fat**	**30.9 g (20.2 g saturated)**
**Protein**	**8.3 g**
**Fibre**	**7.2 g**
**Net carb**	**3.8 g**

Lazy Days (Eating In)

Sometimes, you may want to just eat some simple food at home, maybe even right off the shelf. If this is the case, that is fine as long as the food is high-fat, adequate-protein, and low-carb. The **following list of foods** can be eaten in any combination, on any day.

Cold pressed, extra virgin coconut oil (the best, use in any way you like)
Canned coconut cream (Palm Island is good)
Eggs any style (poached is best)
Bacon
Any fish (salmon, sardines, tuna etc) without batter or pre-packaged sauce
Avocados
Coffee or tea (no milk or sugar, take coffee black, or add cream or coconut oil)
Olives
Nuts and seeds
Home St Keto Bread (can buy from Pak n Save)
Vinegar
Olive oil, avocado oil, and macadamia nut oil
Butter and ghee
Coconut yogurt (stick to Raglan or Cathedral Cove, the others have too much sugar)
Any leafy greens cooked in or covered in any of the above oils, butter, or ghee

Lazy Days (Eating Out)

Sometimes, it is good to eat out, perhaps at a restaurant or social gathering. This is ok as long as you stick to “allowable” foods. Stick to the **following list of meals** when eating out, and try to add at least two tablespoons of healthy oil or butter to every meal.

Breakfast

Breakfast is relatively easy to stay ketogenic in most restaurants. Please **avoid bread, hash browns, potatoes, muesli, porridge, cereals, milk, fruits, waffles, or pancakes** for breakfast (lots of sugar in all of them). Example breakfast options include:

Eggs any style (poached is best, especially with Hollandaise sauce)
Bacon, salmon, and sausages (sausages not the best option, but ok)
Avocados, olives
Coffee or tea (no milk or sugar, take coffee black, or add coconut oil or cream)
Mushrooms (especially in cream)
Omelettes (ask for no potato or bread)
Green vegetables and leafy greens salads
Tomatoes (no more than one tomato)

Lunch

Lunch is probably best done by sticking to salads, although there are also other options. Please **avoid breads (including sandwiches and burgers), pastas, sodas, wedges, chips, potatoes, rice, or nachos** for lunch. Example lunch options include:

Avocados, olives
Chicken, beef, lamb, or seafood salad (no breads, croutons, or potato)
Fish with vegetables (ask for extra butter - no breads, croutons, potato, or rice)
Meat or vegetable soup (no breads, potato, or rice)
Sashimi (not sushi as it contains rice)
Steak (ask for extra butter - no breads, croutons, potato, or rice)
Green vegetables and leafy green salads (ask for extra olive oil)

Dinner and Dessert

Dinner is similar to lunch; if you follow the lunch rules, you will be fine. Mediterranean restaurants (Italian, Greek, Turkish, French etc) are good choices; ask for extra olive oil and vinegar and use as much as you want of either. Indian restaurants are ok if you **avoid all bread and rice**, and stick to the fattiest curries (such as meat and vegetable kormas and madras).

Desserts at restaurants invariably contain too much sugar. Stick to cheese plates, **avoiding all other desserts** (or just go home and have some dessert from the diet plan).
